# Stable peptide-assembled nanozyme mimicking dual antifungal actions

**DOI:** 10.1038/s41467-024-50094-6

**Published:** 2024-07-05

**Authors:** Ye Yuan, Lei Chen, Kexu Song, Miaomiao Cheng, Ling Fang, Lingfei Kong, Lanlan Yu, Ruonan Wang, Zhendong Fu, Minmin Sun, Qian Wang, Chengjun Cui, Haojue Wang, Jiuyang He, Xiaonan Wang, Yuan Liu, Bing Jiang, Jing Jiang, Chenxuan Wang, Xiyun Yan, Xinzheng Zhang, Lizeng Gao

**Affiliations:** 1 Nanozyme Laboratory in Zhongyuan, School of Basic Medical Sciences, Zhengzhou University, Zhengzhou, 450001 China; 2 CAS Engineering Laboratory for Nanozyme, National Laboratory of Biomacromolecules, Institute of Biophysics, Chinese Academy of Sciences, Beijing, 100101 China; 3 Xishan People’s Hospital of Wuxi City, Wuxi Branch of Zhongda Hospital Southeast University, Wuxi, 214105 Jiangsu China; 4 National Laboratory of Biomacromolecules, CAS Center for Excellence in Biomacromolecules, Institute of Biophysics, Chinese Academy of Sciences, Beijing, 100101 China; 5 State Key Laboratory of Common Mechanism Research for Major Diseases, Department of Biophysics and Structural Biology, Institute of Basic Medical Sciences Chinese Academy of Medical Sciences, School of Basic Medicine Peking Union Medical College, Beijing, 100005 China; 6 Nanozyme Laboratory in Zhongyuan, Henan Academy of Innovations in Medical Science, Zhengzhou, Henan 450052 China

**Keywords:** Nanobiotechnology, Antifungal agents, Peptides, Bioinspired materials

## Abstract

Natural antimicrobial peptides (AMPs) and enzymes (AMEs) are promising non-antibiotic candidates against antimicrobial resistance but suffer from low efficiency and poor stability. Here, we develop peptide nanozymes which mimic the mode of action of AMPs and AMEs through de novo design and peptide assembly. Through modelling a minimal building block of IHIHICI is proposed by combining critical amino acids in AMPs and AMEs and hydrophobic isoleucine to conduct assembly. Experimental validations reveal that IHIHICI assemble into helical β-sheet nanotubes with acetate modulation and perform phospholipase C-like and peroxidase-like activities with Ni coordination, demonstrating high thermostability and resistance to enzymatic degradation. The assembled nanotubes demonstrate cascade antifungal actions including outer mannan docking, wall disruption, lipid peroxidation and subsequent ferroptotic death, synergistically killing >90% *Candida albicans* within 10 min on disinfection pad. These findings demonstrate an effective de novo design strategy for developing materials with multi-antimicrobial mode of actions.

## Abstract (web summary)

Natural antimicrobial peptides and enzymes are good candidates for application but suffer from low stability. Here, the authors report on biomimetic self-assembling peptides which mimic both antimicrobial peptide and enzyme functionality, demonstrating application against fungal infection.

## Introduction

Antimicrobial resistance (AMR) is an emerging crisis causing antibiotics failure to infectious diseases^1–3^. It is estimated that AMR may result in more than 10 million deaths in 2050. WHO is therefore calling for more research to develop novel antimicrobial strategies and drugs. Many non-antibiotic alternatives have been developed to combat AMR crisis, among which antimicrobial peptides (AMPs) represent a promising and potent candidate^4,5^. Natural AMPs, also called host defense peptides (HDPs), are short peptides generally constituted by 12–50 amino acids, performing protection from infection in innate immune response found among all classes of life forms^6,7^. AMPs have been demonstrated to kill bacteria, viruses, and fungi through destabilizing biological membranes or forming transmembrane channels^8^. AMPs perform high biocompatibility and avoid causing drug resistance, thus showing great potential for clinical translation as novel therapeutic agents. However, due to long sequence of amino acids, natural AMPs are not very stable to environmental stimuli such as protease degradation, high temperature, and ionic salts, and hold high cost for synthesis and storage, which limits their potential for practical use^9,10^. To overcome this, many artificial strategies have been developed to synthesize and improve AMPs, among which peptide assembly into specific nanostructure has drawn a lot of attention recently^11–13^. AMPs assembly into nanostructure not only enhances the stability but also improves valence as a single assembled unit contains multiple AMPs^14,15^. However, the composition and conformation of natural AMPs are hard to drive ordered self-assembly at nanoscale.

Besides AMPs, antimicrobial enzymes (AMEs) also demonstrate great potential to AMR crisis. Some natural enzymes, such as lysozyme or deoxyribonuclease, can hydrolyze the cell structure of bacteria and thus exhibit excellent antibacterial performance as enzymes-based antibacterials (Enzybiotics)^16^. AMEs are mainly classified into three categories: peptidoglycan hydrolases, proteases, and nuclease, which are highly specific, effective, and fast-acting and thus avoid to induce drug resistance. However, AMEs have low resistance to protease degradation and cause immunogenicity because of their protein nature, which seriously limits their clinical transformation^17^. Moreover, the widespread application of AMEs in industrial processes is considered restricted due to poor stability of enzymes under unfriendly conditions (such as pH and temperature). To overcome these limitations, artificial enzymes able to mimic the activities of AMEs provide an alternative strategy. Recently, nanozymes, a kind of nanomaterials or nanostructures with enzyme-like activity, have attracted much attention as a new generation of artificial enzymes^18^. Compared with natural enzymes, nanozymes show the characteristics of being more stable, economical, and practical. It has been reported that nanozymes with peroxidase (POD)-like or oxidase-like activity could produce a great number of reactive oxygen species (ROS) to kill bacteria^19,20^. Furthermore, nanozymes with hydrolase-like or DNase-like activity also prevent the spread of drug resistance by degradation of bacterial drug resistance genes^21,22^. Consequently, antimicrobial nanozymes have been recognized as an alternative of AMEs to combat AMR challenge, which are termed as nanozybiotics^23,24^. Importantly, recent studies demonstrate that peptide self-assembly can be used to develop supramolecular nanozymes for biomimetic catalysis^25^. Therefore, design peptide-based nanozymes to mimic AMEs may overcome the limitations of AMEs and extend their potency for antimicrobial therapy.

The fundamental rule that both AMPs and AMEs depend on critical amino acids to perform the antimicrobial and catalytic activity provides the rational for designing such nanozymes. Specific amino acid-rich peptides, such as Histatins, perform antimicrobial functions depending on histidine (His) as active site^26–28^. In addition, it has been reported that cysteine (Cys) helps in enhancement of the stability of AMPs and provides resistance against chemical or proteolytic degradation^29^. Coincidentally, His and Cys residues are critical in active center of AMEs for catalytically killing bacteria, including oxidases, peroxidases and hydrolases. It has been recognized that amphipathic His has the highest frequency ( ~ 20%) in active center of enzymes, although its frequency in all proteins is just 5%^30^. Single His could be modulated into nano-assembly to perform peroxidase-like activity^31^. In addition, Cys is the second most frequency in the active center of enzymes. Therefore, His and Cys as basic amino acids are primary choices for de novo design of bioactive materials.

In this work, we develop a de novo strategy of combining His and Cys into one peptide able to assemble into nanozymes with dual features of antimicrobial peptide and antimicrobial enzymes (AMPANs). To facilitate nanoscale assembly, we further introduce isoleucine (Ile) isoleucine which is the most hydrophobic amino acid modulating protein aggregation^32^. We firstly use AlphaFold2 and molecular dynamics (MD) simulations to rapidly determine the optimal sequence and length for the structures of self-assembling^33^. Through experimental validations, a heptapeptide of IHIHICI is successfully designed with the ability to form an ordered nanostructure and act as a nanozyme to perform phospholipase C (PLC)-like activity and peroxidase-like activity. As expected, such heptapeptide nanozyme performs dual AMPs-like and AMEs-like actions to binding fungal surface, disrupting cell wall and inducing lipid peroxidation-based ferroptosis, demonstrating a rapid antifungal activity toward *Candida albicans* (*C. albicans*).

## Results

### De novo design assisted by AlphaFold2

**Fig. 1:**
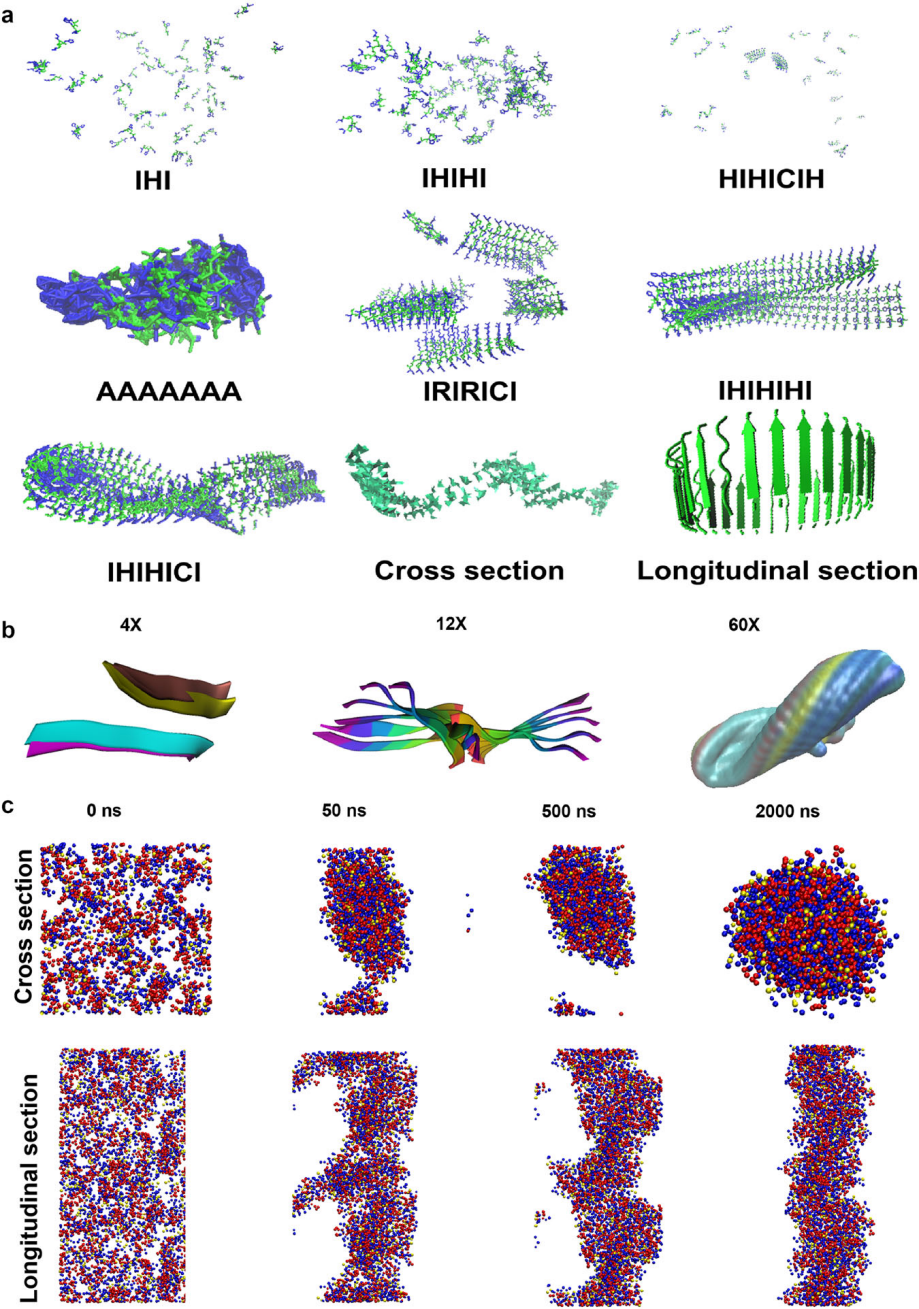
AlphaFold2-aided de novo design of peptide assembly. **a** Possible three-dimensional structures of various polypeptide sequences predicted by AlphaFold2. **b** Simulation of the aggregation of IHIHICI. **c** MD simulations of dynamic assembly into macroscopic structure of IHIHICI.

To achieve the peptide able to assembly into nanostructure with nanozyme feature, we first used the computer simulation software AlphaFold2 in combination with MD and DFT to simulate the spontaneous assembly of peptides with permutation and combination of His, Ile and Cys. We describe a computational and experimental protocol, which screens large numbers of candidate peptides to obtain the aimed AMPANs. In principle, a peptide with alternating hydrophilic and hydrophobic amino groups is prone to self-assembly. Using AlphaFold2 prediction (Fig. 1a), we first screened peptide sequences according to permutation and combination of His and Ile and the length of the amino acids. The simulation results demonstrated that the comprising alternating Ile and His residues is able to form an ordered structure when the length is up to 7 amino acids (heptapeptide). It should be noted that both carboxyl and amine ends of the polypeptide should be Ile rather than His. In addition, peptides less than 7 amino acids in length are susceptible to loss of helical pitch and inhomogeneity during assembly. The heptapeptide IHIHICI showed the potential to form β-sheet structure and assembly into large object (cross section and longitudinal section of IHIHICI structure). These results were confirmed by experimental characterization of peptide morphologies using transmission electron microscopy (TEM). Neither IHI, IHIHI nor HIHICH could form ordered nanostructure (Supplementary Fig. 1a–c). In comparison, the heptapeptide sequence of IHIHIXI could form typical nanofibers (Supplementary Fig. 1d and Supplementary Fig. 2). In addition, the amino acid at position 6 can be flexible to be other amino acids. Whether the position 6 is Ala (A) Met (M), or Phe (F), the heptapeptides were all able to form similar nanofibers (Supplementary Figs. 1d and 2). This flexible position provides the site to introduce Cys. In comparison, the combination of His with Ile is critical to form fibril nanostructure for the heptapeptide. If the His was replaced by Arg (R), Cys (C), Pro (P), the heptapeptides (IRIRICI, ICICICI, or IPIPICI) could not form typical nanostructure (Supplementary Figs. 2 and 3). These results were consistent with the prediction by AlphaFold2 (Fig. 1a and Supplementary Fig. 4). In particular, pLDDT scores from AlphaFold2 were collected to assess the tendency of peptides to undergo self-assembly^34^. As shown in Supplementary Table 1, it is observed that the scores for IRIRICI, IHIHIHI, IHIHICI, and IHIHIFI are relatively high. These peptide assembly trends were further confirmed with Coarse-grained molecular dynamics (GCMD)^35^, among which the aggregation propensity (AP) values showed IHIHICI has the best assembly possibility (Supplementary Figs. 5 and 6).

Furthermore, AlphaFold2 predictions indicated that peptides could exhibit a layered extension on a horizontal plane (Supplementary Fig. 7). Therefore, we speculated that the optimized peptide sequence was the heptapeptide of IHIHICI. To further confirm it, AlphaFold2, molecular dynamics (MD), and density functional theory (DFT) were used in combination to further understand peptide polymerization of IHIHICI. As shown in Fig. 1b, AlphaFold2 prediction demonstrated that heptapeptide IHIHICI were able to gradually polymerize and self-assemble into tubular structures containing helical motifs (4× = 4 peptide molecules, 60× = 60 peptide molecules). GCMD simulations demonstrated self-assembly of the macroscopic structure of IHIHICI over time. In the first 0.05 μs, randomly dispersed IHIHICI rapidly aggregated into small irregular clusters which then fused together and formed a large aggregate at 0.5 μs. This cluster gradually converted to a tube-like structure by 2 μs (Fig. 1c, Supplementary Fig. 8). The driving forces mainly contain hydrogen bondings, π-π stacking interactions, hydrophobic interactions, van der Walls (vdW) interactions and electrostatic interactions (Supplementary Figs. 9–11). Single-point energy calculations indicated that the vdW weak interactions (green region) among atoms in close contact between peptides were significant (Supplementary Fig. 12). DFT demonstrated that IHIHICI was capable of curved growth due to the presence of chiral carbon and hydrogen bonding interactions (Supplementary Movie 1 and 2), although the theoretical size did not match the actual observed size as the computer model cannot comprise many heptapeptide molecules due to computational limitations (Supplementary Fig. 13).

**Fig. 2:**
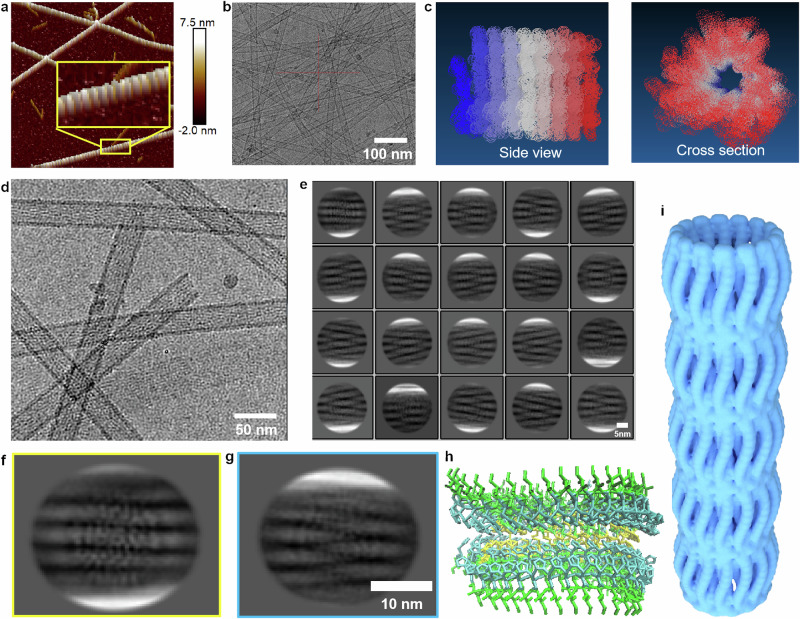
High-resolution characterization of nanostructure by Ni-IH-7 assembly. **a** Helical morphology of Ni-IH-7 assembly characterized by AFM. The inserted image was the magnified area. **b** Tubular nanostructure of Ni-IH-7 assembly characterized by TEM. **c** Schematic diagram of the simulation of helical nanotubes. **d**–**g** Cryo-EM and two-dimensional classification analysis of Ni-IH-7 assembly. **h** The predicted intermolecular structure according to Cryo-EM characterization of Ni-IH-7 assembly using AlphaFold2. **i** Three-dimensional structural diagram of Ni-IH-7 nanotubes. Three times each experiment was repeated independently with similar results. Representative images are shown.

To endow the biomimetic catalysis of IHIHICI (IH-7) nanostructure as nanozymes, we introduced metal salts which can coordinate with imidazole residue of His to form active site similar to those in natural enzymes. According to AlphaFold2 predication, the β-sheet conformation might be a characteristic structure for IH-7 assembly. We then introduced circular dichroism (CD) to characterize the secondary structure of IH-7 in the presence of metal salts. In general, IHIHICI was able to assemble into various secondary structure conformations in varying metal ion solutions, with the negative peak ranging from 198 nm for random coils to 217 nm for β-sheets. By screening a range of metal chloride and acetate salts, we found that nickel acetate (Ni(Ac)_2_) facilitated IHIHICI to form typical secondary structure of β-sheets (Supplementary Figs. 14–16) and the intact nanofiber (termed Ni-IH-7) (Fig. 2a). In addition, CD spectra showed that the pre-assembled Ni-IH-7 nanostructure showed typical β-sheet conformation but could be disrupted by FeCl_3_ (Supplementary Fig. 17). The critical aggregation concentration (CAC) of IH-7 detected with Nile red dye was around 10–15 µM, which was close to the range for the critical micelle concentration (CMC) detected using pyrene probe (Supplementary Fig. 18). The nuclear magnetic resonance (NMR) further confirmed the assembly of IH-7 in the presence of Ni(Ac)_2_ according to the disappearance of characteristic peaks of free peptide (Supplementary Fig. 19). The UV-VIS spectra demonstrated that Ni-IH-7 exhibited a peak at 292 nm (Supplementary Fig. 20), indicating the formation of a metal coordination (Ni–O/N)^36^, which was further validated by XPS characterization (Supplementary Fig. 21). Furthermore, Fourier-transform infrared (FTIR) demonstrated that the Ni-IH-7 had a highly ordered β-sheet structure, while pure IH-7 had a random coil structure in water (Supplementary Fig. 22). Further analysis for the amide I regions of FTIR showed that the ratio for β-sheet and random coil in Ni-IH-7 was 41.7% and 0, respectively, while those structures in IH-7 were 12.0% and 36.1%, respectively (Supplementary Figs. 23 and 24 and Supplementary Tables 2 and 3). The scanning tunneling microscope (STM) characterization of Ni-IH-7 nanotubes further confirmed that β-sheet parallel structures were primarily formed between different IHIHICI, and the distance between peptide chains ranged from 4–5 Å, consistent with the simulated structure (Supplementary Fig. 25). These results demonstrated that IH-7 assembly into nanostructure is characterized with β-sheet structure and effectively driven by Ni(Ac)_2_ to achieve metal coordination. Given the uniformity and symmetry of the nanotube structure, Ni(Ac)_2_-induced self-assembly of the Ni-IH-7 was selected as the focus of the present study.

### IHIHICI assembly into β helix nanotube in the presence of Ni(Ac)_2_

To further verify the assembly of the IHIHICI, we used atomic force microscopy (AFM), Cryo-EM and computer simulation characterize the nanoscale structure and analyze the potential interactions of Ni-IH-7 samples. As shown in Fig. 2a and Supplementary Fig. 26, AFM images showed that Ni-IH-7 formed a long and straight nanofiber with typical helix pattern on the surface like a tightly wound spring. Cryo-EM showed that Ni-IH-7 had hollow structure and thus it should be nanotube with a diameter of approximately 24 nm and a wall depth of approximately 4 nm (Fig. 2b and d). Physical adsorption testing with both N_2_ and Ar showed that the Ni-IH-7 nanotubes had the gaps of approximately 1 nm within the tubular walls (Supplementary Fig. 27). The helical profile (side view) and tubular structure (section view) were proposed in the schematics as shown in Fig. 2c. Two-dimensional classification were used to provide Finer structure of nanotubes (Fig. 2e). In the yellow and blue circles of Figs. 2f and g 5–6 laterally grown spiral helical nanoribbons have been observed between walls (the bright white part represents the wall of nanotube). The nanoribbons between the top and bottom appear to have a winding effect at the spiral parts. The plausible structure of Ni-IH-7 was predicated with using AlphaFold2 based on two-dimensional classification analysis of Cryo-EM image. As shown in Fig. 2h, the imidazole groups of His were parallel inside between tow helical ribbons and disulfide bonds may be formed to connect the two ribbons. In addition, no free thiol was detected and most of Cys was in oxidized state in Ni-iH-7 nanotubes (Supplementary Figs. 28 and 29), indicating the formation of disulfide bonds (S–S) in the assembled Ni-IH-7 nanotubes. Based on the above results, we reconstructed a three-dimensional structural diagram of the β-helix nanotubes (Fig. 2i). Both the transverse and longitudinal directions of the nanotubes exhibit helical characteristics. The pitch of the nanotube (the length of winding part) and nanoribbon (or the length of unit) are both about 10 nm. The parallel distance between the nanoribbons is approximately 2 nm (Fig. 2f), which means the interaction of peptides (or catalytic active) mainly existed in the winding part of nanoribbon. The Young’ s modulus was measured as 7.49 GPa by Ni-IH-7 (Supplementary Fig. 30), which means the formation of soft nanotube. In addition, the powder X-ray diffraction (PXRD) spectra showed that in Ni-IH-7 samples IHIHICI monomers were mainly aggregated to ordered nanotubes with the patterned spectra (Supplementary Fig. 31).

### Acetate modulates heptapeptide assembly

**Fig. 3:**
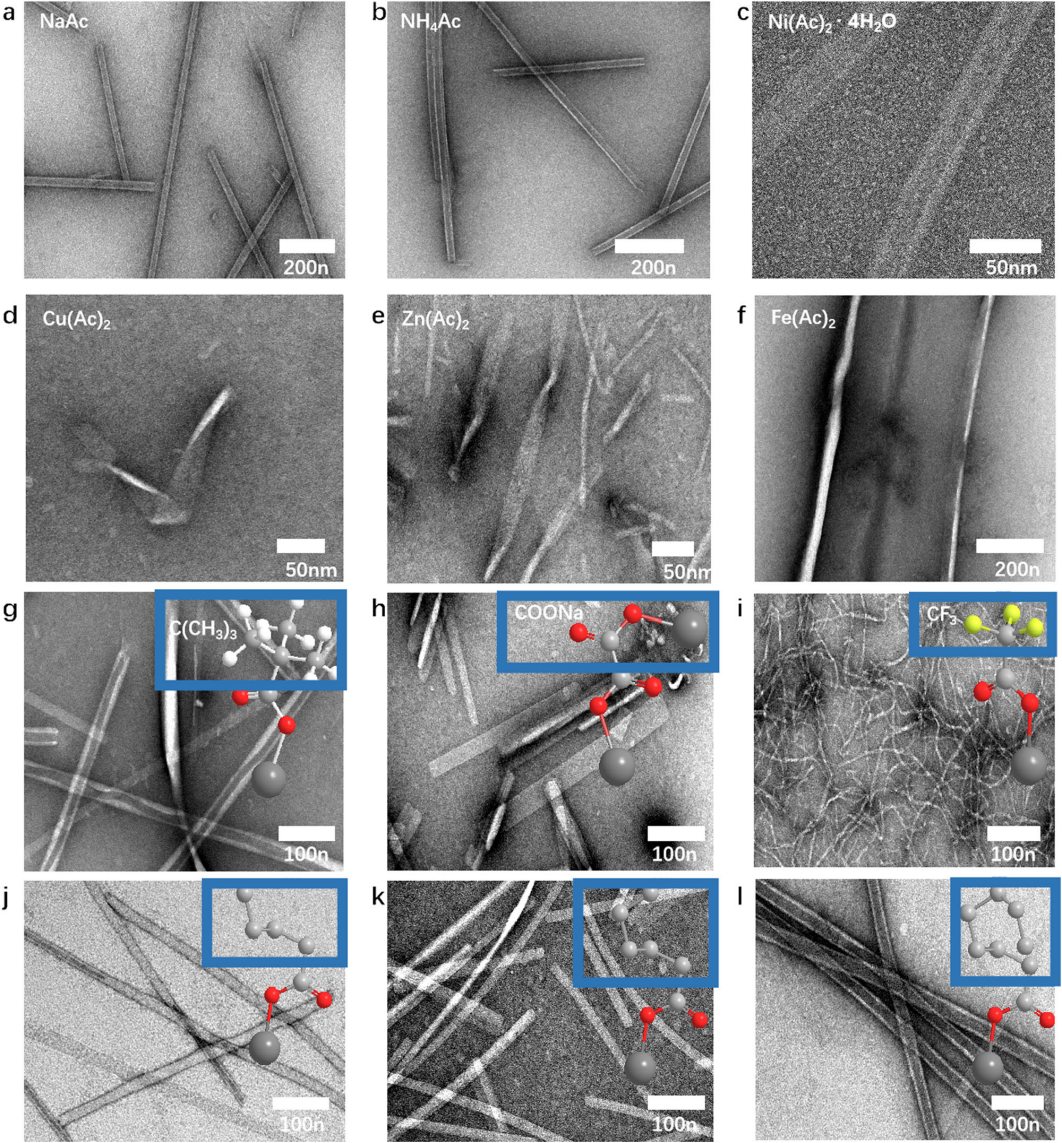
Acetate modulates IHIHICI assembly into tubular nanostructure. **a**–**c** Negative-stain TEM image of IHIHICI assembly with NaAc, NH_4_Ac, and Ni(Ac)_2_·4H_2_O, respectively. **d**–**f** Negative-stain TEM image of IHIHICI assembly with Cu(Ac)_2_, Zn(Ac)_2_, and Fe(Ac)_2_, respectively. **g**–**i** Negative-stain TEM image of IHIHICI assembly with sodium trimethyl acetate, sodium oxalate, and sodium trifluoroacetate, respectively. **j**–**l** Negative-stain TEM image of IHIHICI assembly with sodium valerate, sodium caproate, and sodium caprylate, respectively. Three times each experiment was repeated independently with similar results. Representative images are shown.

To further confirm the role of Ni(Ac)_2_ in Ni-IH-7 assembly, we investigated the influence of acetate salts on nanotubular morphology using negative staining TEM and CD. Firstly, although Ni(Ac)_2_ endowed the heptapeptide to form nanotubes, other nickel salts, such as nickel carbonate, basic nickel carbonate, nickel hypophosphite or nickel formate only induced the heptapetide to form irregular morphology (Supplementary Table 4). Conversely, acetate salts, such as sodium acetate, ammonium acetate endowed the heptapeptide (IH-7) into straight tubular morphology similar to that with Ni(Ac)_2_ (Fig. 3a–c). These results demonstrated that acetate is critical for IHIHICI to assemble into straight tubular nanostructure. In addition, such assembly process was independent on the environmental temperature (Supplementary Fig. 32). Of noted, other heptapeptide, such as IRIRICI (IR-7), retained assembly in nanoribbons rather than nanotubes whether under extended sonication time or increased concentration of Ni(Ac)_2_ (Supplementary 33). Secondly, if nickel is replaced with other metal ions in acetate salts, the morphology was also affected. As shown in Fig. 3d–f, folded nanotubes in a V-shape were observed in the presence of Cu(Ac)_2_ (Fig. 3d) and the addition of Zn(Ac)_2_ resulted in twisting of the nanotubes (bright white areas in peptides are torsion tangles) leading to the formation of a thinner helical structure (Fig. 3e), while Fe completely disrupted the nanotube structure (Fig. 3f). CD spectra further confirmed that IH-7 under Cu(Ac)_2_ and Fe(Ac)_2_ conditions primarily exhibited irregular coiling conformation, while under Zn(Ac)_2_, IH-7 underwent a transition from a mixture of alpha helix and β-sheet to primarily β-sheet, especially with longer sonication time (Supplementary Figs. 34 and  35). These results demonstrated that Ni(Ac)_2_ is optimal to facilitate IH-7 assembly into ordered nanostructure.

Finally, to further investigate the influence of acetate moiety (CH_3_COO^−^) of nanotube formation by, the molecules with different H substituents (CH_3_) were used for heptapeptide self-assembly. The addition of C(CH_3_)_3_COO^−^ (Sodium trimethyl acetate) caused inward curling of nanotube wall, thereby decreasing the diameter of the tube in contrast to the use of CH_3_COO^−^ (Fig. 3g), When the CH_3_ group was replaced with COO^−^ (Sodium oxalate), wider nanorods (21–32 nm) were formed and the nanotube wall disappeared. The combination as COO^−^ with heptapeptides led to the formation of nanorods (the inner part of the nanotube, ~16 nm), with the CH_3_ group appearing to promote curling of the rod to form the nanotube wall. Accordingly, wider nanorods were formed due to the lack of a CH_3_ group and an increased amount of COO^-^ (Fig. 3h). CF_3_COONa (Sodium trifluoroacetate)-heptapeptides formed thinner tangled filaments of solid nanorods (Fig. 3i). In comparison, the addition of CH_3_CH_2_CH_2_CH_2_COO^−^ (Sodium valerate), CH_3_CH_2_CH_2_CH_2_CH_2_COO^−^ (Sodium caproate) and CH_3_ CH_2_CH_2_CH_2_CH_2_CH_2_CH_2_COO^−^ (Sodium caprylate) allowed to maintain essential tubular morphology (Fig. 3j–l). These results demonstrated that electron-withdrawing group in acetate suppressed the formation of nanotubular morphology and electron donating carbon chain facilitated acetate to promote nanotube formation. Taken together, acetate moiety is critical for the IH-7 assembly into tubular nanostructure, while the metal ion also affect the assembly, among which Ni(Ac)_2_ is optimal recipe for IH-7 assembly into intact nanotubes (Ni-IH-7).

### Coordination interactions of Ni(Ac)_2_ facilitates nanotube assembly

**Fig. 4:**
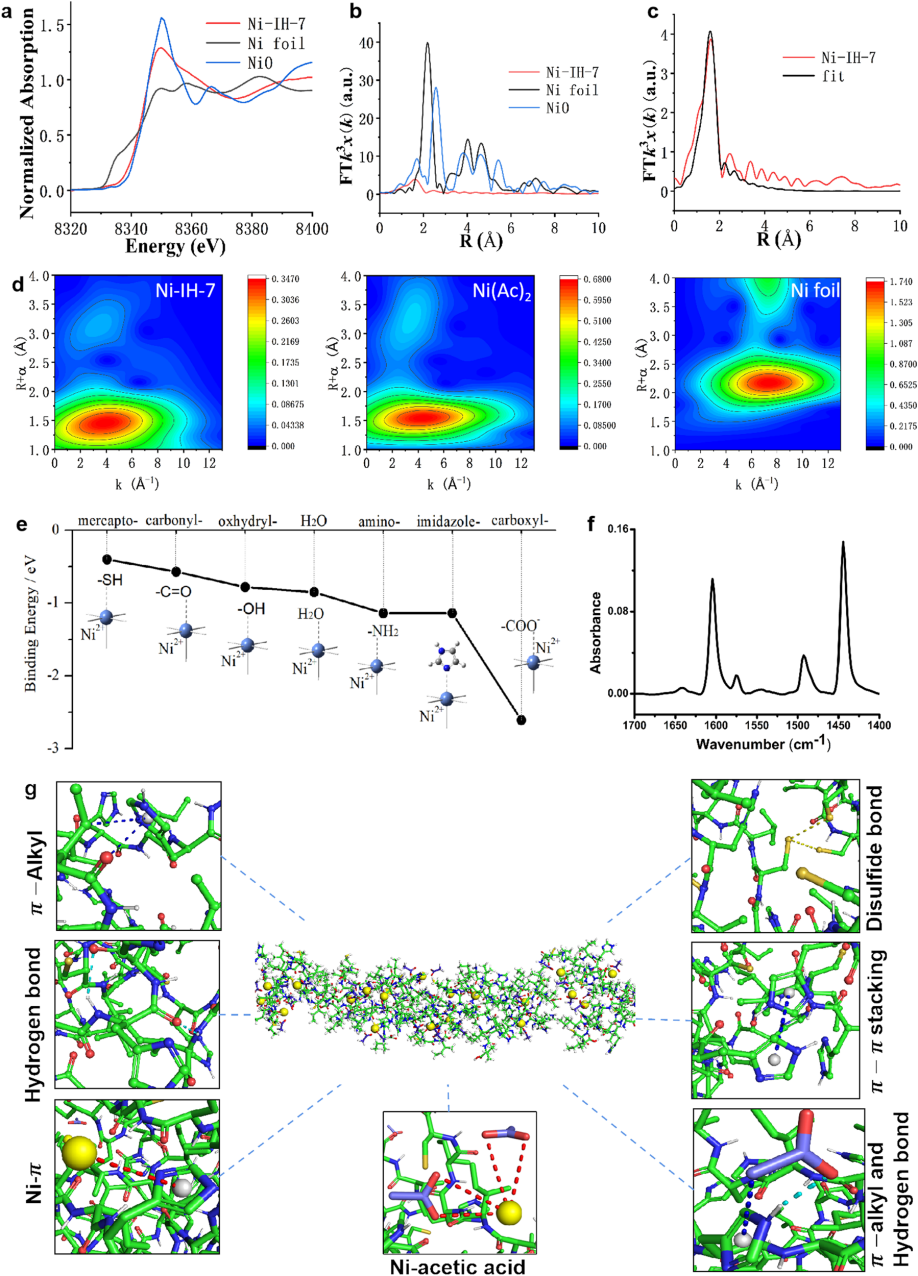
Ni coordination characterization in Ni-IH-7 nanotubes. **a**, **b** XANES and EXAFS spectra at the Ni K-edge. **c** EXAFS fitting curves of Ni-IH-7 in R space. **d** WT of Ni-IH-7, Ni(Ac)_2_, and Ni foil (from top to bottom). **e** Binding energy between Ni^2+^ and different functional groups in heptapeptide molecules. **f** FTIR spectrum of Ni-IH-7 nanotube after pyridine adsorption at 50 °C. **g** Schematic diagram of the predicted helical tubular structure and snapshots of possible atomic interactions in the Ni-IH-7 nanotube. Three times each experiment was repeated independently with similar results. Representative images are shown. Source data are provided as a Source Data file.

To understand the interactions between Ni^2+^ and heptapeptides in the tubular nanostructure, advanced characterizations were conducted. The local structure of the Ni-IH-7 nanotubes was further determined by X-ray absorption spectroscopy. Firstly, Ni K-edge X-ray absorption near-edge structure (XANES) demonstrated that the absorption edge of Ni-IH-7 was similar to that of NiO but different to that of Ni foil (Fig. 4a), indicating the presence of Ni–O or Ni–N coordination. In addition, the formation of π-π stacking was promoted in Ni-IH-7 (Supplementary Fig. 36 and Supplementary Table 5). Secondly, phase-uncorrected Fourier transformed extended X-ray absorption fine structure (EXAFS) characterization provided further evidence for the presence of Ni–O or Ni–N bonds in Ni-IH-7, as the EXAFS spectrum of Ni-IH-7 had a peak at 1.6 Å which is similar to the main peak of Ni–O at 1.7 Å in standard NiO, while Ni foil with Ni–Ni bond had a main peak at 2.2 Å (Fig. 4b). Lastly, quantitative EXAFS curve fitting analysis were performed to investigate the structural parameters of Ni-IH-7. The calculated mean Ni–O/N bond distance in Ni-IH-7 was 2.07 Å, which was different to that of Ni foil (2.48 Å). The best-fitting analysis demonstrated that the Ni–O/N coordination number in Ni-IH-7 nanotube was approximately 6.1 (Fig. 4c and Supplementary Table 6). The wavelet transform (WT) map demonstrated that the pattern of Ni-IH-7 was similar to Ni(Ac)_2_ rather than Ni foil, further proving that Ni–O/N bond, not Ni–Ni bond, has been formed in Ni-IH-7 (Fig. 4d). Since one Ni^2+^ only coordinates two CH_3_COO^−^, other coordination was possibly from Ni–N bond. In addition, the calculation of binding energy of Ni with different groups in the heptapeptide showed that amino, imidazole, and carboxyl groups but not mercapto, carbonyl, and oxhydryl groups could bind to Ni^2+^ with lower energy than H_2_O (Fig. 4e). Since one heptapeptide of IHIHICI had two His with imidazole groups, we speculated that Ni–N coordination with His should be present in Ni-IH-7 nanotubes, which might form catalytic site to mimic the active center of natural enzymes. These results demonstrated that Ni mainly coordinated with O from acetate and carboxyl group of heptapeptide and with N from His in the peptide. By pyridine adsorption, the ratio of B/L was calculated as 0.0755, indicating that more formation of Ni–N rather than Ni–O (Fig. 4f).

To understand the potential interactions, atomistic molecular dynamic simulations were performed based on the granulation model. As shown in Fig. 4g, there might be typical π–alkyl, π–π, S–S, and hydrogen bonds in the Ni-IH-7 molecules. Meanwhile, Ni–π, π–alkyl, metal coordination, and hydrogen bonds may be formed in the presence of Ni acetate (Supplementary Fig. 37). The increased number of hydrogen bonds per peptide was consistently shown during the first 100 ns (Supplementary Fig. 38), possibly due to the participation of CH_3_COO^−^. Thus, the interactions between peptides could be strengthened by the addition of Ni(Ac)_2_, thereby inducing the formation of nanotubes. Similar molecular dynamic simulation was conducted using the model of IHIHICI with NH_4_Ac (Supplementary Figs. 39 and 40). Although similar possibility and interactions in IHIHICI assembly, Ni(Ac)_2_ promoted IHIHICI assembly much faster than NH_4_Ac.

We also calculated the changes in the interaction forces (free energy) between free peptides and Ni-containing peptides during the stretching dynamics. The maximum force required to separate peptides in the presence of Ni(Ac)_2_ than that without Ni(Ac)_2_, indicating that Ni(Ac)_2_ is more likely to promote peptide assembly (Supplementary Fig. 41). Furthermore, the calculations on Gibbs free binding energy (ΔG_gibbs_) demonstrated that the assembly of Ni-IH-7 may involve the following processes: (1) Interactions between heptapeptide monomers (IH-7/IH-7), (2) Interactions between heptapeptide monomer and Ni-IH-7 oligomer formed from heptapeptide and Ni(Ac)_2_, (3) Interaction between single Ni(Ac)_2_ and two heptapeptide monomers (Supplementary Table 7). Altogether, the above characterizations and analyzes indicate that Ni(Ac)_2_ strengthens the molecular interactions in the Ni-IH-7 assembly process.

### Ni-IH-7 nanotubes perform phospholipase C-like catalysis

**Fig. 5:**
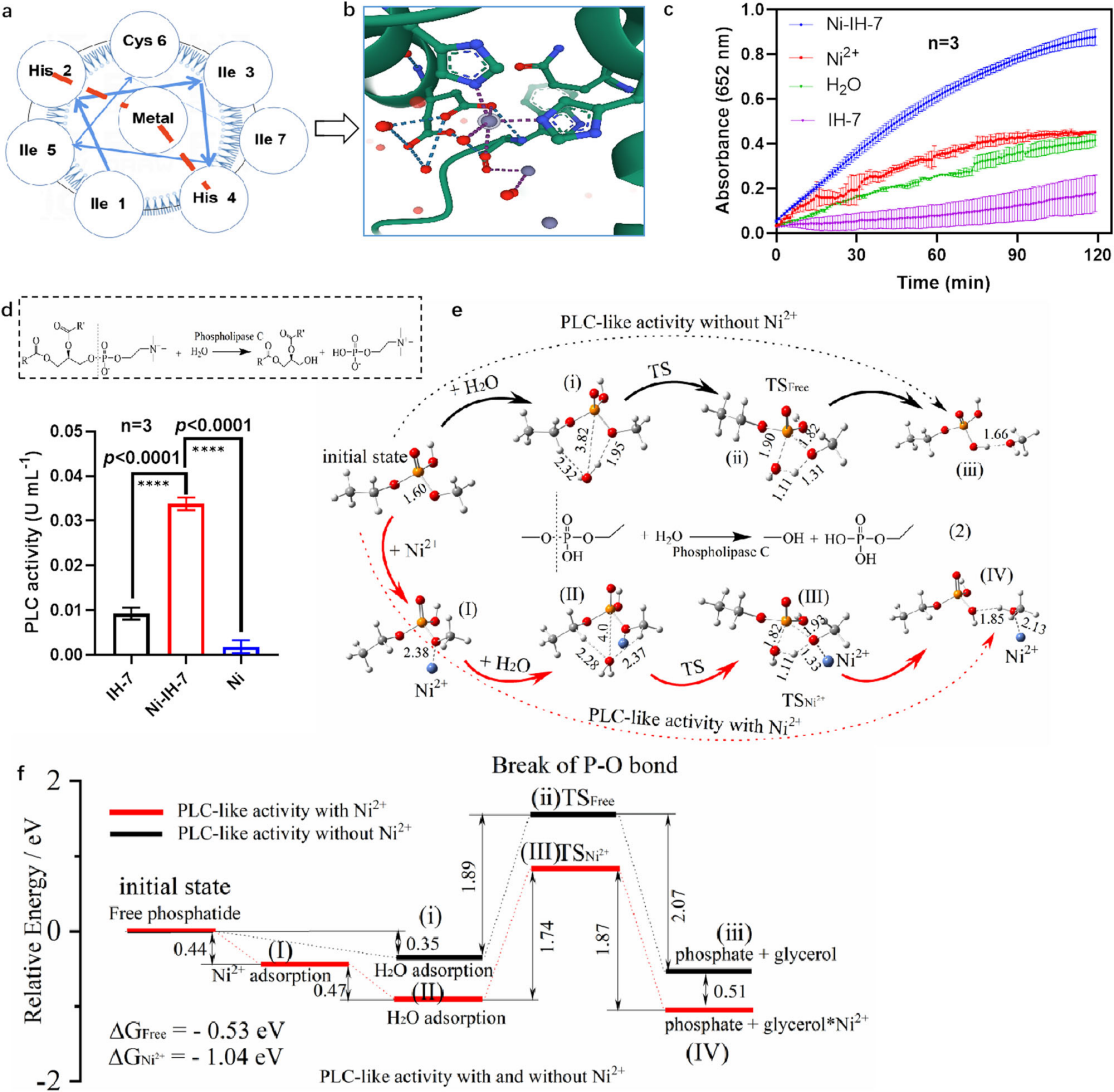
Enzyme-like activity of Ni-IH-7 nanotubes. **a**, **b** Schematic diagram of metal-His coordination mimicking active center in natural enzyme. **c** POD-like activity of Ni-IH-7 nanotubes. The significant difference was evaluated by a two-tailed unpaired t-test. *n* = 3 independent samples, bars represent means ± SD. **d** Hydrolytic reaction mediated by PLC. The significant difference was evaluated by a two-tailed unpaired *t*-test. *n* = 3 independent samples, bars represent means ± SD, ****p < 0.0001. **e** Proposed reaction pathways mediated by PLC-like catalysis of Ni-IH-7 nanotubes. **f** Gibbs free energy profile for PLC-like catalysis of Ni-IH-7 nanotubes. Three times each experiment was repeated independently with similar results. Representative images are shown. Source data are provided as a Source Data file.

The unique coordination of metal with His in Ni-IH-7 nanotubes provides potential active site to simulate active center of natural enzymes. In Ni-IH-7 nanotubes, Ni^2+^ coordinates with the N of the imidazole in His, which makes the nanotubes possible to mimic the activity of enzymes, such as oxidases, peroxidases, hydrolases owing the active center of metal cofactor and His coordination (Fig. 5a, b). By the activity screening, Ni-IH-7 showed a typical POD-like activity (Fig. 5c) and PLC-like activity (Fig. 5d) which involves cleavage of P-O bonds in phosphatide molecules to produce an inositol triphosphate molecule and a diacylglycerol molecule (Fig. 5d). In particular, the PLC-like activity showed a dependence on Ni, as IH-7 alone showed very low activity, indicating that Ni plays a cofactor role for the catalysis. To further evaluate the specific contribution of Ni to PLC-like activity, density function theory (DFT) was used to analyze the reaction pathway with or without Ni^2+^ (Fig. 5e, f). The calculations demonstrated that PLC-like activity without Ni^2+^ comprised two steps: H_2_O adsorption and hydrolysis with TSfree, with a corresponding energy barrier of 1.89 eV. In comparison, PLC-like activity with Ni^2+^ comprises three steps: Ni^2+^ adsorption, H_2_O adsorption, and hydrolysis with TSNi^2+^, with a corresponding energy barrier of 1.74 eV. Thus, compared to the pathway without Ni^2+^, Ni^2+^ adsorption reduced the energy barrier of the transition state by 0.15 eV. In addition, the change in Gibbs free energy for the reaction in the presence of Ni^2+^ was 0.51 eV lower than the reaction without Ni^2+^ (Fig. 5f). These calculations indicate that breaking of the phosphorus-oxygen bond in phosphatide molecules is primarily mediated by Ni as cofactor. Therefore, Ni-IH-7 nanotubes perform PLC-like activity owing to Ni coordination, which belongs to a type of nanozymes defined as nanomaterials with enzyme-like property.

### High stability of Ni-IH-7 nanotubes

**Fig. 6:**
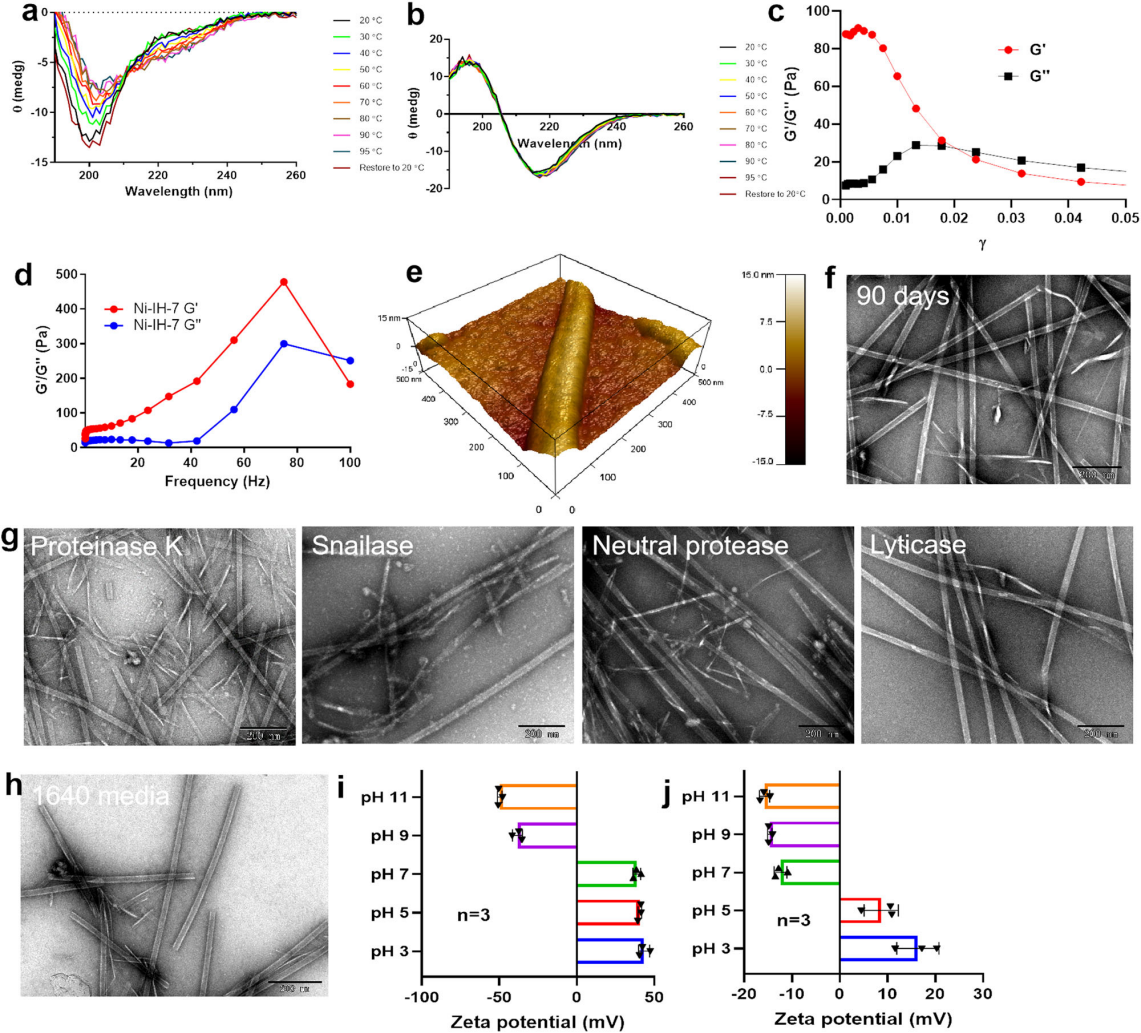
The stability assessment of Ni-IH-7 nanotubes. **a**, **b** Variable temperature CD of the heptapeptide (IH-7) and Ni-IH-7 nanotubes. **c**, **d** The fluid mechanics of the nanotubes assessed with rheological tests and modulus tests. **e** Surface charge of Ni-IH-7 nanotubes characterized by Kelvin probe force microscopy (KPFM). **f** Long-term storage of Ni-IH-7 nanotubes at room temperature. **g** Resistance to enzymatic degradation of Ni-IH-7 nanotubes. **h** Morphology of Ni-IH-7 nanotubes in 1640 culture media. **i**, **j** Zeta potential of Ni-IH-7 nanotubes in water and 1640 culture media under different pH. *n* = 3 independent samples, bars represent means ± SD. Three times each experiment was repeated independently with similar results. Representative images are shown. Source data are provided as a Source Data file.

Besides catalytic property, the physicochemical stability features of Ni-IH-7 nanotubes were further characterized. Circular dichroism at variable temperature showed that Ni-IH-7 nanotubes retained β-sheet conformation at the range of 20–95 °C, while the conformation of heptapeptide alone changed from random coil at low temperature to α-helix at high temperature (Fig. 6a, b), indicating that Ni-IH-7 nanotubes have superior thermal stability compared to the heptapeptide monomer. Furthermore, the fluid mechanics of the nanotubes was assessed with rheological tests. The amplitude sweep curve showed that the elastic response of the gel (Gʹ) exceeded the viscosity response (Gʹʹ) by a low Shear strain (γ < 1%), while the flow point was appeared as the loss factor (tanδ = Gʹʹ/Gʹ) reached more than 1 when the γ > 1%, indicating the transition from elasticity to viscosity (Fig. 6c). Rheological measurements of dynamic strain sweeps demonstrated that a trend from Gʹ > Gʹʹ to Gʹʹ > Gʹ occurred when the frequency increased (Fig. 6d), indicating that the nanotubes in water formed entangled liquid paste rather than hydrogel. Kelvin probe force microscopy (KPFM) demonstrated that the Ni-IH-7 nanotubes were positively charged with 443.52 mV (Fig. 6e and Supplementary Fig. 42). The as prepared Ni-IH-7 nanotubes remained good morphology after storing for 90 days at room temperature (Fig. 6f). In addition, the nanotubes showed considerable resistance to enzymatic degradation by proteinase K, snailase, neutral protease and lyticase (Fig. 6g). These features ensured the Ni-IH-7 nanotubes with good stability in culture media (Fig. 6h). Zeta potential assay showed that the Ni-IH-7 nanotubes were positively charged in the water with pH ≤ 7, while the nanotubes were negatively charged in 1640 culture media with pH at 7 (Fig. 6i, j), indicating a charge inversion under different environment. Such charge property indicates that Ni-IH-7 nanotubes are suitable to work under acidic environment that keeps them positive charge to bind microbes whose surface are negative charged (Supplementary Fig. 43). These results demonstrate that the Ni-IH-7 nanotubes possess high thermal stability and resistance to enzymatic degradation. The positive charged feature may facilitate their interaction with the surface of microbes.

### Ni-IH-7 Nanotubes integrate AMP and AME-like antifungal potency against *C. albicans*

**Fig. 7:**
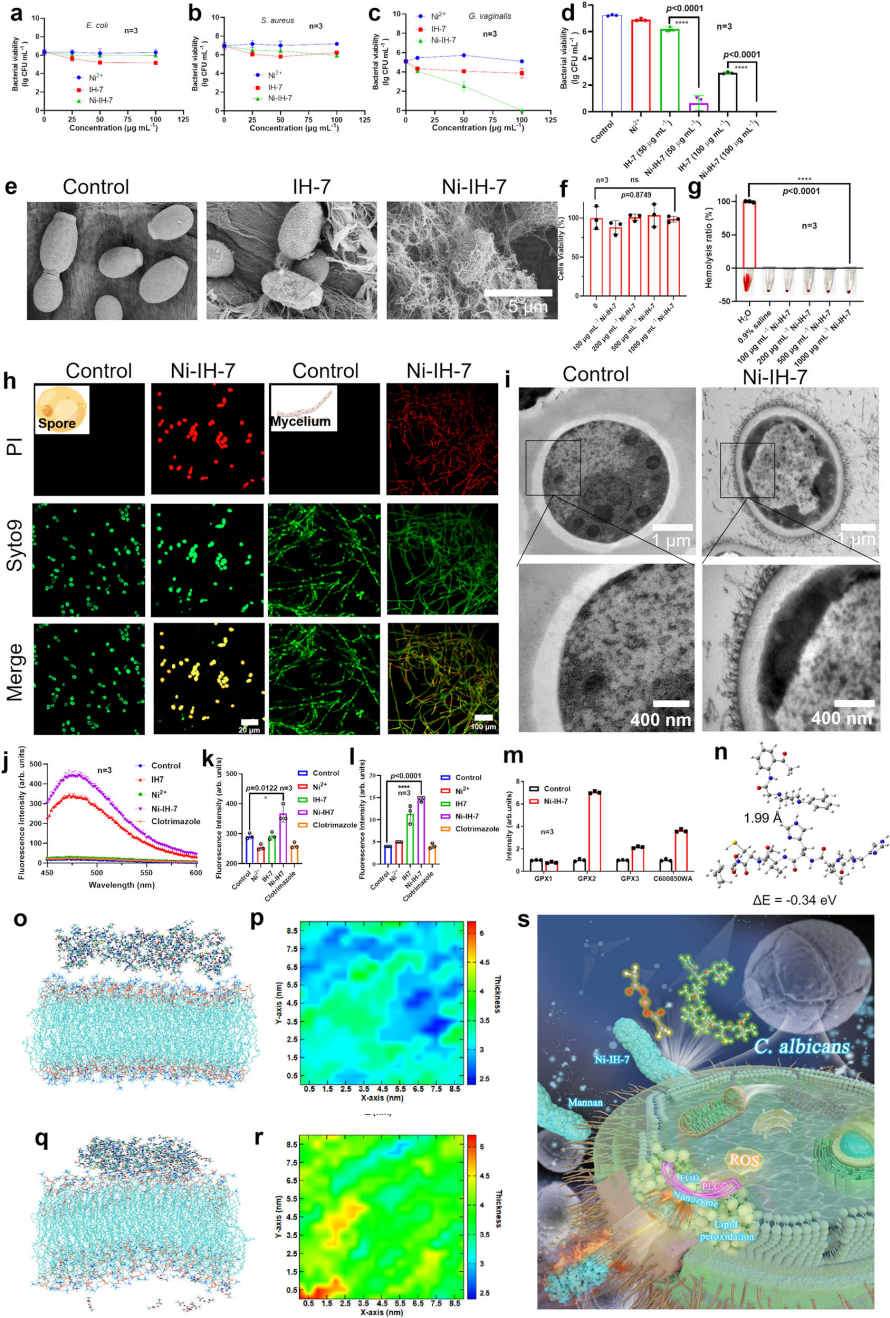
Antimicrobial activity and MoA of Ni-IH-7 nanotubes against *C. albicans.* **a**–**c** Antimicrobial activity of Ni-IH-7 nanotubes toward *E. coli* (Gram-negative species), *S. aureus* (Gram-positive species) and *G. vaginalis* (Gram-variable species), respectively. *n* = 3 independent samples, bars represent means ± SD. **d** Antifungal activity of Ni-IH-7 nanotubes toward *C. albicans*. *n* = 3. The significant difference was evaluated by a two-tailed unpaired *t*-test. *n* = 3 independent samples, bars represent means ± SD, ****p < 0.0001. **e** SEM characterization of *C. albicans* treated with Ni-IH-7 nanotubes. **f** Cytotoxicity of Ni-IH-7 nanotubes toward VK2 cells (from human vaginal mucosal tissue). The significant difference was evaluated by a two-tailed unpaired *t*-test. *n* = 3 independent samples, bars represent means ± SD. ns means no significant difference. **g** Hemolysis assessment of Ni-IH-7 nanotubes toward red blood cells from mice. The significant difference was evaluated by a two-tailed unpaired *t*-test. *n* = 3 independent samples, bars represent means ± SD, ****p < 0.0001. **h**, Confocal microscopy of planktonic yeast and mycelium biofilm of *C. albicans* treated by Ni-IH-7 nanotubes using live (Syto9)/dead (PI) staining. **i** SEM and TEM characterization of *C. albicans* treated with Ni-IH-7 nanotubes. **j**, **k** Cell membrane permeability and depolarization verified by ANS and diSC3(5) probes. The significant difference was evaluated by a two-tailed unpaired *t*-test. *n* = 3 independent samples, bars represent means ± SD, *p < 0.05. **l** Lipid peroxidation in *C. albicans* verified by BODIPY probe. The significant difference was evaluated by a two-tailed unpaired *t*-test. *n* = 3 independent samples, bars represent means ± SD, ****p < 0.0001. **m** qPCR validation for transcriptomic data of glutathione peroxidase (GPX) expression. *n* = 3 independent samples, bars represent means ± SD. **n** DFT calculation for potential interactions between Ni-IH-7 and mannan. **o** MD simulations of initial state of the interactions between Ni-IH-7 nanotube and POPC membrane. **p** Spatial distribution projection map of POPC membranes corresponding to (**o**). **q** MD simulation of final state of the interactions between Ni-IH-7 nanotubes and POPC membrane. **r** Spatial distribution projection map of POPC membrane corresponding to (**q**). **s** A schematic to summarize multi-antifungal actions of Ni-IH-7 nanotubes as an integrated nanozyme. Three times each experiment was repeated independently with similar results. Representative images are shown. Source data are provided as a Source Data file.

We next evaluated the antimicrobial properties of the assembled Ni-IH-7 in view of the unique nanotube structure, POD-like activity, PLC-like activity and high stability that integrate mechanism of action of AMPs and AMEs. A screening on antimicrobial activity toward different species [Fungus *C. albicans*, Gram-variable *Gardnerella vaginalis* (*G. vaginalis*), Gram-positive *Staphylococcus aureus* (*S. aureus*) and Gram-negative *Escherichia coli* (*E. coli*)]. was conducted. As shown in Fig. 7a–c, Compared to Ni^2+^ or IH-7 peptide, NI-IH-7 nanotubes showed no antibacterial effects on *E. coli* or *S. aureus*, while they partially killed *G. vaginalis* (Supplementary Figs. 44 and 45). In contrast, Ni-IH-7 exhibited remarkable antifungal activity toward *C. albicans* (Fig. 7d). In particular, Ni-IH-7 nanotubes showed higher antimicrobial activity than IH-7. Under in vitro aqueous condition, Ni-IH-7 nanotubes (at 50 µg mL^−1^, equal to 0.058 mM) reduced the viability of *C. albicans* about 6.5 lg [from 7.23 lg (CFU mL^−1^) to 0.65 lg (CFU mL^−1^)], while IH-7 peptide only reduced it just 1 lg [from 7.23 lg (CFU mL^−1^) to 6.21 lg (CFU mL^−1^)] (Fig. 7d), indicating that Ni-IH-7 nanotubes perform remarkably higher antifungal efficiency than IH-7 peptide. Similar antifungal potency and certain antibacterial effect were also achieved on three drug-resistant strains or clinical isolates of *C. albicans* (Supplementary Fig. 46 and Supplementary Table 8) and two clinical samples of resistant *G. vaginalis* (Supplementary Fig. 47), respectively. Correspondingly, SEM characterization showed that abundant Ni-IH-7 nanotubes entangled on *C. albican* to form a Cocoon-like structure, while no much binding on *G. vaginalis*, *E. coli* or *S. aureus* (Fig. 7e and Supplementary Fig. 48). These data demonstrated that the de novo designed heptapeptide has certain antimicrobial activity and the assembly into nanotubes of Ni-IH-7 significantly improved the antifungal performance. In addition, Ni-IH-7 nanotubes showed negligible cytotoxicity to VK2 cells (vaginal epithelial tissue) (Fig. 7f). Hemolysis assay showed that Ni-IH-7 nanotubes did not damage red blood cells even at high concentration up to 1 mg mL^−1^ (Fig. 7g). These results demonstrated that Ni-IH-7 exerted selective antimicrobial activity and high biocompatibility.

To further understand antifungal mechanism of action of Ni-IH-7 nanotubes, Live/dead assay with Syto9/PI staining characterized by confocal microscopy was conducted for both planktonic and biofilm-embedded *C. albicans*. As shown Fig. 7h, after Ni-IH-7 treatment, dead *C. albicans* whether in planktonic yeast state or in mycelium stage in biofilm were significantly enhanced. Further high-resolution TEM characterizations demonstrated that large amount of Ni-IH-7 nanotubes wrapped on the surface of *C. albicans* and bound to outer mannan layer *C. albicans* and inserted into the cell wall. Treatment with Ni-IH-7 resulted in the disruption of the cell wall integrity, enlargement of nucleus area and disappearance of suborganelles such as mitochondria (Fig. 7i). Importantly, such process was accompanied with enhanced membrane permeability and depolarization determined by 1-Anilino-8-naphthalene sulfonate (ANS) and 3,3’-Dipropylthiadicarbocyanine Iodide (diSC3(5)) probes (Fig. 7j, k). In addition, typical lipid peroxidation occurred with increased GSSG/GSH ratio (Fig. 7l and Supplementary Fig. 49), demonstrating that the cell membrane of *C. albicans* was disrupted and suffered heavily oxidative stress. The transcriptome analysis demonstrated that the expression levels of genes involved in regulation of the cell wall, cell membrane, and lipid peroxidation (the transcription levels of GPX were upgraded) were dramatically altered compared to untreated *C. albicans* (Supplementary Fig. 50). The qPCR validated the reliability of transcriptomic data, in particular for increased GPX2 (Fig. 7m, Supplementary Figs. 51 and 52). Metabolomic analyzes demonstrated that levels of glycerophospholipids, glutathione, and mannan were altered in *C. albicans* following treatment with Ni-IH-7 nanotubes (Supplementary Figs. 53 and 54). These results demonstrated that Ni-IH-7 killed *C. albicans* not only through cell wall and membrane disruption which is similar to the action of AMPs, but also induced membrane lipid peroxidation and ferroptotic responses.

To further understand the mechanism of action, density function theory (DFT) and molecular dynamics (MD) simulations were conducted to investigate the interactions between Ni-IH-7 nanotubes and *C. albicans*. As shown in Fig. 7n, DFT calculation showed that the interaction energy between the heptapeptide and mannan was at least −0.34 eV which was further converted to the dissociation constant (K_*D*_) at 1.76 × 10^−6^ M, indicating that Ni-IH-7 nanotubes have high binding affinity to mannan. To further prove this, we tested the antifungal efficiency of Ni-IH-7 nanotubes toward fungi with different mannan content. Firstly, we found that the antifungal ability of Ni-IH-7 nanotubes disappeared in the presence of β-mannanase which can degrade mannan on the wall of *C. albicans* (Supplementary Fig. 55a–c). Secondly, we found *C. auris* with higher mannan in the cell wall^37^ could be more easily killed by Ni-IH-7 than *C. albicans* (Supplementary Fig. 55d). In addition, we found that *G. vaginalis* also contained certain level of mannan, which can be used to explain why Ni-IH-7 can kill them but failed to *E. coli* or *S. aureus* (Supplementary Table 9). These results provide a direct evidence that the mannan is a critical target for Ni-IH-7 nanotubes to perform antifungal action.

Furthermore, using 1-Palmitoyl-2-oleoyl-sn-glycero-3-PC (POPC) as a model membrane, MD simulation showed that compared to the initial state, after a 50 ns simulation, an obvious interaction between Ni-IH-7 nanotubes and POPC molecules was observed, including significant thickening of the POPC membrane and structural collapse under the influence of Ni-IH-7 nanotubes (Fig. 7o–r). The entire POPC membrane had uniform thickness, although the overall thickness was small (Fig. 7o, p). However, the embedding of Ni-IH-7 nanotubes in the POPC membrane led to significantly increased thickness of the entire POPC membrane with an uneven thickness distribution, indicating that the binding of Ni-IH-7 nanotubes disrupts the structure of the POPC membrane (Fig. 7q, r). The above theoretical analyzes demonstrated that Ni-IH-7 nanotubes are able to readily bind with mannan in the outer wall and disrupt membrane integrity of *C. albicans*. In particular, the destruction of the cell membrane and lipid peroxidation may be correlated to the coordinated Ni in Ni-IH-7 nanotubes which endowed PLC-like activity to degrade membrane and redox activity to induce oxidative stress, thus demonstrating AMEs-like antimicrobial action when Ni-IH-7 nanotubes acting as a nanozyme (Fig. 7s).

To investigate the potential application, the vaginal secretions of vaginitis patients were used to verify the antifungal ability of Ni-IH-7 nanotubes. As expected, the Ni-IH-7 nanotubes showed significant antifungal effects by diluting vaginal secretion with 0.9% NaCl (Supplementary Fig. 56a and b). It should be noted that the Ni-IH-7 nanotubes had been stored at room temperature for 100 days prior to use. Further, we were also able to fix Ni-IH-7 nanozymes to medical pads by soaking the pads in a nanotube solution for 10 min followed by drying in air for one week (Supplementary Figs. 56c and  57). Impressively, 95% *C. albicans* were killed within 10 min on such pad with Ni-IH-7 nanotubes. (Supplementary Fig. 56d).

## Discussion

In this study, we have developed an artificial peptide-assembled nanozyme using de novo design assisted by AlphaFold2 and precise morphology modulation with acetate salts to achieve antimicrobial actions of AMPs and AMEs in one system. Different to natural AMPs and AMEs with large amino acid sequences, the peptide has been comprised of just three amino acids: His, Cys and Ile, in which the first two are often key residues in the active center of AMPs or AMEs and the last determines folding structure. AlphaFold2 and MD predict that the shortest sequence of heptapeptide is able to form β-sheet conformation and high-resolution characterization with AFM and Cryo-EM confirms that the heptapeptide assembled into β-helical nanotubes. Importantly, Ni coordination endowed the nanotubes as a nanozyme with PLC-like activity and peroxidase-like activity. Such peptide-based nanozyme shows high stability to thermal stimuli and enzymatic degradation, and have performed antifungal activity integrating AMPs mode of targeting to wall disruption and AMEs mode of catalytically inducing microbial death. We notice ferroptotic death has been induced when *C. albicans* exposed to Ni-IH-7 nanozyme, which may be a unique antifungal mechanism depending on Ni coordination^38^. The surface charge property and specific antimicrobial efficiency toward *C. albicans* and *G. vaginalis* may enable single Ni-IH-7 nanozyme for treatment and prevention of bacterial vaginosis which is caused by the prevalence of the above two pathogens under vaginal environment with acidic pH^39,40^. To expand practical use, further rational design may be required to optimize physicochemical property including surface charge and morphology and enhance enzyme-like activity to improve antimicrobial potency and enzyme. Taken together, our study provides a proof-of-concept to design an artificial system that can integrate antimicrobial features of AMPs and AMEs but overcome their limitation of poor stability, which will help to develop superior antimicrobial candidates against the challenge of antimicrobial resistance.

## Methods

### Ethical regulations

All research complied with all relevant ethical regulations. All animal studies were performed following the protocols approved by the Animal Ethics Committee of Zhengzhou University. The use of the samples of vaginal secretions from patients with fungal infection was approved by the Ethics Committee of Wuxi Xishan People’s Hospital (approval number: LLS2020ky036 27/07/2020) and obtained informed consent from all patients.

### Materials

All peptides (purity > 95%) were purchased from GL Biochem (Shanghai, China). Glutaraldehyde (2.5%), Amphotericin B and the Micro Total Mercapto Assay Kit were purchased from Beijing Solarbio Science & Technology Co., Ltd. (China). Various salt compounds, Metronidazole and 5-Flucytosine were purchased from Shanghai Macklin Biochemical Co., Ltd. (China). Itraconazole was purchased from Jinclone (Beijing) Biotechnology Co., Ltd. (Beijing, China). Fluconazole was purchased from Shanghai Yuanye Bio-Technology Co., Ltd. (China). Propidium iodide (PI), GSH and GSSG Assay Kit were purchased from Beyotime Biotechnology (Shanghai, China). PLC activity Assay Kit and Syto9 green fluorescent nucleic acid stain was purchased from Thermo Fisher Scientific (USA). 8-anilinonapthalene-1-sulfonic acid (ANS) and 3,3ʹ-dipropylthiadicarbocyanine iodide (diSC3(5)) were purchased from Shanghai Bepharm Science&Technology Co., Ltd (Shanghai, China). BODIPY 581/591 C11 probe was purchased from Sigma-Aldrich Co., Ltd. (USA).

### AlphaFold2 prediction

Due to the enhanced capability of AlphaFold2 compared to AlphaFold, it includes the functionality to predict multimeric structures and their interactions. We set the maximum limit, provided by the open-source online platform, for the total number of amino acids to 60× (1× = 7 amino acids).The number of multimers for all peptides was standardized to 60×, and num_recycles was configured as 24 or 48. The template_mode was set to none. Msa_mode, pair_mode, and model_type were set to options named MMseqs2, unpaired + paired, and auto, respectively. For each prediction, five different models were provided. The model with the highest per-residue pLDDT score, specifically rank_001 (indicating high confidence), was selected for comparison with the experimentally observed structure.

### Sample preparation

Powders of each of the peptides were dissolved in 1.5 mM Ni(Ac)_2_ solution to a certain concentration range (e.g. 0.0–1.5 mM) and sonicated for a period of time, and were then allowed to cool gradually to 25 °C overnight. Aged samples could be left at room temperature for more than 90 days.

### MD simulations for Ni-IH-7 assembly

GCMD simulations have been performed using the general purpose Martini force field^41,42^. All systems were generated randomly placing 150 peptides and Ni(Ac)_2_ into a cubic box of ~ 10 nm, the coarse-grained water box was used to solvate the initial system before thousands of steps of energy minimization. The leap-frog algorithm with a 20 fs time step was used with neighbor list updated every 20 steps using a cutoff 1.1 nm. The coulomb potential is addressed by using the reaction-field method and the LJ potential is dealt with by using Potential-shift-verlet, and the standard relative dielectric constant of Coulomb potential of 15 was used. The pressure and temperature were controlled at 1.0 atm and 298.0 K by the Berendsen method. Coarse-grained molecular dynamics simulations have been performed in the GROMACS^43^ (version 2020.6) simulation package for 2 µs. As for the calculations of AP, 20 different sequences of peptides were first randomly placed in a cubic box of around 10 nm and coarse-grained water molecules were used to solvate the whole system. The Ni(Ac)_2_ were placed through random replacement of water molecules, and then they were performed similar steps as above. AP values (SASA_initial_/SASA_final_) were calculated as the ratio of solvent accessible surface areas (SASAs) at the start (SASA_initial_) and finish (SASA_final_) of GCMD^35^.

1 $${{{{{\rm{AP}}}}}}={{{{{{\rm{SASA}}}}}}}_{{initial}}/{{{{{{\rm{SASA}}}}}}}_{{final}}$$

Molecular dynamics were also performed in the GROMACS (version 2020.6) simulation package, and the General Amber force field (GAFF2) was used for the Polypeptide complex. The parameters for Ni used the Universal force field (UFF)^44^, which covers the whole periodic table. The TIP3P water model was used to describe the water molecules, and the cell membranes were chosen POPC (1-palmitoyl-2-oleoyl-sn-glycero-3-phosphocholine), there are 128 lipid molecules for each membrane model, the CHARMM36 force field was used for the POPC. Molecular dynamics simulations under the iso-thermo and iso-baric ensemble after thousands of steps of energy minimization were implemented using the Berendsen method under 1 atm along the norm of the surface at 298 K for an equilibration period of 5 ns. Subsequent 50 ns production runs were carried out for each system using the Nose–Hoover and Parrinello–Rahman coupling methods. A cutoff scheme was implemented at 1.2 nm for the non-bonded interactions, and the Particle Mesh Ewald method^45^ with a fourierspacing of 0.1 nm was applied for the long-range electrostatic interactions. All covalent bonds with were constraint using the LINCS algorithm^46^.

### MD simulations for Ni-IH-7

The protein structure was built using Alphafold2 to construct the three-dimensional structure of the peptide (sequence IHIHICI). The three-dimensional structure was further optimized using UCSF Chimera^47^, and the atomic charges of the protein were calculated using AMBER14SB. The pKa values of amino acids were calculated and assigned under neutral conditions (pH = 7) using the online tool H + + 3^48^. Subsequently, this study utilized PackMOL (18.002)^49^ to construct the self-assembled initial model, which included 20 peptide chains randomly placed in the system. The PackMOL tolerance was set to 2.0 Å.

#### MD simulations

This study employed the open-source software package Gromacs 5.1.5^50^ for molecular dynamics simulation. The simulation system was placed in a closed environment with a temperature of 289.15 K (room temperature), pH of 7, and pressure of 1 bar. Periodic boundary conditions were set with the protein at the center, and the minimum distance from the protein edge to the box edge was set to 10 Å. The pdb2gmx tool was used to convert the topology file of the receptor structure into a GROMACS-readable file, with the AMBEff14SB force field and TIP3P water molecules simulating the water environment^51,52^. The initial system included 20 peptide chains and 39,659 TIP3P water molecules, totaling 121,637 atoms. After constructing the initial system, a steepest descent algorithm was applied to minimize the energy of all atoms. Subsequently, constant number of particles, volume, and temperature (NVT) equilibrium simulation for 1000 ps and constant number of particles, pressure, and temperature (NPT) equilibrium simulation for 1000 ps were performed. After NVT and NPT equilibration, both wild-type and mutant systems underwent a 100 ns dynamic production simulation, with a simulation interval of 2 fs. Covalent bond lengths were constrained using a linear constraint solver algorithm, and long-range electrostatic interactions were treated with the Particle Mesh Ewald method^53^. For the analysis of the rdg (reduced density gradient), single-point energy calculations were first performed on the aggregated peptides under the conditions of the B3LYP functional and the 6-31+g(d,p) basis set. The keyword “output=wfn” was included in the calculations. Subsequently, Multiwfn3.8 was employed for Hirshfeld partition of molecular density (IGMH) analysis to investigate the distribution of van der Waals weak interactions during the aggregation of peptides.

### The stretching dynamics simulations for IH-7 and Ni-IH-7

The initial model predicted by Alphafold2 was used as the initial structure for the water phase simulation, where no disulfide bond structure was formed between peptides. The optimization of the peptide dimer with disulfide bond structure as the initial structure was performed using UCSF Chimera under Ni(Ac)_2_ conditions. Subsequently, PackMOL was used to construct the initial model of stretching dynamics simulation under Ni(Ac)_2_ conditions, including 20 peptide chains and 40 Ni(Ac)_2_ molecules, randomly placed with a PackMOL tolerance set to 2.0 Å.

#### Dynamics Simulation

System 1 represented the stretching process of peptides in an aqueous environment, while System 2 represented the stretching process of peptide dimers in Ni(Ac)_2_ environment. The simulation systems were set in a closed environment with a temperature of 289.15 K (room temperature), pH of 7, and pressure of 1 bar. Periodic boundary conditions were set with the protein at the center, and the minimum distance from the protein edge to the box edge was set to 10 Å. The pdb2gmx tool was used to convert the topology file of the receptor structure into a GROMACS-readable file, with the AMBEff14SB force field and TIP3P water molecules simulating the water environment. System 1 included 20 peptide chains and 4,978 TIP3P water molecules, totaling 12,597 atoms. System 2 included 20 peptide chains, 40 Ni(Ac)_2_ molecules, and 12,597 TIP3P water molecules, totaling 38,175 atoms. After constructing the initial systems, a steepest descent algorithm was applied to minimize the energy of all atoms. Subsequently, NVT equilibrium simulation for 1000 ps and NPT equilibrium simulation for 1000 ps were performed. After NVT and NPT equilibration, both systems underwent a 20 ns dynamic production simulation, with a simulation interval of 2 fs. External forces were applied during the simulation for stretching, using a Gaussian potential with a Height of 0.03 kcal/mol, stretching interval time of 0.09 ps, and Width interval of 0.05. Covalent bond lengths were constrained using a linear constraint solver algorithm, and long-range electrostatic interactions were treated with the Particle Mesh Ewald method.

### Gibbs free binding energy (ΔG_gibbs_)

The systems were subjected to 100 ps energy optimization using the Gromacs 5.1.5 software. The optimized structures were then used with the MMPBSA.py program from AmberTools to calculate the Gibbs free energy ΔGgibbs of the systems based on atomic properties. The calculation involved covalent bond energy (COV), solvent free energy based on molecular mechanics/Poisson-Boltzmann (Generalized Born) model (SGB), and non-covalent interaction energies such as van der Waals forces (Lennard-Jones potential, LJ), hydrophobic effects (Lipophilic, Lipo), and electrostatic interactions (Electrostatic, EL). Additionally, hydrogen bond interactions (Hydrogen bond, HBOND) and π-π stacking interactions (PACKING) were calculated. The entropy contribution to the free energy was also computed based on the molecular mechanics/Poisson-Boltzmann (Generalized Born) model.

2 $$\Delta {{{{{{\rm{G}}}}}}}_{{{{{{\rm{gibbs}}}}}}}=\Delta {{{{{\rm{H}}}}}}-{{{{{\rm{T}}}}}}\Delta {{{{{\rm{S}}}}}} \, \approx \, \Delta {{{{{{\rm{G}}}}}}}_{{{{{{\rm{covalent}}}}}}}+\Delta {{{{{{\rm{G}}}}}}}_{{{{{{\rm{nonbonded\; energy}}}}}}}+\Delta {{{{{{\rm{G}}}}}}}_{{{{{{\rm{Other}}}}}}}-{{{{{\rm{T}}}}}}\Delta {{{{{\rm{S}}}}}}$$

### Docking calculation for the interaction of IH-7 and Ni(Ac)_2_

AutoDock 4.2 software was employed for molecular docking experiments, setting the box size as a cube with a side length of 22.5 Å and a spacing step of 0.375. The maximum limit for conformational search was set to 10000, and a genetic algorithm was used for conformation sampling and scoring. The optimal conformations were selected based on the docking scores. Ni(Ac)_2_ molecules were generated in three dimensions using RDKit (2024.03.3) and assigned AM1-BCC partial charges using UCSF Chimera (1.15).

### Electron microscopy for peptide samples

TEM images were recorded using a JEM1200EX (JEOL) (Japan) operating at 100 kV dropped on a double copper net. Typically, 1.5 mM samples are diluted 10-fold and stained with 3% uranyl acetate for viewing. For AFM, liquid Ni-IH-7 (1 mL) was dropped on a mica sheet and observed by Bruker Dension Icon (Germany). The AMFM data were fitted using the Analyze panel module in Igor Pro software to obtain a modulus curve, yielding the average Young’s modulus. Simultaneously, we utilized the Hertz model force curve fitting results to validate the reliability of the Young’s modulus. Cryo-EM and two-dimensional classification data of Ni-IH-7 were collected by Talos Arctica 200 kV FEG (USA). AMFM was tested using Cypher S (USA) in the presence of AC160TS-R3 (probe). Samples were diluted 50 times and dried on mica flakes. KPFM was tested using Cypher S (USA) and obtain the Contact potential difference. The standard highly oriented pyrolytic graphite was 675 mV. HAADF-STEM was obtained by JEM ARM 200 F (accelerating voltage: 200 kV) (JEOL, Japan).

### Characterizations based on spectroscopy, FTIR, STM, PXRD, X-ray photoelectron spectroscopy (XPS), 2D NMR, zeta potential, CMC

The Ultraviolet-visible (UV-VIS) spectroscopy of samples were collected by a Shimadzu UV-3600. For FTIR, 3 mg of powdered sample and 50 mg of potassium bromide (KBr) were mixed for tablet compression analysis with transmittance at 400–4000 cm^−1^, collected using an iS10 FTIR spectrometer (USA). Measurements were performed by averaging 32 scans at 4-cm^−1^ resolution (signal-to-noise ratio (S/N) = 50,000). The single-molecule STM imaging experiments were performed under ambient conditions by using a Nanoscope IIIa SPM system (Bruker, USA). 10 μL of the peptide solution was incubated on the surface of freshly cleaved highly oriented pyrolytic graphite (HOPG) at room temperature for 20 min. Then the excess peptide solution was removed from the substrate surface. The STM tips were mechanically made by Ir/Pt wires (20/80). Different STM tips and samples were used in these imaging experiments to ensure reproducibility. The samples were characterized by PXRD using a D8 ADVANCE (Germany). Measurements were performed at 1.79026 Å of the cobalt target wavelength, 40-kV tube voltage, and 40-mA tube current. Zeta potentials of samples were determined using a Zetasizer Nano ZS90 (England) via three tests in a 1-mL sample cell. All CD datum were collected using Chirascan Plus (England) and analyzed at 190-260 nm. Time-per-point, step size and bandwidth were set as 0.5 s, 1 nm and 1 nm. The temperature was set as 25 °C. The datum of variable temperature CD were collected at 20, 30, 40, 50, 60, 70, 80, 90, 95 °C, respectively. Physical adsorption tests were completed as N_2_ and Ar atmosphere using ASAP 2460 (USA). Samples were degassed at room temperature for 24 h in advance. As for XPS, the Thermo Escalab 250XI from the United States was employed with the following parameters: monochromatic Al Kα X-ray source (hv = 1486.6 eV) with a power of 150 W, a spot size of 650 µm, an accelerating voltage of 14.8 kV, a current of 1.6 A, and charge correction using contaminated carbon C*1s* at 284.8 eV. Pyridine infrared (IR) were measured at 1400–1700 cm^−1^ using Thermo Scientific Nicolet 380 (USA). The temperature was set as 50 °C. As for 2D NMR, samples were dissolved in the buffer containing 1.5 mM IH-7, Ni-IH-7. All the 1H NMR experiments were performed on the 800 MHz spectrometer (AVANCE III HD) with a cryoprobe at 25 °C. The standard pulse sequence zgesgp was used for water suppression and data acquisition. The relaxation delay time and scan number were set to be 1.5 s and 16. The fluorescence signals (*I*_374nm_, *I*_394nm_) were collected for detecting CMC using the pyrene probe under excitation at 330 nm across various concentrations of peptides.

### XAS and Wavelet Transform analysis

The X-ray absorption spectra(XAS) including X-ray absorption near-edge structure (XANES)and extended X-ray absorption fine structure (EXAFS)of the samples at Ru K-edge (They were collected at the Singapore Synchrotron Light Source (SSLS) center, where a pair of channel-cut Si (111) crystals was used in the monochromator, The Ni K-edge XANES data were recorded in a transmission mode. The storage ring was working at the energy of 2.5 GeV with an average electron current of below 200 mA. The acquired EXAFS data were extracted and processed according to the standard procedures using te ATHENA module implemented in the FEFIT software packages. The k3-weighted Fourier transform (FT) of x(k) in R space was obtained over the range of 0–14.0 Å^−1^ by applying a Besse window function. For Wavelet Transform analysis, the χ(k) exported from Athena was imported into the Hama Fortran code. The parameters were listed as follow: R range, 1–4 Å, k range, 0–12 Å^−-1^ for standards and 0–9 Å^−1^ for Ni sample; k weight, 2; and Morlet function with κ = 10, σ = 1 was used as the mother wavelet to provide the overall distribution.

### POD-like and PLC-like activity of nanozyme

To detect POD-like activity, 140 µL of sodium acetate buffer (pH 4.5, 0.2 M), 20 µL of 10 mM TMB, 20 µL of 1.5 mM Ni-IH-7 or 1.5 mM 4H_2_O·Ni(Ac)_2_, and 20 µL of H_2_O_2_ (50 mM) were added in order and reacted for 2 h at 37 °C. Absorbance at 652 nm was monitored using a microplate reader. PLC activity Assay Kit was used to detect PLC-like activity.

### DFT calculations

All geometries, including local minima and transition states (TS), were fully optimized using the B3LYP density functional in conjunction with the 6-31 G(d,p) basis set. The harmonic frequency analysis was performed for each structure to identify whether the stationary point is a local minimum or a transition state and to obtain the Gibbs free energy. All the calculations were carried out using the Gaussian 09 package.

### Antifungal activity of Ni-IH-7 and its permeability and depolarization of fungal membranes

*albicans* (ATCC10231, ATCC90028, ATCC90029) were employed in our experiments. Their antifungal names and the resistance degree were included in Supplementary Table 7. The Multidrug-resistant strain was provided by Xishan People’s Hospital of Wuxi City (including *Candida auris* (*C. auris*)). First, *C. albicans* stored at -80°C was thawed and inoculated in Sabouraud’s liquid medium with shaking overnight. The volume of the culture medium was 10 mL, and the incubation condition was 37°C in an air incubator. On the third day, *C. albicans* was transferred to fresh medium and incubated for 6–8 h for activation of fungi. The inoculum volume was approximately 10^5-6^ CFU mL^−1^. The volume of the culture medium was 10 mL, and the incubation condition was 37°C in an air incubator. The amounts of *C. albicans* could be reached 10^7-8^ CFU mL^–1^. The *C. albicans* (100 µL) was mixed with Ni-IH-7 solutions (100 µL) in 800 µL sterile water for 3 h. Then, 100 µL of the different dilutions of mixed solution was spread evenly on Sabouraud’s medium and cultured at 37°C for 24–48 h before counting the colony-forming units.

The antibacterial effects of Ni-IH-7 were also evaluated with *E. coli* (CMCC(B)44102), *S. aureus* (ATCC 29213) and Methicillin-resistant *Staphylococcus aureus* (*MRSA*) (ATCC43300), *G. vaginalis* (ATCC14018) and metronidazole-resistant *G. vaginalis* (provided by Xishan People’s Hospital of Wuxi City) under the similar procedures as those for *C. albicans*. Initially, *E. coli*, *S. aureus* and MRSA stored at -80°C were thawed and inoculated into Luria-Bertani liquid culture medium for overnight shaking. The culture volume was 5 mL, and the incubation conditions were set at 37°C in an air incubator. The next day, *E. coli*, *S. aureus* and MRSA were transferred to fresh culture medium and incubated for 2 h to activate the bacteria. The inoculum was approximately 10^5-6^ CFU mL^−1^. The culture volume was 10 mL, and the conditions were maintained at 37 °C in an air incubator. The quantities of *E. coli*, *S. aureus* and MRSA could reach 10^7-8^ CFU mL^−1^. Mixtures of 100 µL of *E. coli*, *S. aureus* and MRSA with 100 µL of Ni-IH-7 solution were prepared in 800 µL of sterile water and incubated for 0–6 h. Subsequently, 100 µL of different dilutions of the mixture were evenly spread on Luria-Bertani agar plates, followed by incubation at 37 °C from 24 to 48 h, and colony counting assay was performed to determine antibacterial efficiency. Similarly, *G. vaginalis* stored at −80°C was thawed and inoculated into brain-heart infusion (BHI) medium. This BHI medium consisted of brain/heart infusion broth containing 1% gelatin, 1% yeast extract, 0.1% soluble starch, 0.1% glucose, and 10% fetal bovine serum, and the inoculation process occurred overnight. The volume of the medium used was 5 mL, and the incubation condition was set at 37 °C in an incubator with 5% CO_2_. Following an overnight incubation post-inoculation, the necessary amount of bacterial solution was taken and transferred into fresh medium at a ratio of 1:25. This fresh medium was then cultured at 37°C with 5% CO_2_ for 6–8 h until the OD600 reached approximately 0.5. Next, an appropriate quantity of the bacterial inoculum was centrifuged at 4300 × g for 5 min at 4 °C to replace the bacteria from the culture medium with an equivalent volume of sterile water. For the experimental setup, 100 µL of *G. vaginalis* was mixed with 900 µL of sterile water as the control group. In the experimental group, 100 µL of *G. vaginalis* was mixed with either Ni-IH-7 solutions, Ni(Ac)_2_, or metronidazole in 800 µL of sterile water. The colony counting assay was performed to determine antibacterial efficiency.

Permeability measurements were performed using the ANS uptake assay. 20 µM ANS and 10^7^ CFU mL^−1^ *C. albicans* were mixed in 0.9% NaCl and incubated in dark condition for 30 min. The centrifuged system were resuspended in 0.9% NaCl and the changes in the fluorescence emission was measured between 450–600 nm with excitation at 380 nm, using a costar blk microplate plate.

Depolarization measurements were performed using the diSC3(5) uptake assay. 20 µM diSC3(5) and 10^7^ CFU mL^−1^ *C. albicans* were mixed in 0.9% NaCl and incubated in dark condition for 30 min. The centrifuged system were resuspended in 0.9% NaCl and the changes in the fluorescence emission was measured at 670 nm with excitation at 622 nm, using a costar blk microplate plate.

### Ferroptosis measurement

10 µM BODIPY were used to investigate the lipid peroxidation of *C. albicans*. The changes in the fluorescence emission was measured at 525 nm with excitation at 488 nm. GSH/GSSG ratio in treated *C. albicans* were measured using GSH and GSSG Assay Kit.

### SEM, TEM and Confocal Microscopy for *C. albicans*

The patterns of *C. albicans* treated by Ni-IH-7 was examined by SEM and TEM. First, *C. albicans* were resuspended in Glutaraldehyde (2.5%) for 24 h at 4 °C under dark conditions. Fungal cells were then fixed on the filter paper with crawler and washed and treated with ethanol gradient dehydration, before being dried using a critical point dryer and coated with platinum sputter. Finally, SEM images were obtained on a Hitachi S-4800FE-SEM (Japan) at a working voltage of 15.0 kV and a working current of 10 µA. For TEM, the centrifuged fungi were processed by high pressure freezing and observed. The confocal microscopy of *C. albicans* were also characterized. In short, 1 µg mL^−1^ PI and 5 × 10^−6^ M Syto9 in 0.9% NaCl staining solution were added to the centrifuged fungi and incubated for 25 min avoiding light. Next, the samples were washed twice with 0.9% NaCl before being analyzed using ZIESS-LSM700 (Germany).

### RNA-Seq for Transcriptome Analysis and Metabolome Analysis and Real-time PCR verification

The supernatants were removed from fungi cultured in Sabouraud’s broth following centrifugation at 4 °C at 10,000 × g for 6 min and the precipitation was collected. After washing twice with 0.9% NaCl, the *C. albicans* were stored at −80°C. Bacteria were processed for transcriptome analysis and metabolome analysis. Total RNA was isolated using the Trizol Reagent (Invitrogen Life Technologies, China), after which the concentration, quality and integrity were determined using a NanoDrop spectrophotometer (Thermo Scientific, USA). Total RNA extraction, RNA sequencing and bioinformatic data collection were performed by Shanghai Personal Biotechnology Cp. Ltd (Shanghai, China). Real-time(RT)-qPCR was performed to quantify mRNA. A total of 1 μg RNA was reverse transcribed into cDNA using HiScript III RT SuperMix for qPCR Kit (Vazyme) according to the manufacturer’s instructions. qPCR was performed in the QuantStudio real-time PCR system (Applied Biosystems) with Taq Pro Universal SYBR qPCR Master Mix Kit (Vazyme). Reactions were carried out for 30 s at 95 °C, followed by 40 cycles of two-step PCR for 10 s at 95 °C, 30 s at 60 °C. The relative level of the target gene was determined using the 2^−delta delta Ct^ analysis method. TEF3 was applied as the endogenous control.

### Antifungal activity of Ni-IH-7 against vaginal secretions from patients with fungal infection

Vaginal secretions from patients with fungal infection were provided by Xishan People’s Hospital of Wuxi City. They were dissolved in 0.9% NaCl and treated by Ni-IH-7 (400 µg mL^−1^, 3 h). Columbia blood plates were used for microbial counting. This study was approved by the Ethics Committee of Wuxi Xishan People’s Hospital (approval number: LLS2020ky036 27/07/2020) and obtained informed consent from all patients. The ages of human participants are 35, 42, 46, 48, 27, 36, 41, 31, 26, and 47, respectively.

### The application of Ni-IH-7 in medical pads

Medical polylactic acid fiber pads were provided by Xishan People’s Hospital of Wuxi City. In short, The pads were soaked in nanotube solution for 10–30 min and dried in a 60 °C for more than 6 h. Pads attached to nanotubes were used for antifungal testing.

### Cytotoxicity and hemolysis analyzes

For cytotoxicity assay, VK2 cells (CRL-2616, obtained from ATCC) were planted in 96-well plates (2 × 10^4^ cells per well) and cultured with 5% CO_2_ at 37 °C overnight. Then the culture medium was replaced by fresh DMEM medium with 10% fetal bovine serum (FBS) and 1% penicillin and streptomycin (PS), 100, 200, 500, 1000 μg mL^−1^ of Ni-IH-7 were added to incubate for another 24 h. Then the medium containing materials was removed and cell viability was measured using a CCK8 Kit.

Hemolysis assay was performed by using fresh animal blood from 10-week-old c57BL/6 rats (all animals were housed in a temperature-controlled (25 ± 2 °C) condition (treatment of humidity from 40–70%) with normal light, and were given free access to water and diet). First, 0.5 mL of red blood cells (RBC) were resuspended in 5 mL of saline. Then, RBC suspensions (0.2 mL) were added into 0.8 mL of Ni-IH-7 saline dispersions with different concentrations (100, 200, 500, and 1000 μg mL^−1^). The mixtures were incubated at 37 °C for 2 h. Ultrapure water and saline were used as the positive control and negative control, respectively. Finally, the absorbance of the supernatant was measured at 540 nm after centrifugation at 3000 g for 15 min. The ratio of hemolysis was calculated by the formula:

3 $${{{{{\rm{Hemolysis\; ratio}}}}}}(\%)=({{{{{{\rm{A}}}}}}}_{s}-{{{{{{\rm{A}}}}}}}_{N})/({{{{{{\rm{A}}}}}}}_{P}-{{{{{{\rm{A}}}}}}}_{N})\times 100\%$$

where A_*S*_ represents the absorbance of RBCs exposed to Ni-IH-7, A_*N*_ represents the absorbance of RBCs exposed to saline, and A_*P*_ represents the absorbance of RBCs exposed to ultrapure water. All assays were performed as triplicates.

### Statistics and reproducibility

Each experiment, including TEM, STM, Cryo-EM, AFM, SEM, CD, NMR, UV-VIS, FTIR, XRD, XPS, confocal microscopy imaging, cell viability assay, hemolysis analyzes, and all antibacterial experiments, was independently repeated at least three times. Quantitative analysis was conducted using GraphPad Prism 9.0.0, and all values and error bars were represented by calculating the mean ± SD. Dot plots and bar charts were analyzed using an unpaired Student’s two-sided *t*-test to obtain the *p*-values. The ClusterProfiler (version 3.4.4) software was employed for the enrichment analysis of the KEGG pathway of differential genes, with a focus on pathways significantly enriched with a *p*-value < 0.05. (ns means no significant difference, *p < 0.05, **p < 0.01, ***p < 0.001, and ****p < 0.0001).

### Reporting summary

Further information on research design is available in the Nature Portfolio Reporting Summary linked to this article.

### Supplementary information

Supplementary Information

Peer Review File

Description of Additional Supplementary Files

Supplementary Data 1

Supplementary Movie 1

Supplementary Movie 2

Reporting Summary

### Source data

Source Data

## Data Availability

All data are available in the main text or Supplementary Materials. All electron microscope images are available upon request to the corresponding authors. Source data is available for Figs. 4–7, Supplementary Fig. 5, 8, 10, 11, 14–28, 30–36, 38, 41, 43–47, 49–52, 55 and 56. All primers used in this study can be found in Supplementary Data 1. Source data are provided with this paper
