# Supplementary material for: Stable peptide-assembled nanozyme mimicking dual antifungal actions
Source: Nat Commun. 2024 Jul 5;15:5636. doi: 10.1038/s41467-024-50094-6 (PMC11224359; doi:10.1038/s41467-024-50094-6)
Supplement: Supplementary file 1 — Supplementary Information [file 41467_2024_50094_MOESM1_ESM.pdf]

Stable peptide-assembled nanozyme mimicking dual antifungal actions

By Ye et al,

This file contains 57 figures and 9 tables.

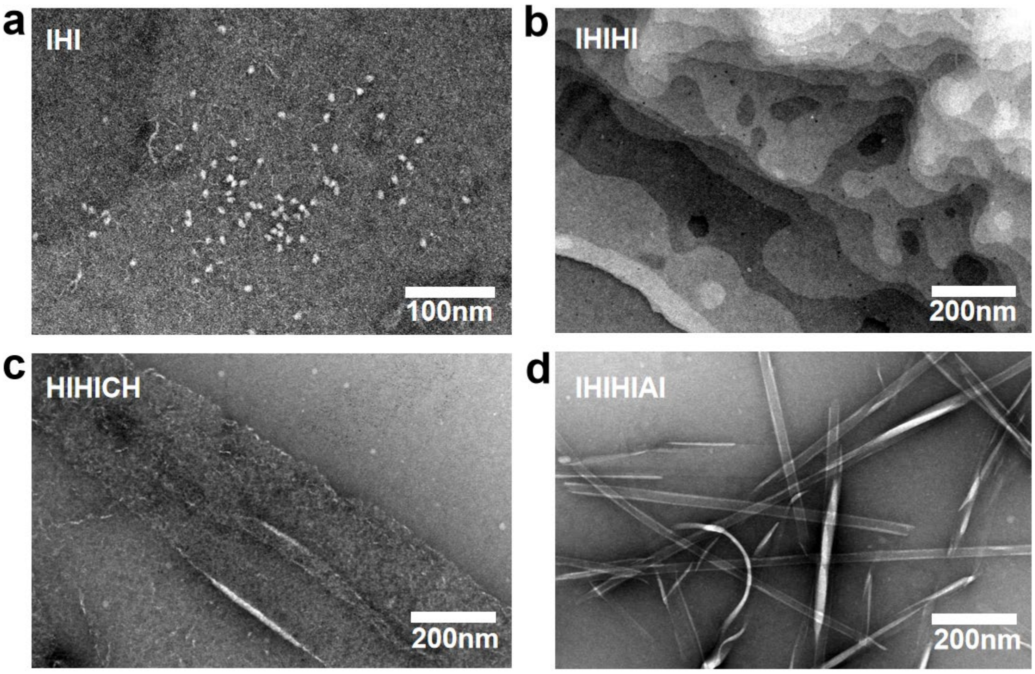

Supplementary Fig. 1 TEM characterization of peptide assembly with different lengths. a IHI. **b** IHIHI. **c** HIHICH. **d** IHIHIAI. Three times each experiment was repeated independently with similar results. Representative images are shown.

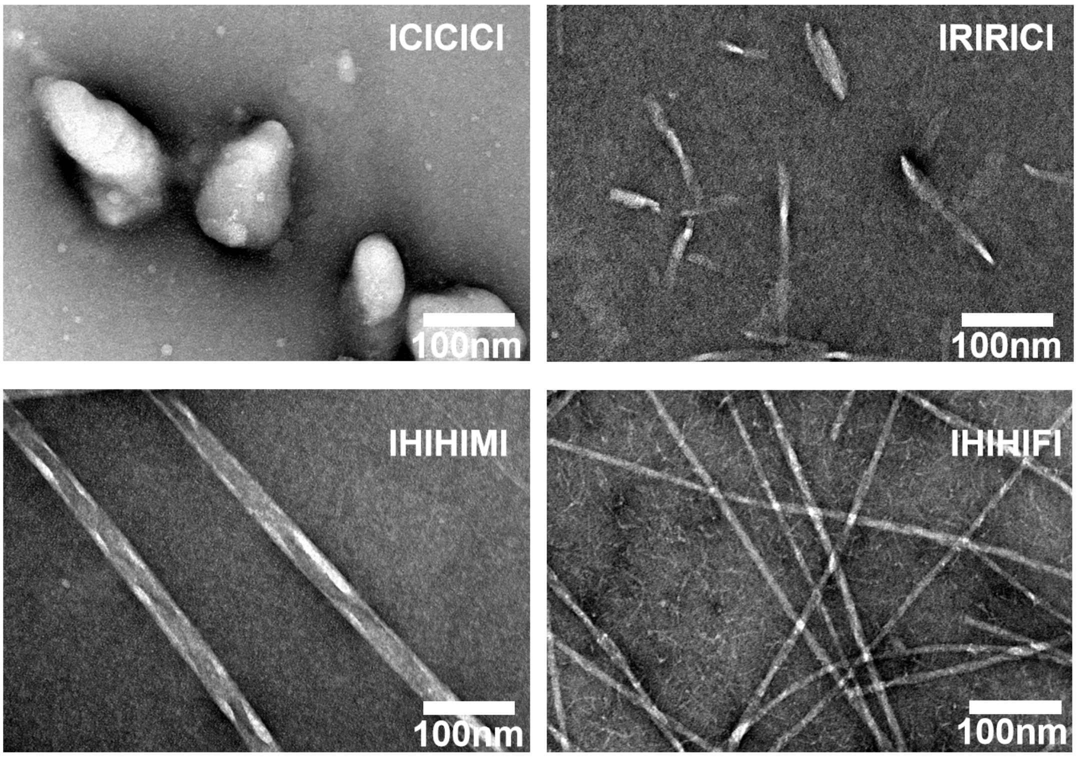

Supplementary Fig. 2 TEM characterization of heptapeptides with different sequences. Three times each experiment was repeated independently with similar results. Representative images are shown.

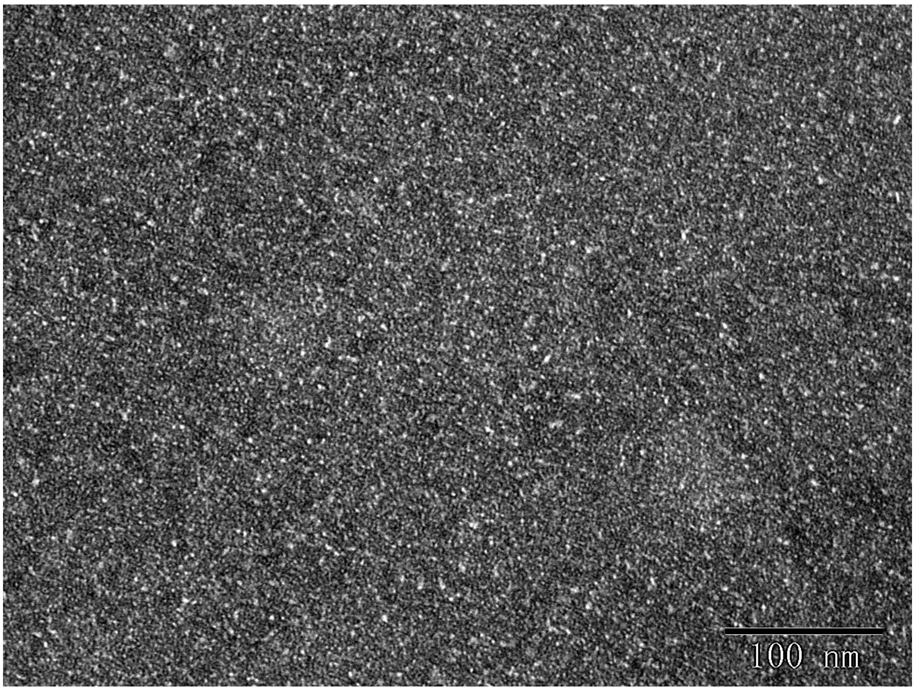

Supplementary Fig. 3 TEM characterization of IPIPICI assembly. Three times each experiment was repeated independently with similar results. Representative images are shown.

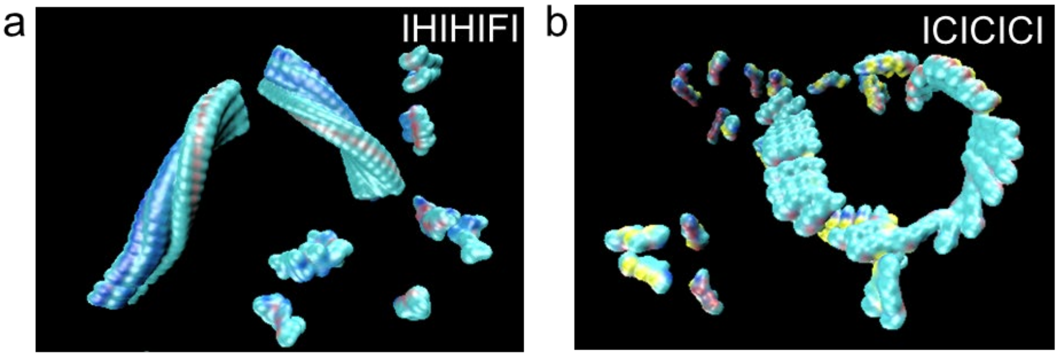

Supplementary Fig. 4 AlphaFold2 predicted assembly pattern of IHIHIFI (a) and ICICICI (b). Both of their assembled nanostructure have been characterized by TEM in Supplementary Fig. 2.

Supplementary Table 1 The pLDDT scores, provided by AlphaFold2, are utilized to forecast the trajectory of peptide self-assembly.

Sequence	pLDDT
IHI (60X)	<60
IHIHI (60X)	<60
IHIHIFI (60X)	~80
IHIHIHICI (60X)	~60
ICICICI (60X)	<60
IRIRICI (60X)	~80
AAAAAAA (60X)	<60
IHIHIHI (60X)	~80
IHIHICI (60X)	~80
IHIHICI (12X)	<60
IHIHICI (4X)	~60
HIHICIH	<60
HIHIHIC	~60
IIIIIII	<60

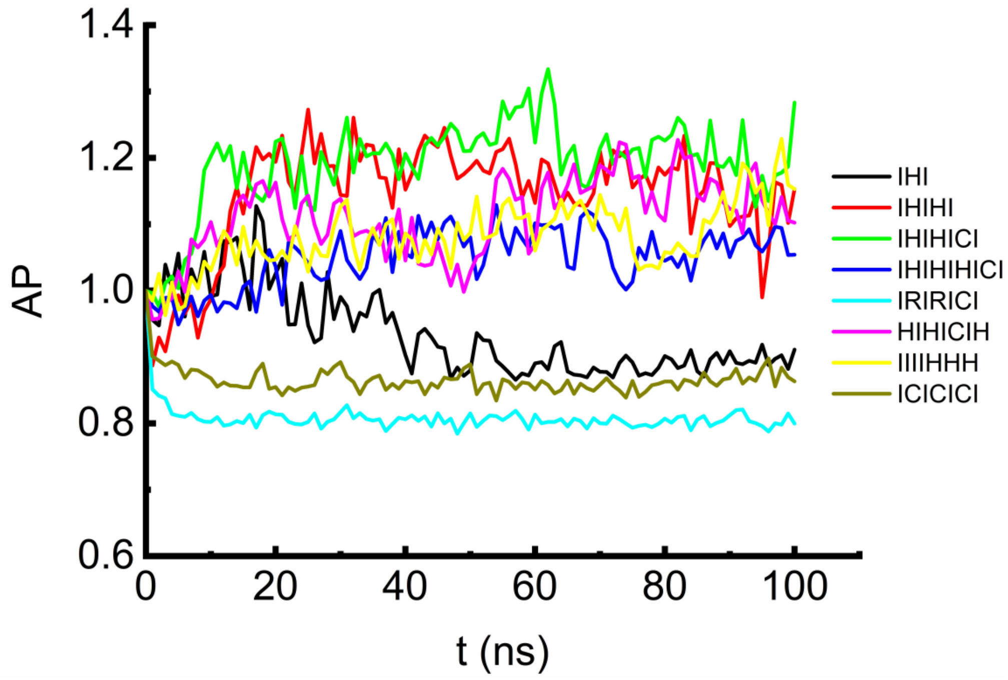

Supplementary Fig. 5 AP as a function of hydrophobicity for different peptides.

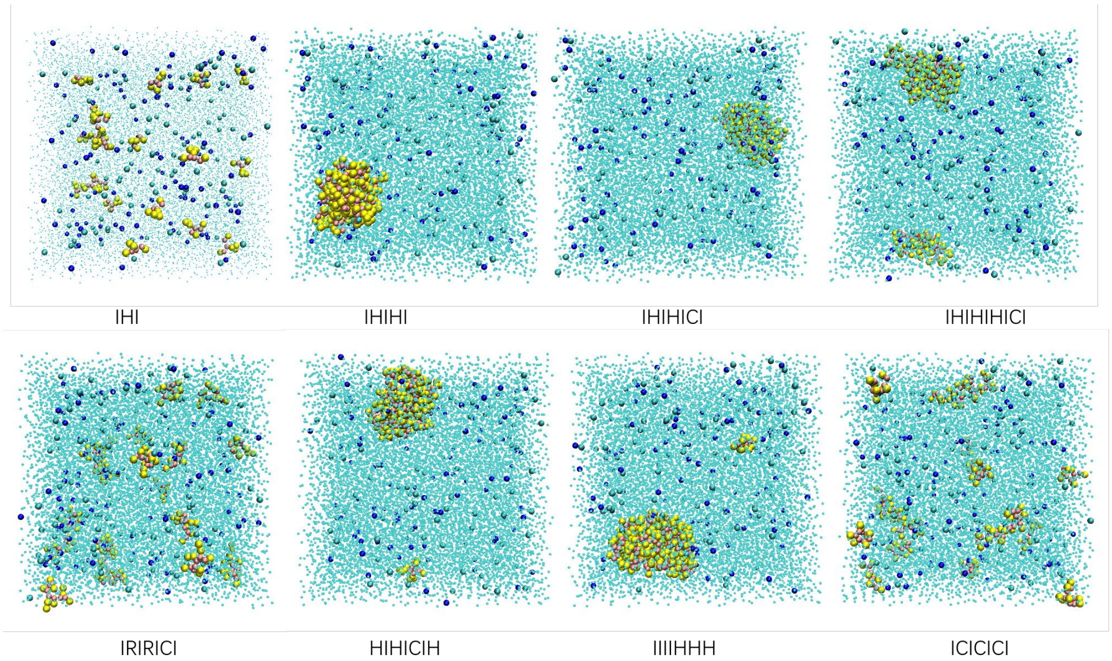

Supplementary Fig. 6 GCMD simulation results (100 ns) for different peptides, showing various levels of aggregation.

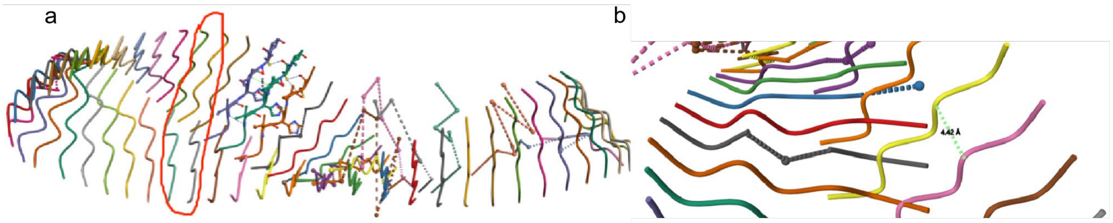

Supplementary Fig. 7 Structure predication with AlphaFold2. a, Possible three-dimensional structures of IHIHICI predicted by AlphaFold2. **b,** The separation distance between two IHIHICI monomers by AlphaFold2.

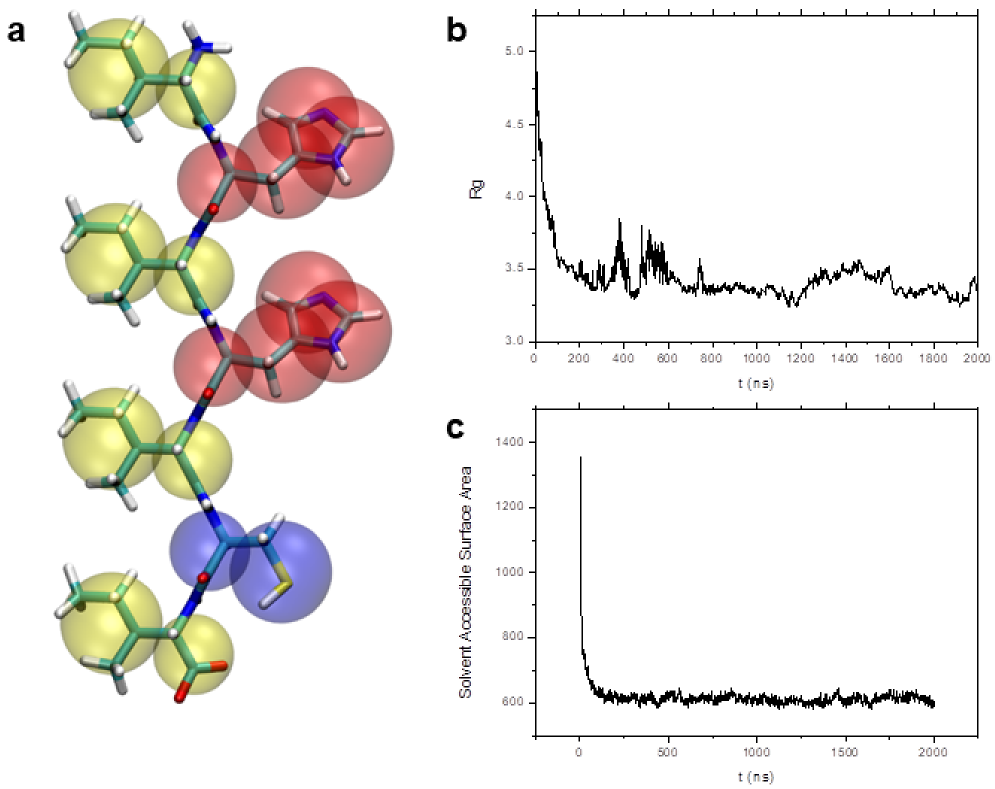

Supplementary Fig. 8 Data quality analysis of GCMD by Ni-IH-7. a The group granulation model of single IHIHICI. **b** Rg and **c** SASA of MD of the granulation model. The Rg of the overall polypeptide decreases rapidly, and the different structures of the aggregates evolve, and the Rg changes continuously until the final columnar system was about 3.3 nm. The solvent-contactable area of SASA decreases rapidly with the progress of assembly, and the solvent-contactable area reaches a stable value after the formation of large aggregates, indicating that the molecules in the whole system have formed stable fibrous aggregates at this time.

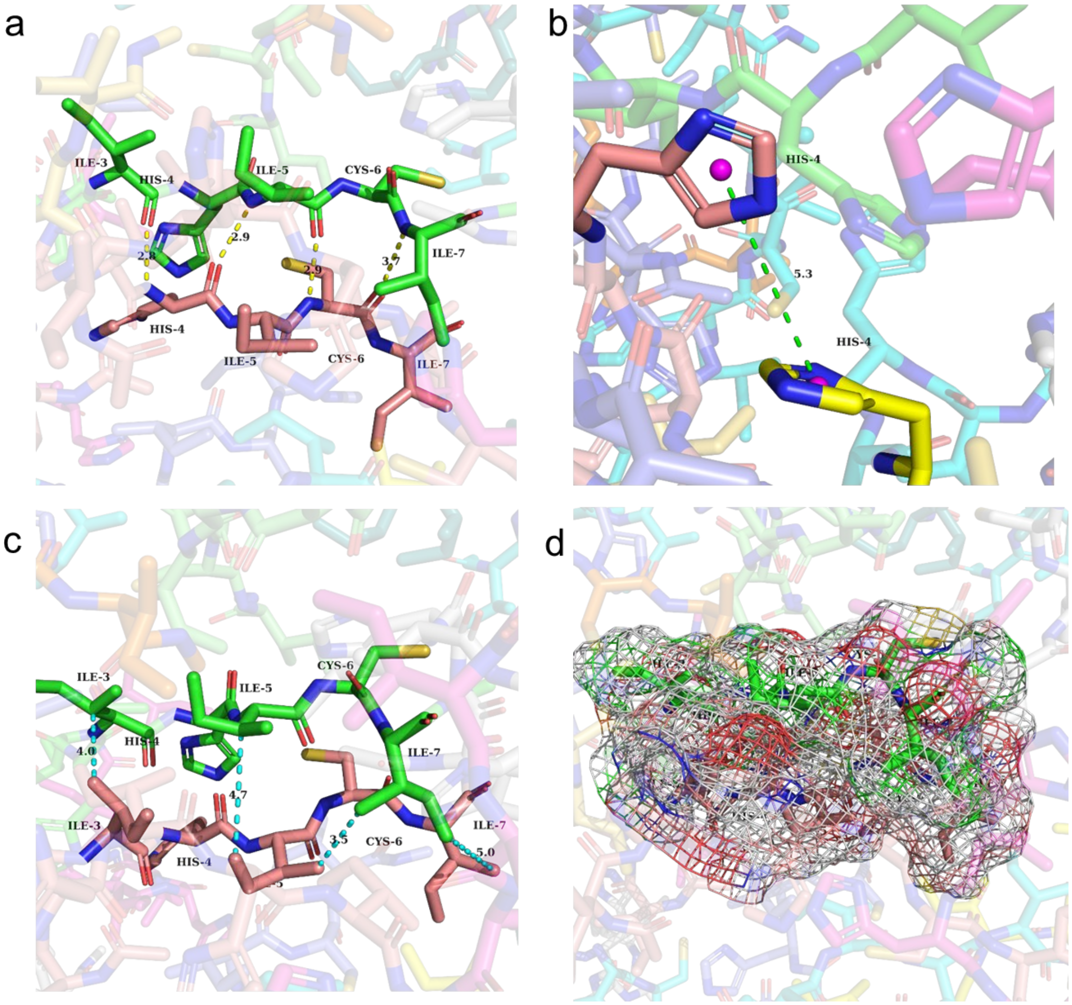

Supplementary Fig. 9 Molecular mechanism of peptides self-assembly. a, hydrogen bonds. **b,** π-π stacking. **c,** hydrophobic interaction. **d,** vdW interaction using the analysis of the rdg (reduced density gradient). The atomic proximity between peptides showed a significant presence of van der Waals interactions (green area). Hydrogen bonds are primarily formed between the carbonyl groups (hydrogen bond acceptors) and the amino groups in the amide bonds (hydrogen bond donors) in the peptide backbones, with the bond distances ranging from 2.8 to 4 Å. The π-π stacking mainly occurs between the aromatic ring side chains of histidine residues in the peptides. Furthermore, the hydrophobic interactions come from the side chain of the isoleucine residues in the heptapeptide. There are multiple van der Waals contacts.

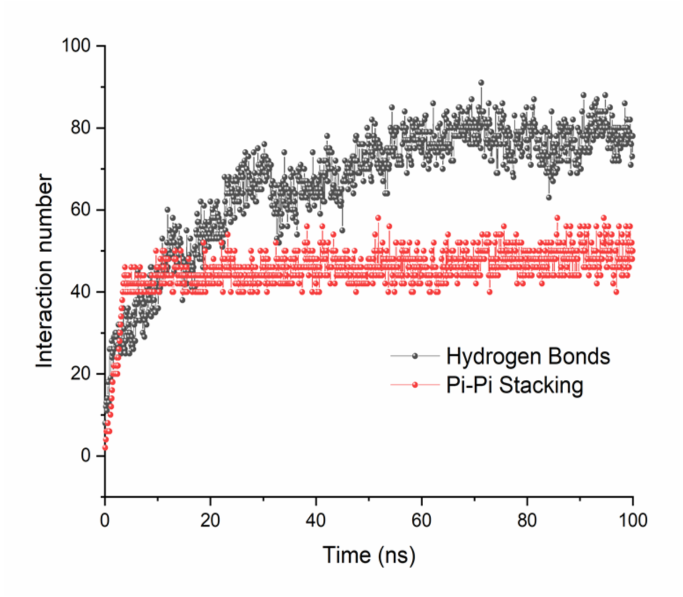

Supplementary Fig. 10 The counting number of hydrogen bonds and π-π stacking in IH-7 assembly (20x).

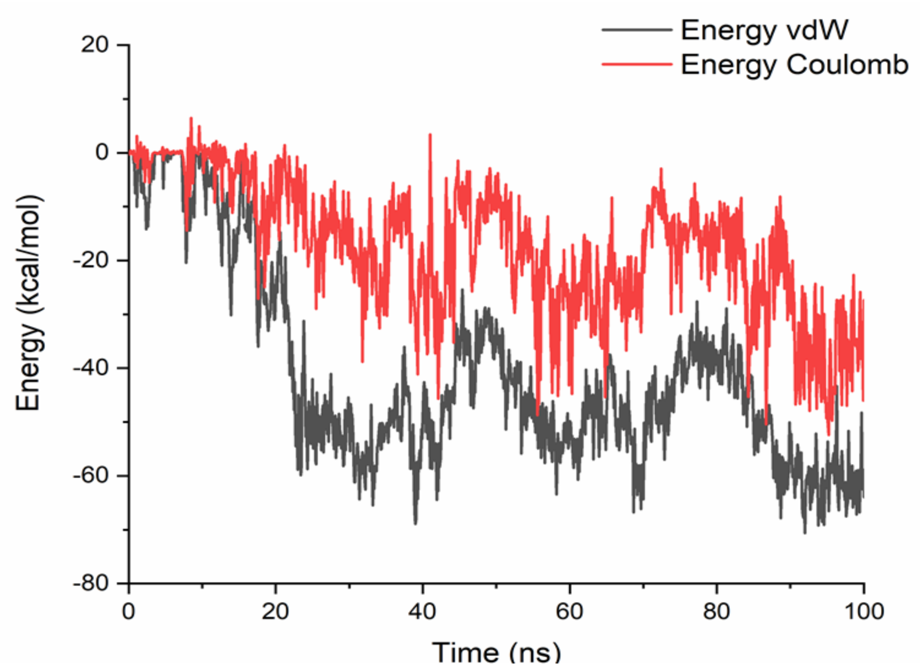

Supplementary Fig. 11 The trends of the interaction energy (vdW and Coulomb) between peptides during the self-assembly process. In a 100 ns simulation, the van der Waals interaction energy was -63.995 kcal/mol, and the electrostatic interaction energy was -27.35 kcal/mol.

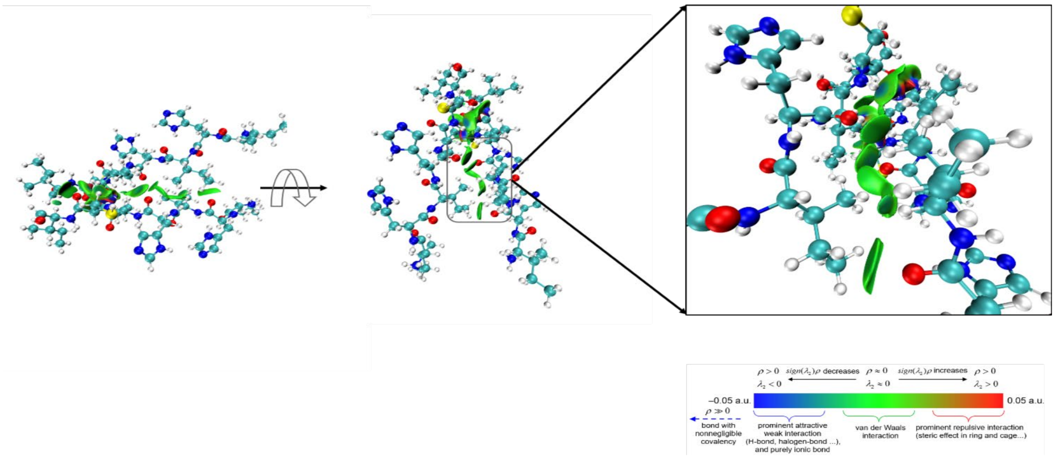

Supplementary Fig. 12 DFT analysis to ensure the distribution of van der Waals weak interactions during IH-7 peptides aggregation process.

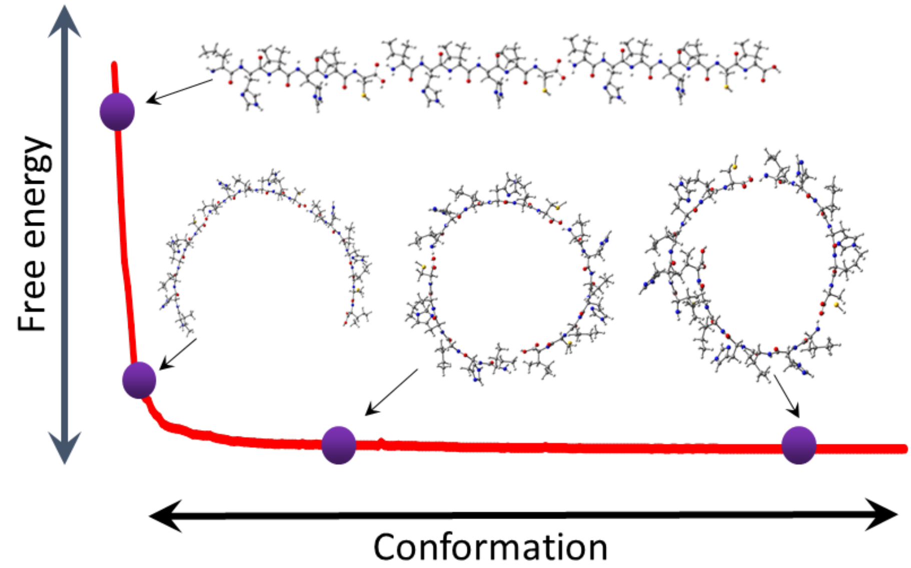

S

Supplementary Fig. 13 The DFT calculations confirm the tendency of IHIHICI oligomers to grow in the curved direction.

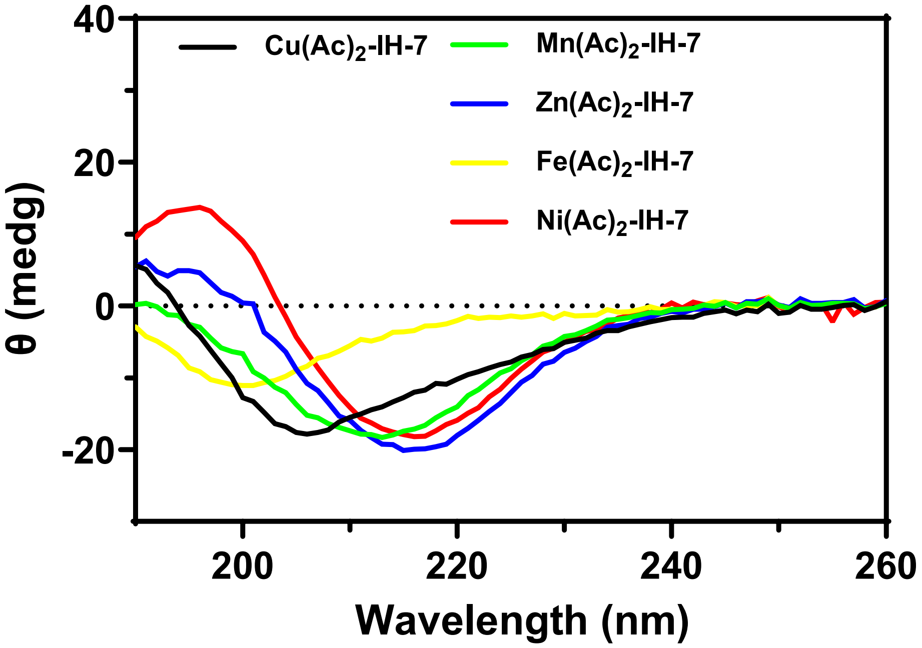

Supplementary Fig. 14 CD spectra of IH-7 in acetate solutions containing different metal ions.

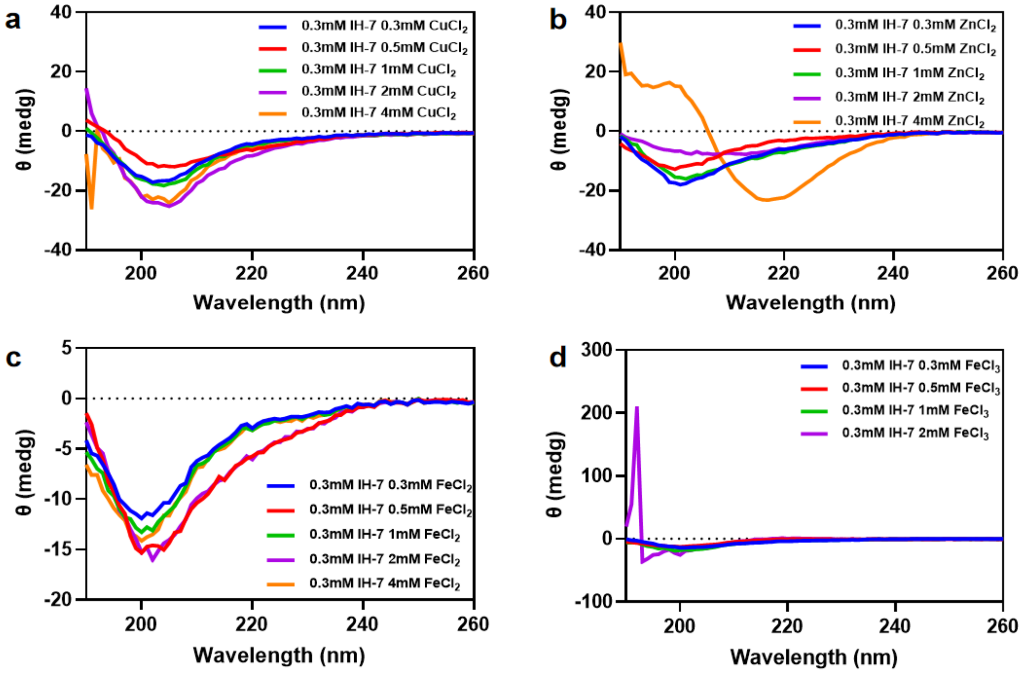

Supplementary Fig. 15 CD spectra of IH-7 in different concentrations of metal ions solutions. **a** $CuCl_2$. **b** $ZnCl_2$. **c** $FeCl_2$. **d** $FeCl_3$.

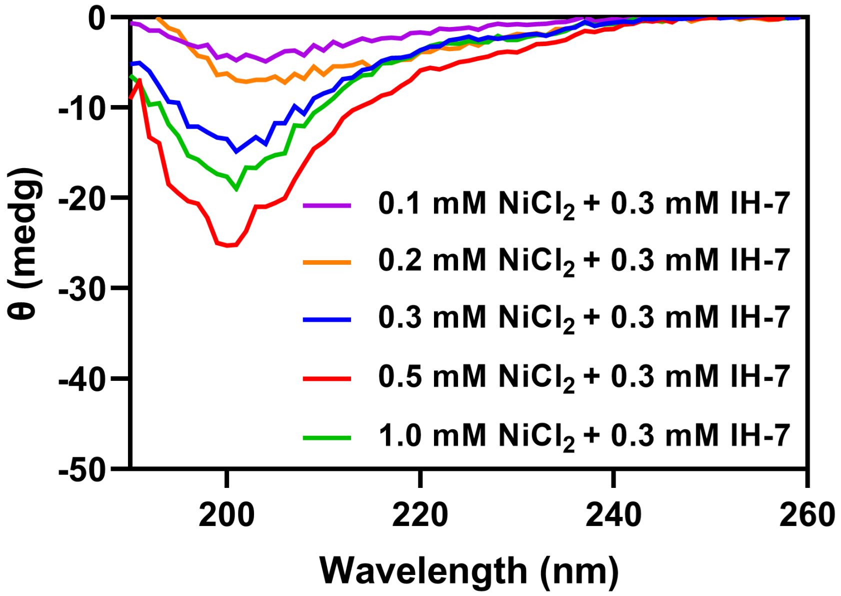

Supplementary Fig. 16 CD spectra of IH-7 in different concentrations of Ni(Cl)$_2$ solutions.

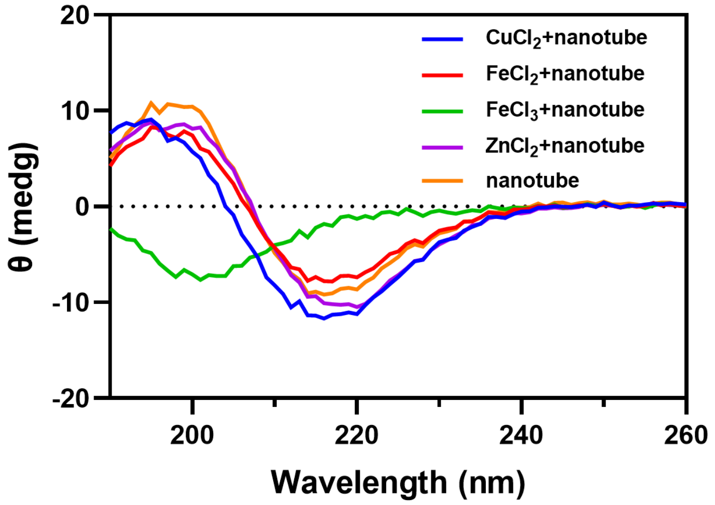

Supplementary Fig. 17 CD spectra of NH$_4$Ac-nanotube in different metal ions solutions.

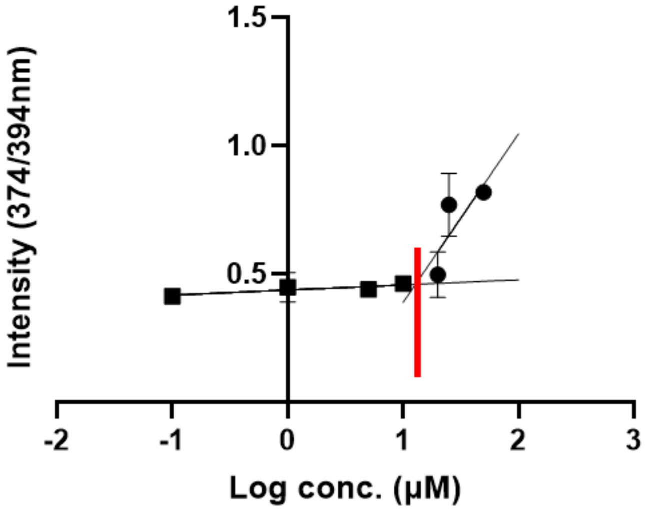

Supplementary Fig. 18 The CMC of IH-7 using pyrene probe.

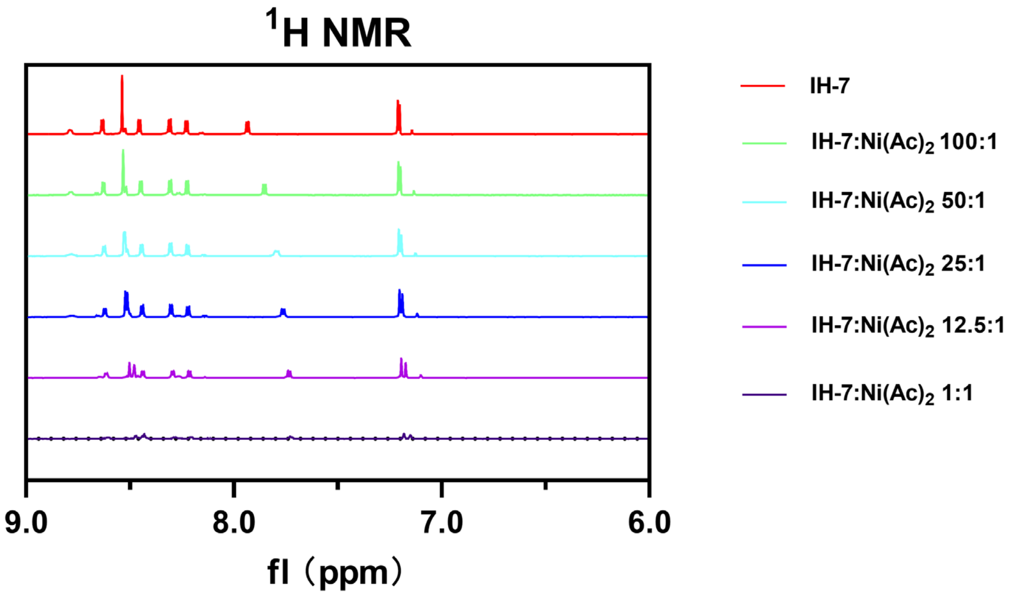

Supplementary Fig. 19 NMR spectra of IH-7 with different ratios of nickel acetate.

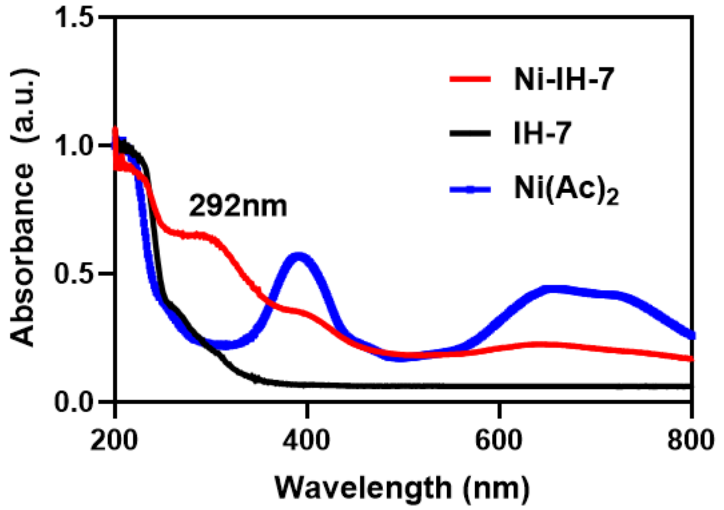

Supplementary Fig. 20 UV-VIS of Ni-IH-7 from 200-800nm.

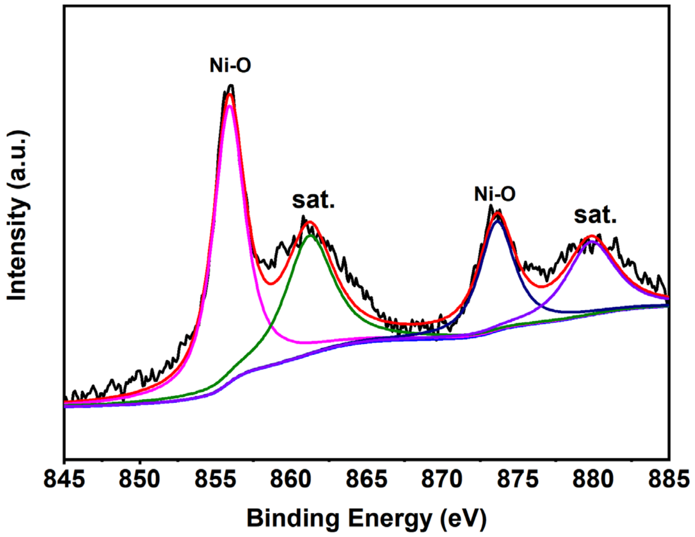

Supplementary Fig. 21 Ni *2p* peak of Ni-IH-7 from XPS.

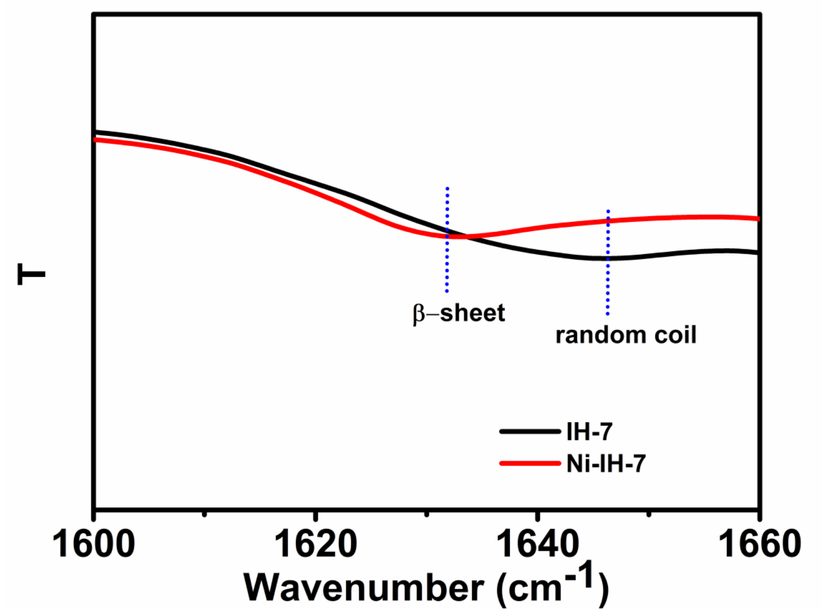

Supplementary Fig. 22 FTIR spectra of IH-7 and Ni-IH-7 demonstrating the occurrence of secondary structural transitions.

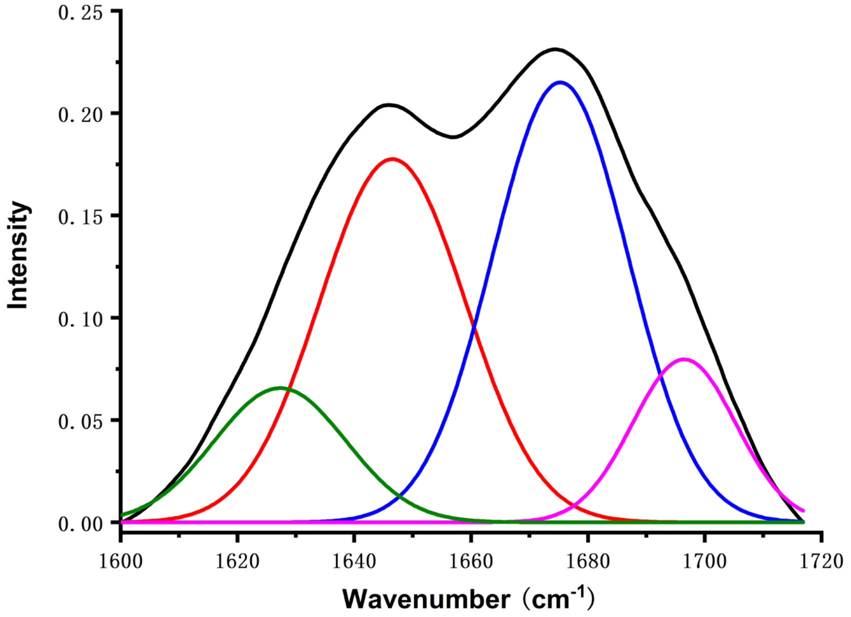

Supplementary Fig. 23 The FTIR of the amide I region, ranging from 1,600 to 1,700 cm^{-1} of IH-7 was fit by multiple Gaussian peaks.

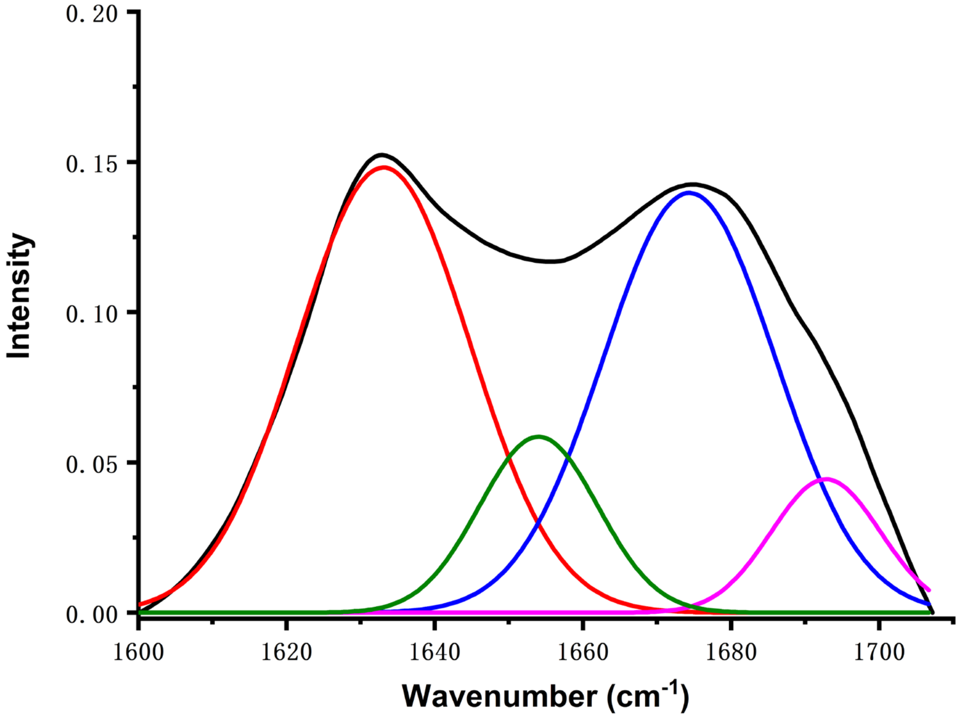

Supplementary Fig. 24 The FTIR of the amide I region, ranging from 1,600 to 1,700 cm^{-1} of Ni-IH-7 was fit by multiple Gaussian peaks.

Generally, highly ordered β-sheets yield amide I bands at 1 610-1 640 cm^{-1} and antiparallel β-sheets yield bands at 1 660-1 695 cm^{-1}, whereas the α-helix pattern occurs at 1,650-1,660 cm^{-1}, random coils and other turn structures show bands at 1 640-1 650 cm^{-1}.

Supplementary Table 2 The percent of different secondary structures in IH-7.

Wavenumber (cm^{-1})	Structure	Peak (cm^{-1})	Percent (%)
1610-1640	β-sheet	1627	12.00
1640-1650	Random coils	1646	36.10
1650-1660	α-helix	/	/
1660-1695	Antiparallel β-sheet	1675	40.50
		1696	11.40

Supplementary Table 3 The percent of different secondary structures in Ni-IH-7.

Wavenumber (cm^{-1})	Structure	Peak (cm^{-1})	Percent (%)
1610-1640	β-sheet	1633	41.70
1640-1650	Random coils	/	/
1650-1660	α-helix	1654	11.30
1660-1695	Antiparallel β-sheet	1674	39.20
		1692	7.80

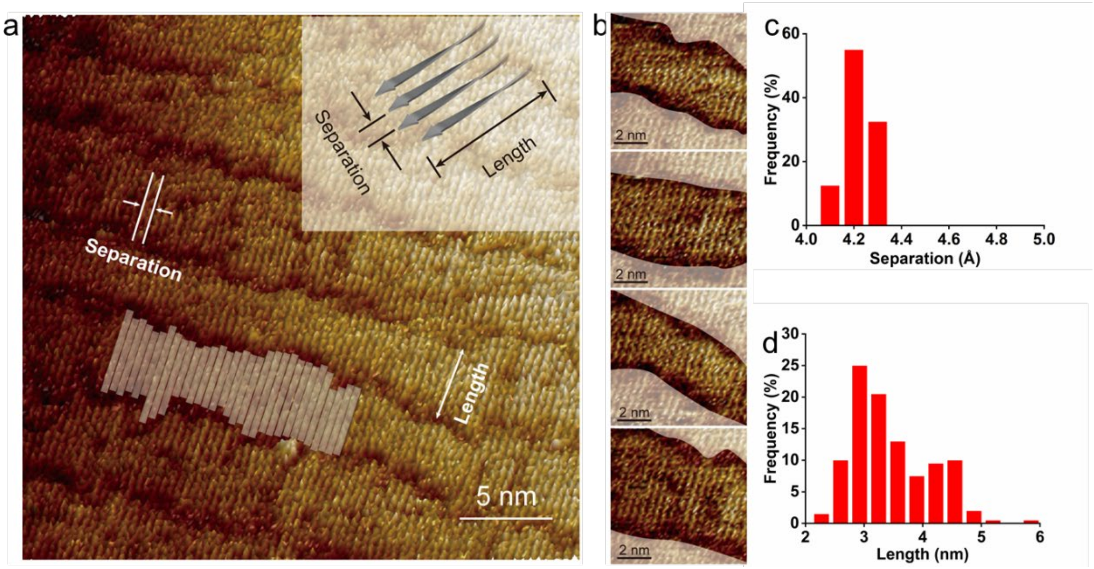

Supplementary Fig. 25 STM characterization of Ni-IH-7 nanotubes. a, representative STM image of the Ni-IH-7 nanotube. **b,** Partial enlarged view of Fig. a. **c,** The separation distance between two adjacent IHIHICI strands. **d,** Data statistics for each individual length of IHIHICI. The results determined that the regions represented by some rectangles are different IHIHICI monomers. Three times each experiment was repeated independently with similar results. Representative images are shown.

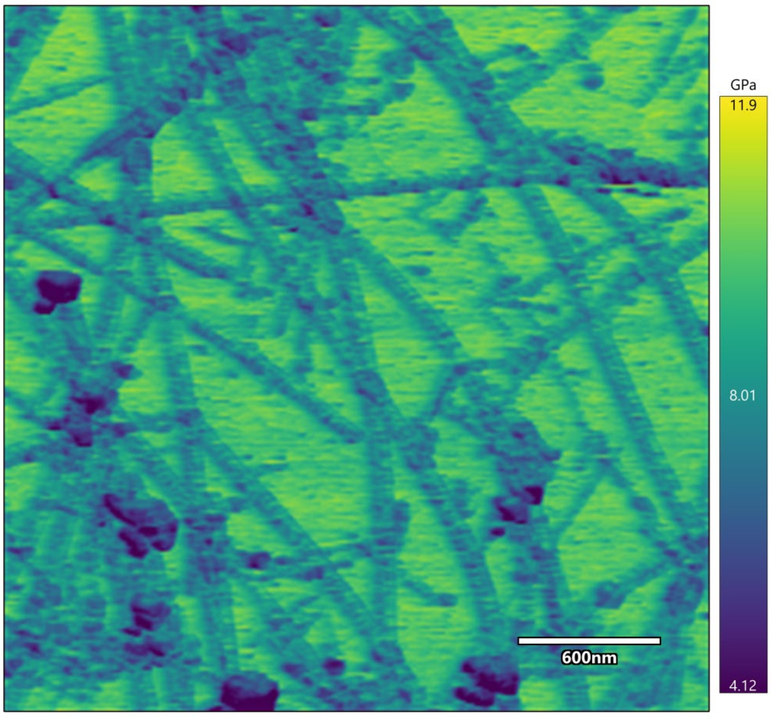

Supplementary Fig. 26 AM-FM characterization of Ni-IH-7 morphology. Three times each experiment was repeated independently with similar results. Representative images are shown.

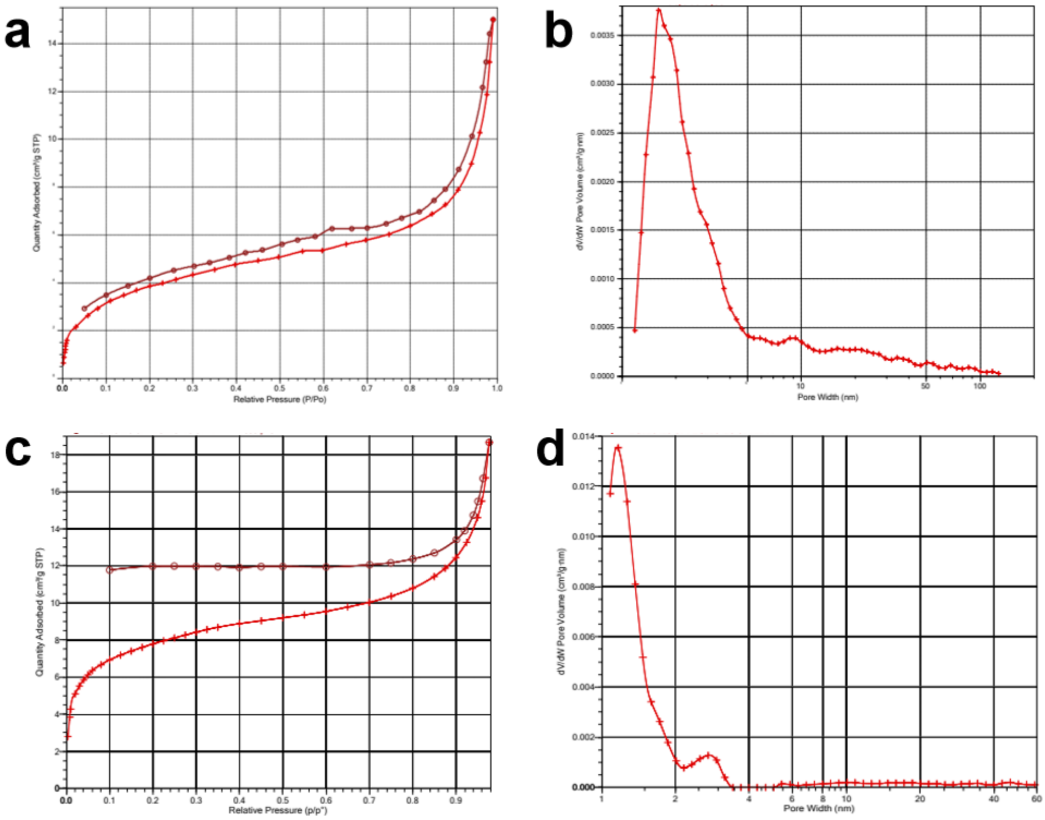

Supplementary Fig. 27 The pore size analysis of Ni-IH-7 using physical adsorption. a Nitrogen physisorption isotherms. **b** Mesopore size distributions of the Ni-IH-7 nanotubes. **c** Argon physisorption isotherms. **d** Micropore size distributions of the Ni-IH-7 nanotubes. The micropore

distribution is obtained using NLDFT analysis.

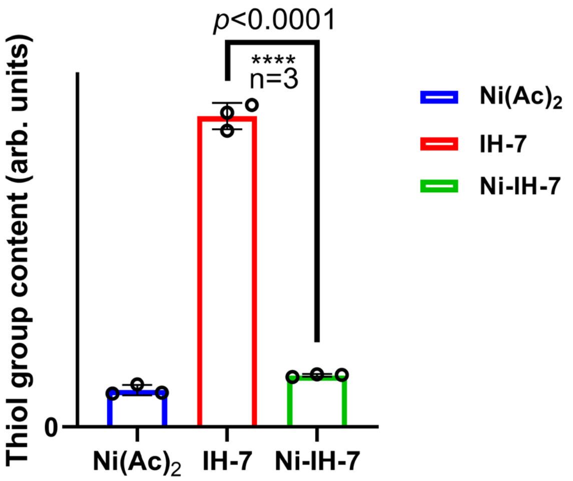

Supplementary Fig. 28 The state of the cysteine in the Ni-IH-7 nanotubes measured by a Micro Total Mercapto Assay Kit. The significant difference was evaluated by a two-tailed unpaired t-test. n = 3 independent samples, bars represent means ± SD, ****p < 0.0001.

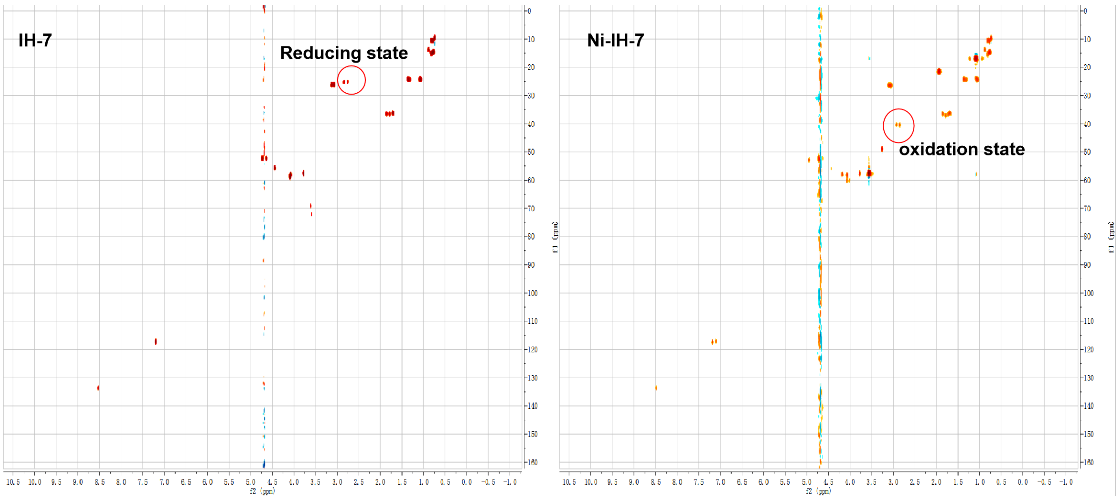

Supplementary Fig. 29 1H-13C 2D NMR of IH-7 and Ni-IH-7. The Cys of C_β could be seen and the oxidation and reduction states have been labeled.

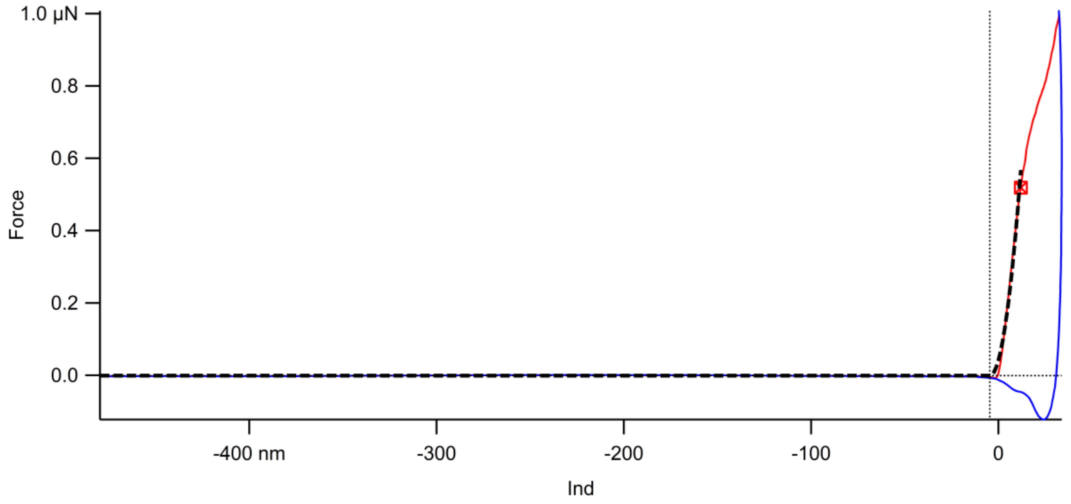

Supplementary Fig. 30 Hertz curves of Ni-IH-7.

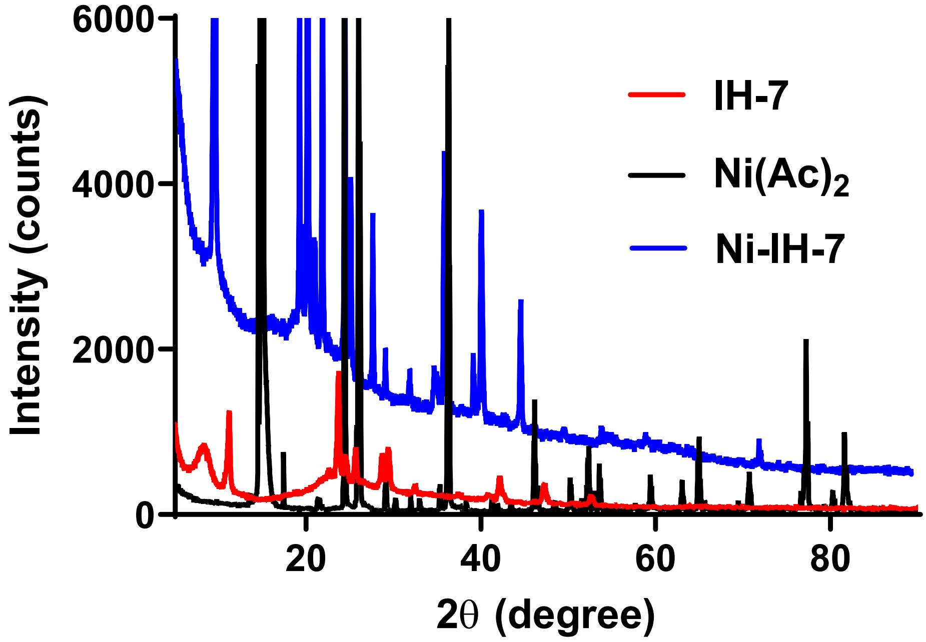

Supplementary Fig. 31 The corresponding PXRD of each component showing the formation of ordered organic nanotubes.

Supplementary Table 4 Coordination Analysis of Ni-IH-7 analyzed by EXAFS.

Nickel salt	Morphology
Basic nickel carbonate	Irregular
Nickel carbonate	Irregular
Nickel hypophosphite	Irregular
Nickel formate	Irregular filament and nanotube

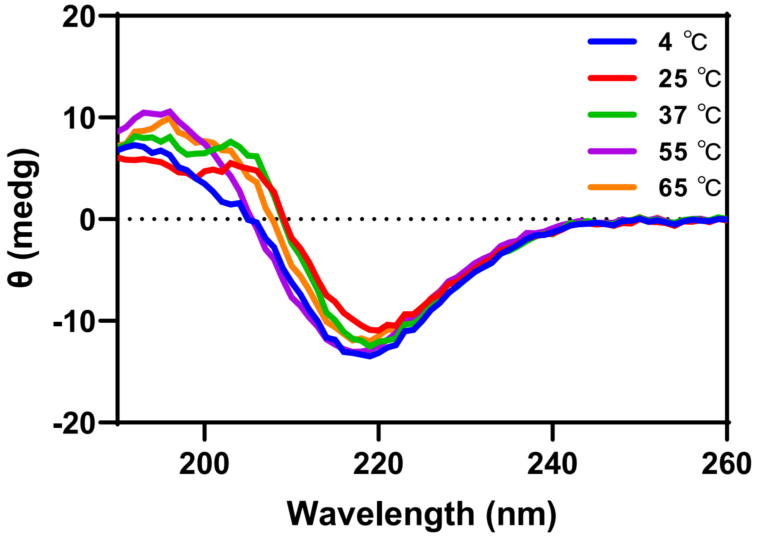

Supplementary Fig. 32 CD spectra of Ni-IH-7 co-assembly at different constant temperatures.

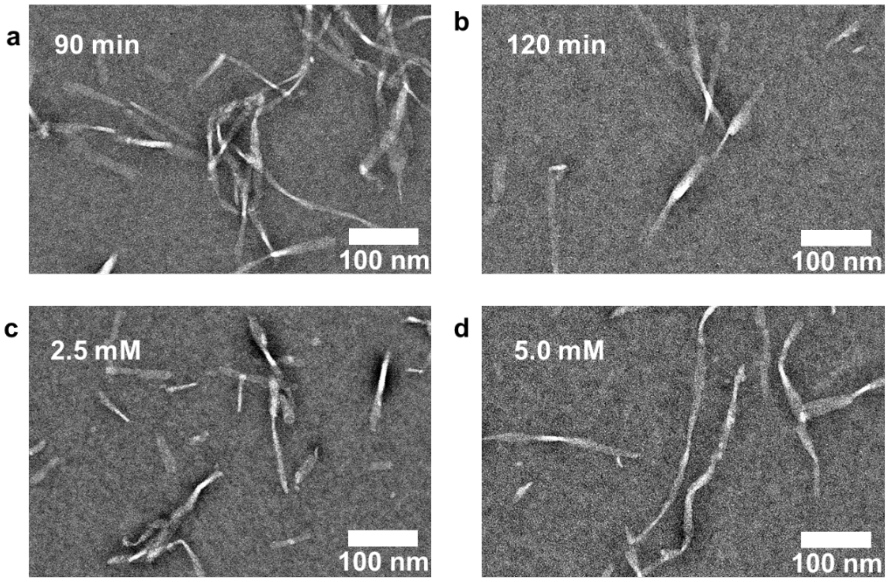

Supplementary Fig. 33 The morphology of IRIRICI with Nickle Acetate at different assembly time and concentrations. a 90 min. b 120 min. c 2.5 mM. d 5.0 mM.

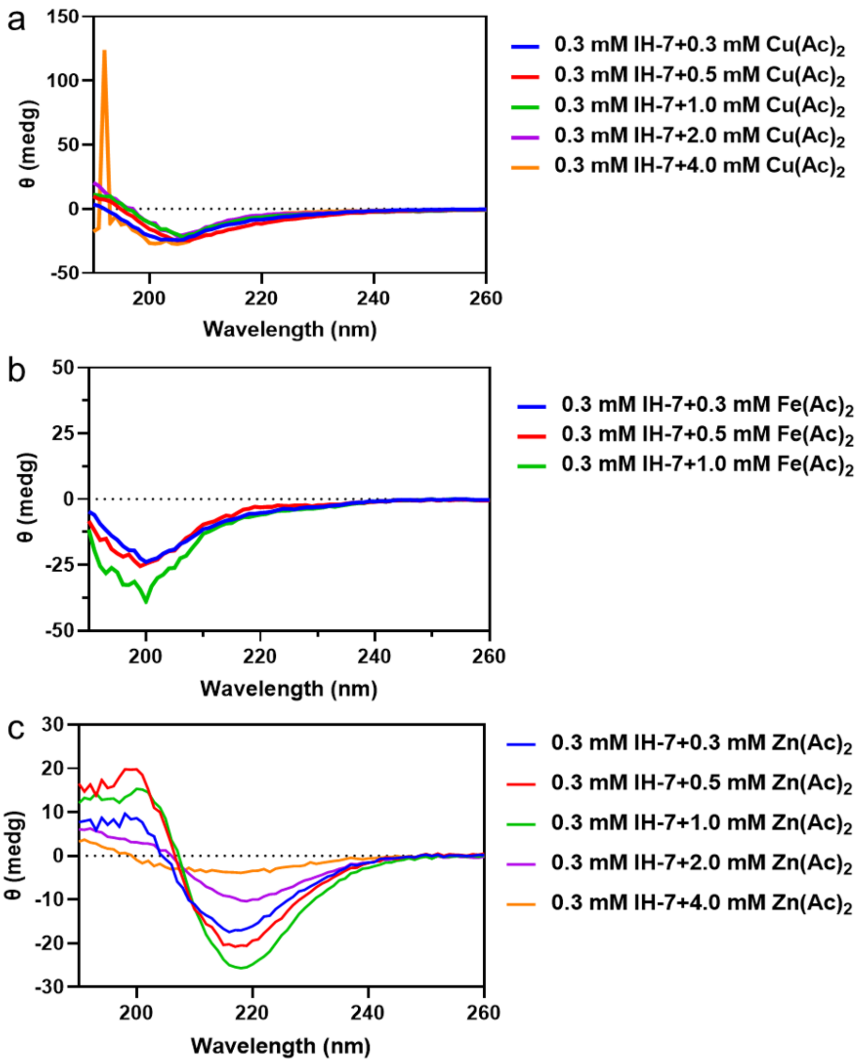

Supplementary Fig. 34 CD spectra of acetate-IH-7 co-assembly at different concentrations of acetate salts. **a** $Cu(Ac)_2$. **b** $Fe(Ac)_2$.**c** $Zn(Ac)_2$.

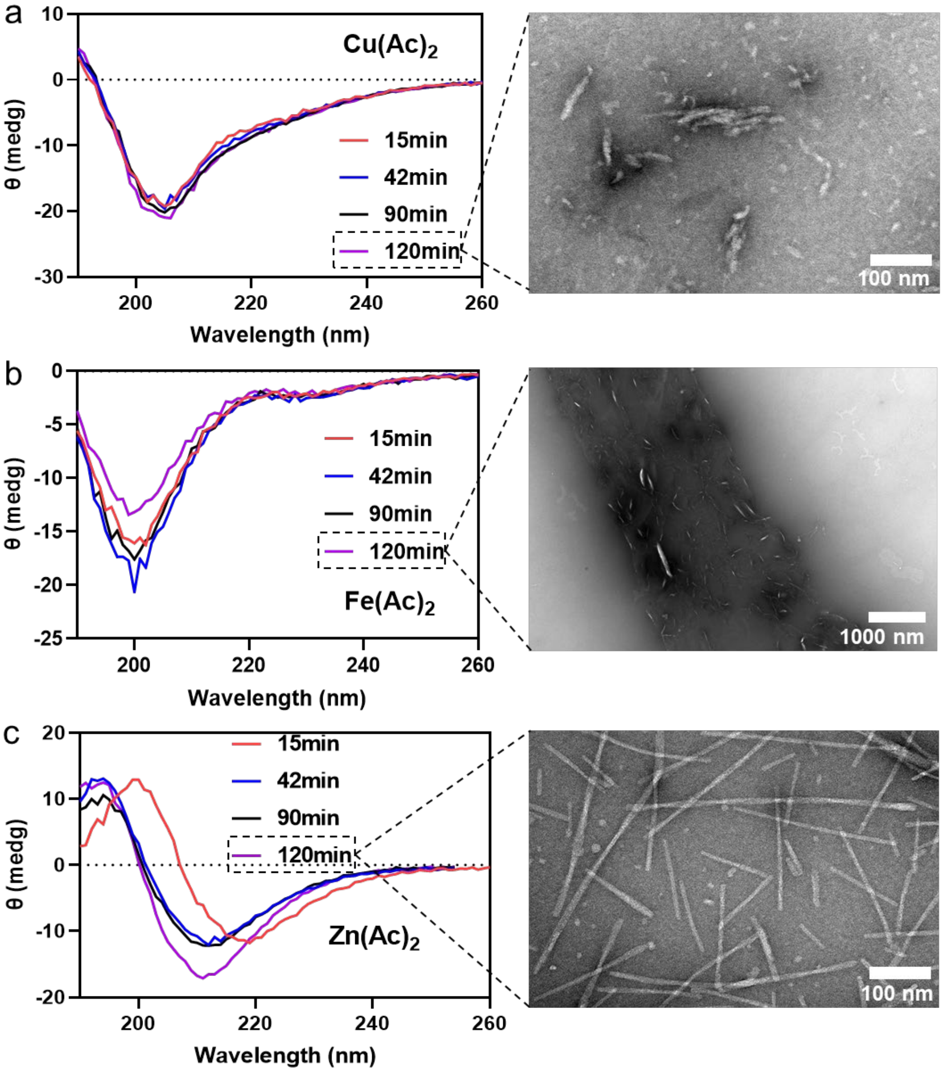

Supplementary Fig. 35 CD spectra (Left) and TEM (Right, the samples with 120 min sonication) characterization of acetate-IH-7 co-assembly at different ultrasonic time. a $Cu(Ac)_2$. **b** $Fe(Ac)_2$.**c** $Zn(Ac)_2$.

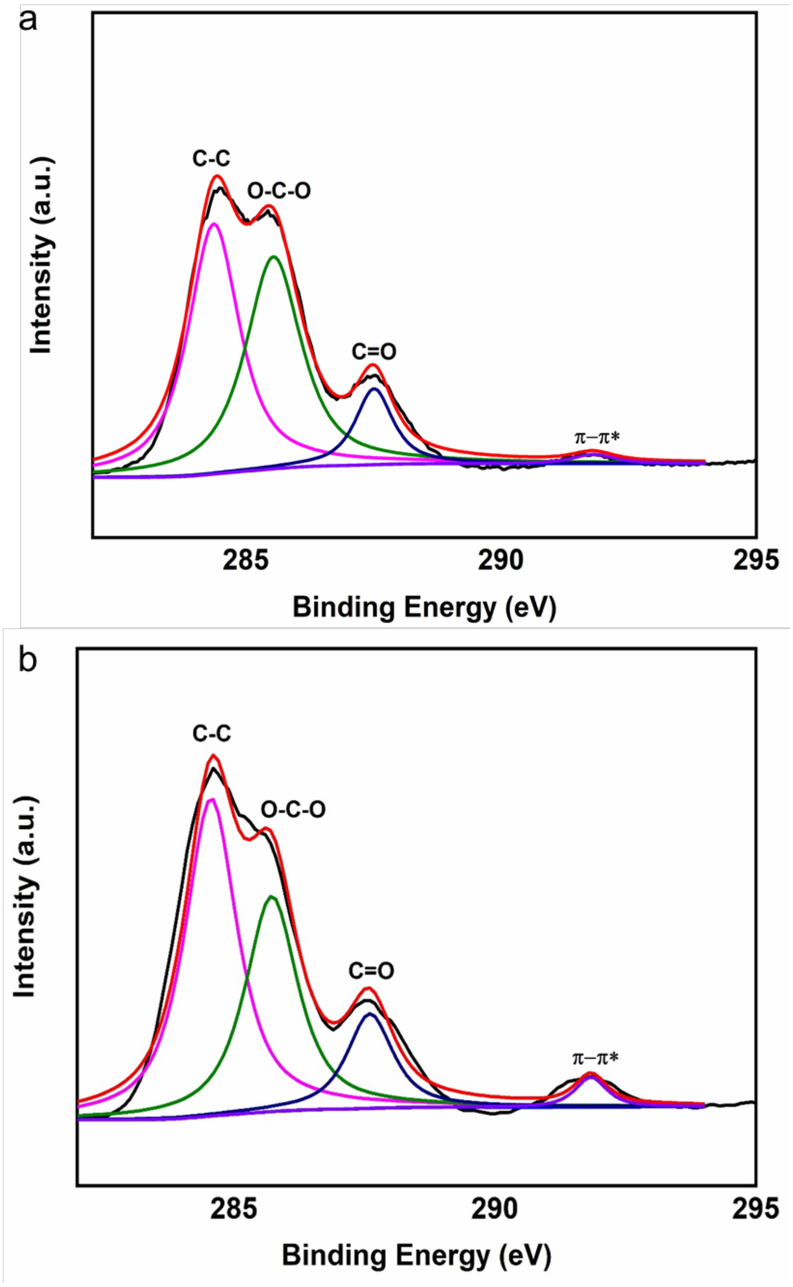

Supplementary Fig. 36 The π-π analysis of Ni-IH-7 and IH-7. a C *1s* peak of IH-7 from XPS. **b** C *1s* peak of Ni-IH-7 from XPS.

Supplementary Table 5 IH-7 and Ni-IH-7 C *1s* peak splitting results statistics.

Functional groups types	IH-7（%）	Ni-IH-7（%）
C-C	46.13	50.13
O-C-O	42.39	34.01
C=O	10.22	13.02
π-π	1.25	2.84

Supplementary Table 6 Coordination Analysis of Ni-IH-7 analyzed by EXAFS.

^{a}N: coordination numbers; ^{b}R: bond distance; $^{c}\sigma^2$: Debye-Waller factors; ^{d} ΔE_0: the inner potential correction. *R* factor: goodness of fit. S_0^2 was set to 0.77, according to the experimental EXAFS fit of Ni foil reference by fixing CN as the known crystallographic value; δ: percentage.

	shell	CN	R(Å)	σ^2	ΔE_0	R factor
Ni foil	Ni-Ni	12	2.48±0.01	0.0060	6.3±0.3	0.0013
sample	Ni-N(O)	6.1±0.5	2.07±0.02	0.0121	1.3±2.0	0.0133

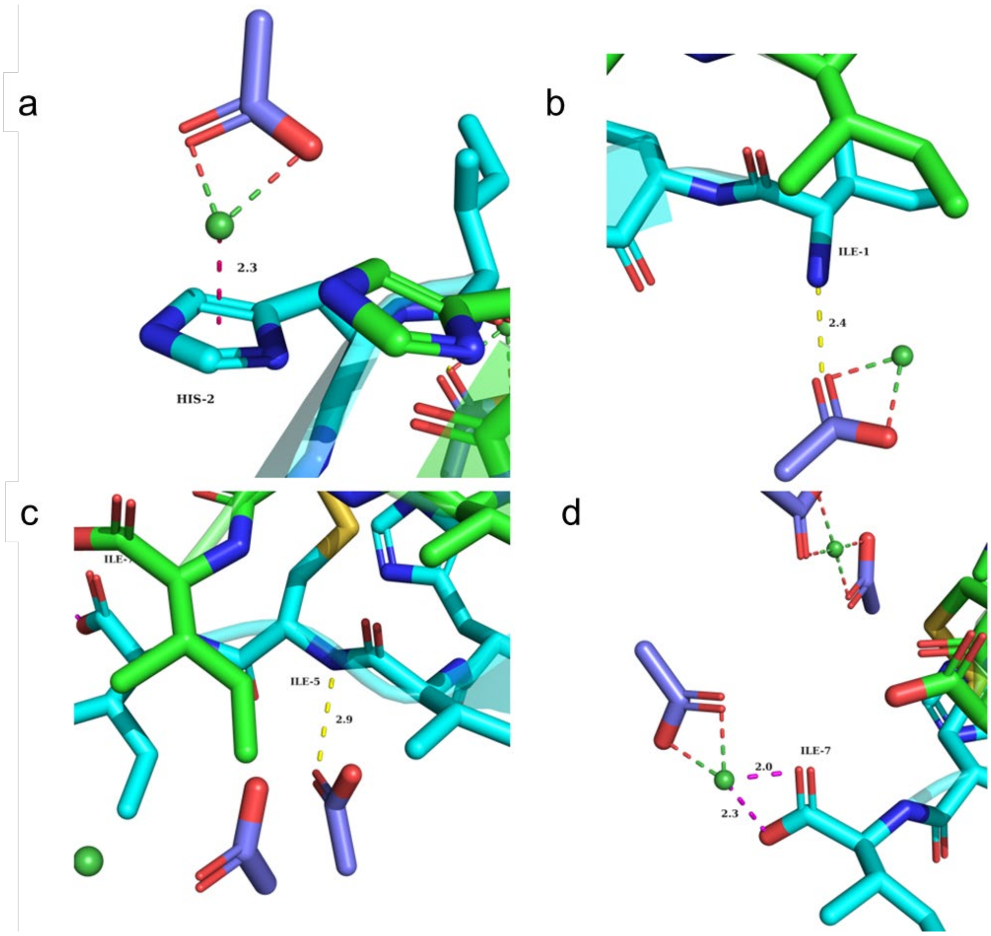

Supplementary Fig. 37 The analysis of interaction of IH-7 and $Ni(Ac)_2$. a, cation-π stacking. **b** and **c**, H bonding. **d**, metal coordination interaction. Docking experiments were supplemented to further demonstrate the coordination between Ni^{2+} and IH-7. In panel a, it is illustrated that the nickel ion in the nickel acetate molecule forms a cation-π stacking interaction with the aromatic ring of His2 at a distance of 2.3 Å. Panel b depicts the hydrogen bonding interaction between the acetate ion in nickel acetate and the amino end of Ile1, with a distance of 2.4 Å. Panel c shows a hydrogen bonding interaction at a distance of 2.9 Å between the amide group of Ile5 and the acetate ion. In Panel d, the nickel ion in the intramolecular acetate forms a metal coordination interaction with the carboxyl end of Ile7, at distances of 2.0 Å and 2.32 Å

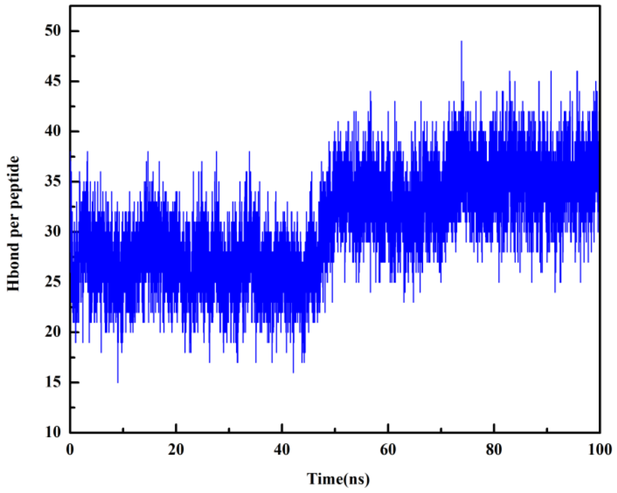

Supplementary Fig. 38 Hydrogen bond per peptide with the continuous extension of assembly time.

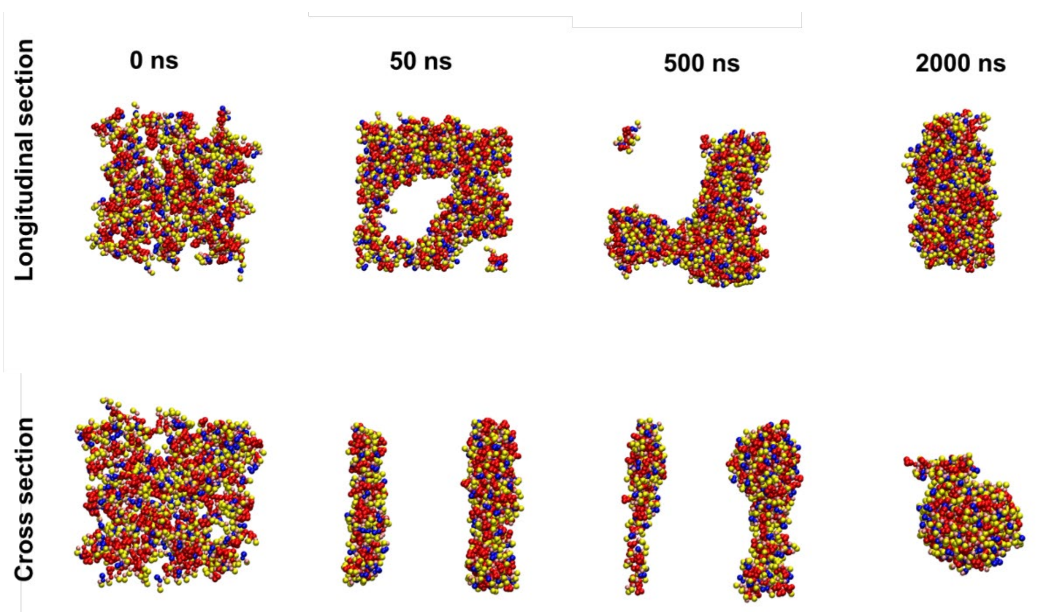

Supplementary Fig. 39 MD simulations of dynamic assembly into macroscopic structure of IHIHICI in the presence of NH_4Ac.

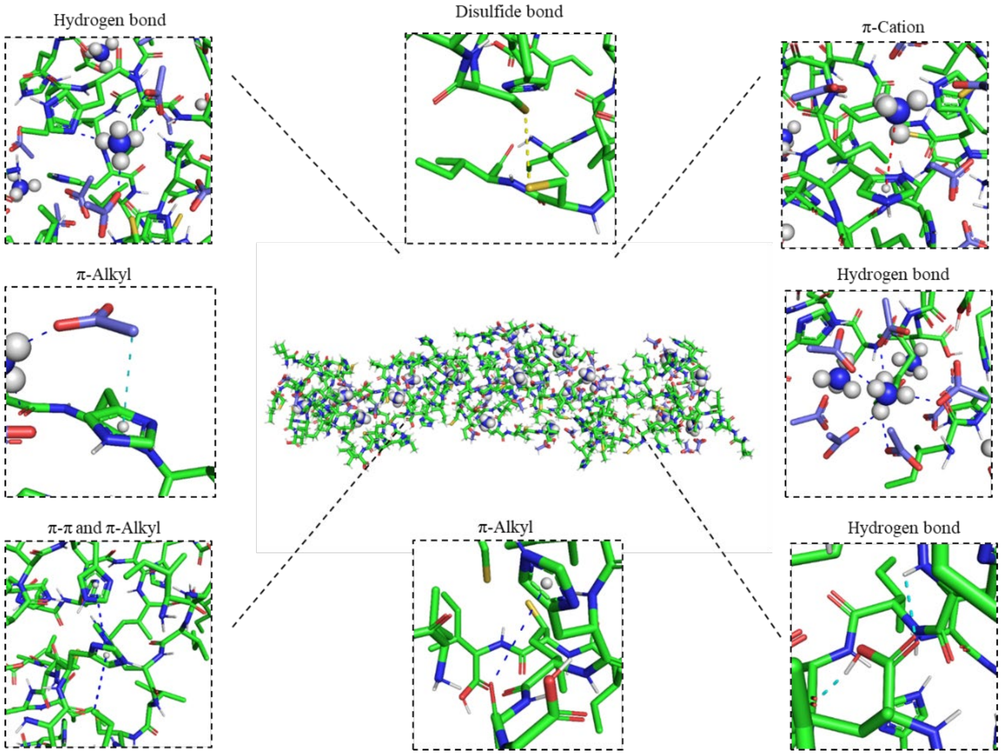

Supplementary Fig. 40 Schematic diagram of the predicted helical tubular structure and snapshots of possible atomic interactions in assembly of IHIHICI with NH_4Ac.

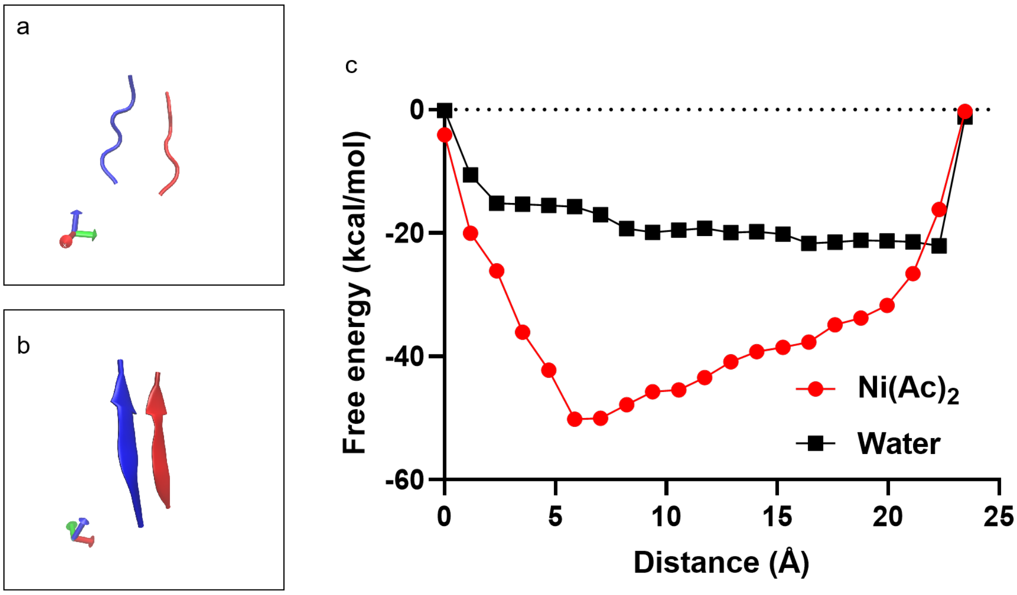

Supplementary Fig. 41 The stretching dynamics simulations for IH-7 and Ni-IH-7. a, Initial structural model of peptides under water conditions. **b,** Initial structural model of peptides under nickel acetate conditions. **c,** Free energy profiles of the dissociation of two Initial conformation formed with peptides in different solutions.

The changes in the interaction forces (free energy) between free peptides and Ni-containing peptides during the stretching dynamics were calculated. As shown in Supplementary Fig. 38, the

top left image (Supplementary Fig. 38a) represents the initial model of peptides in an aqueous phase, the bottom left image (Supplementary Fig. 38b) represents the initial model of peptides in nickel acetate. The curve in the graph shows the trend of force with distance. As Supplementary Fig. 38c illustrates, the data indicate that the maximum force required to separate peptides in nickel acetate is -50 kcal/mol, whereas in an aqueous phase, the maximum force required to separate peptides is -22 kcal/mol. The environment of nickel acetate requires a greater force to separate the peptides, meaning that nickel acetate is more likely to promote peptide assembly, whereas the aqueous environment requires relatively less force, indicating that peptide aggregation or self-assembly is relatively less likely in an aqueous phase. Gibbs free binding energy (ΔGgibbs) is used to reveal the self-assembly thermodynamics of the co-assembly possess of nickel acetate and IH-7. lower ΔGgibbs represents higher stability of the assembled superstructure, and vice versa. As shown in Supplementary Table 6, the co-assembly of Ni-IH-7 can involve the following steps: 1. Interactions between monomers; 2. Interactions between oligomers formed from monomers and $Ni(Ac)_2$ after the initial interactions between monomers; 3. Interaction between $Ni(Ac)_2$ and monomers. The results provide a simple dynamics of the co-assembly of IH-7 and $Ni(Ac)_2$, indicating that the peptide first forms a complex with $Ni(Ac)_2$ before potentially combining with monomeric IH-7.

Supplementary Table 7 ΔG_{gibbs} calculations for different assembly systems.

	IH-7-IH-7	IH-7-IH-7-$Ni(Ac)_2$	IH-7-$Ni(Ac)_2$-IH-7
ΔG_{gibbs} (kcal/mol)	-110.245	-118.282	-137.822

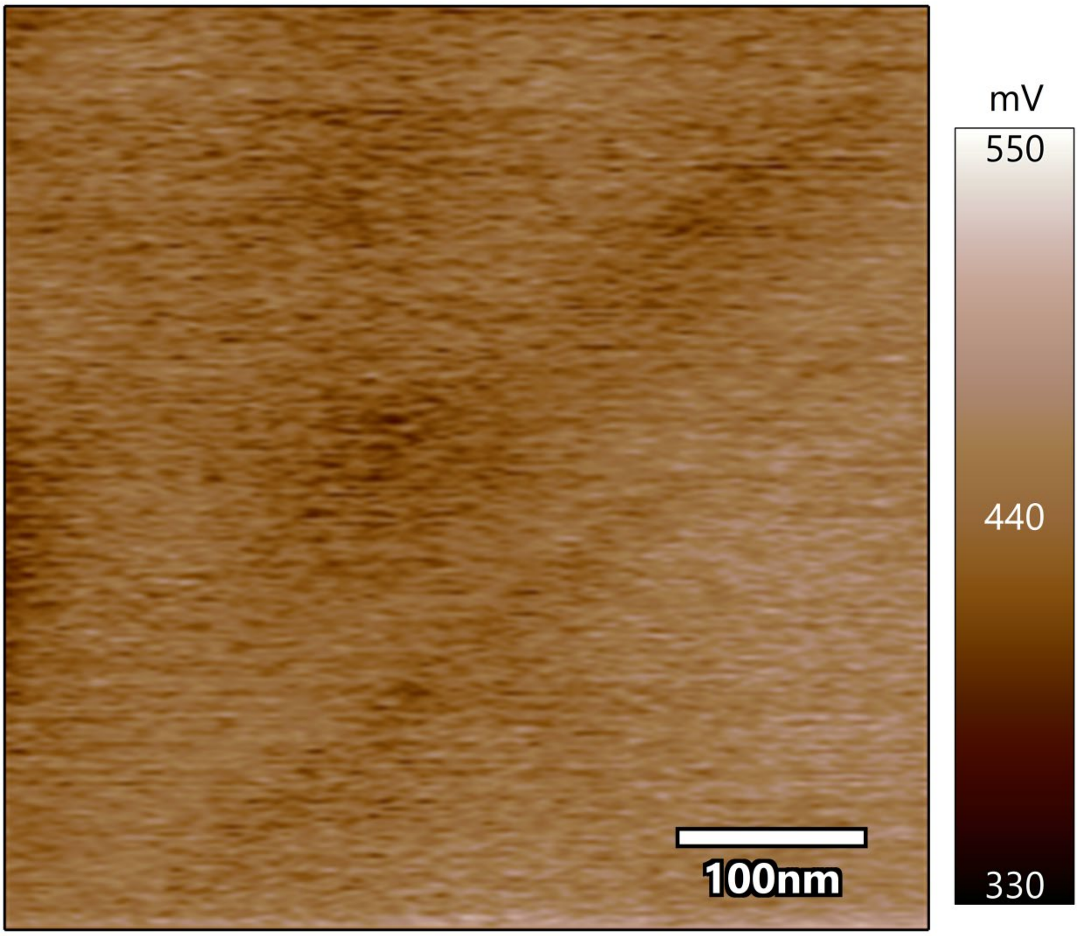

Supplementary Fig. 42 The surface potential of Ni-IH-7 measured by KPFM. Standard HOPG was measured as 0.675V. CPD was measured as 443.521mV.

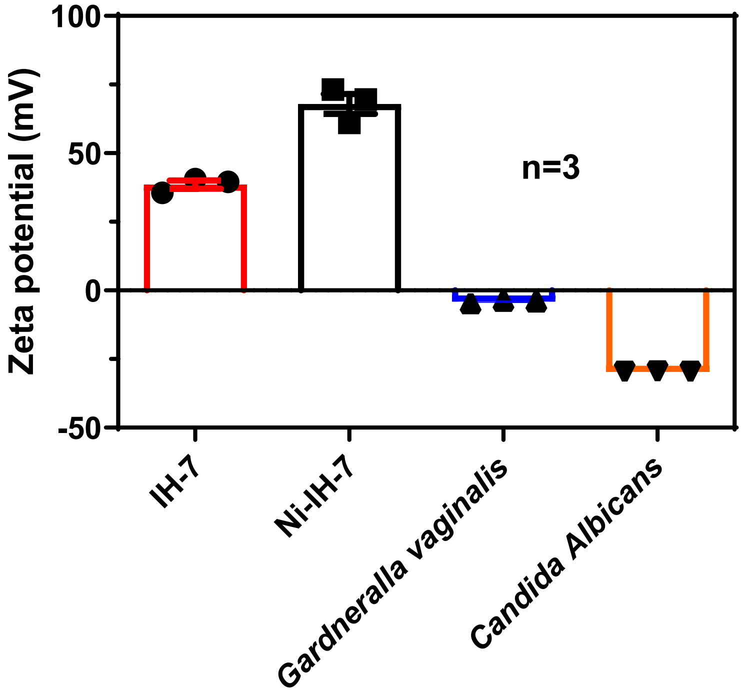

Supplementary Fig. 43 Zeta potential of Ni-IH-7, IH-7, *G. vaginalis* and *C. albicans*. n = 3 independent samples, bars represent means ± SD.

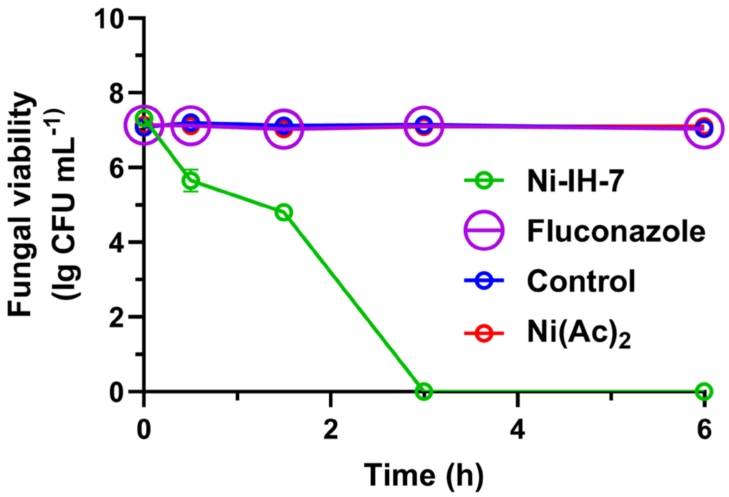

Supplementary Fig. 44 Antifungal activity of Ni-IH-7 nanotubes under different action time towards *C. albicans*. n = 3 independent samples, bars represent means ± SD.

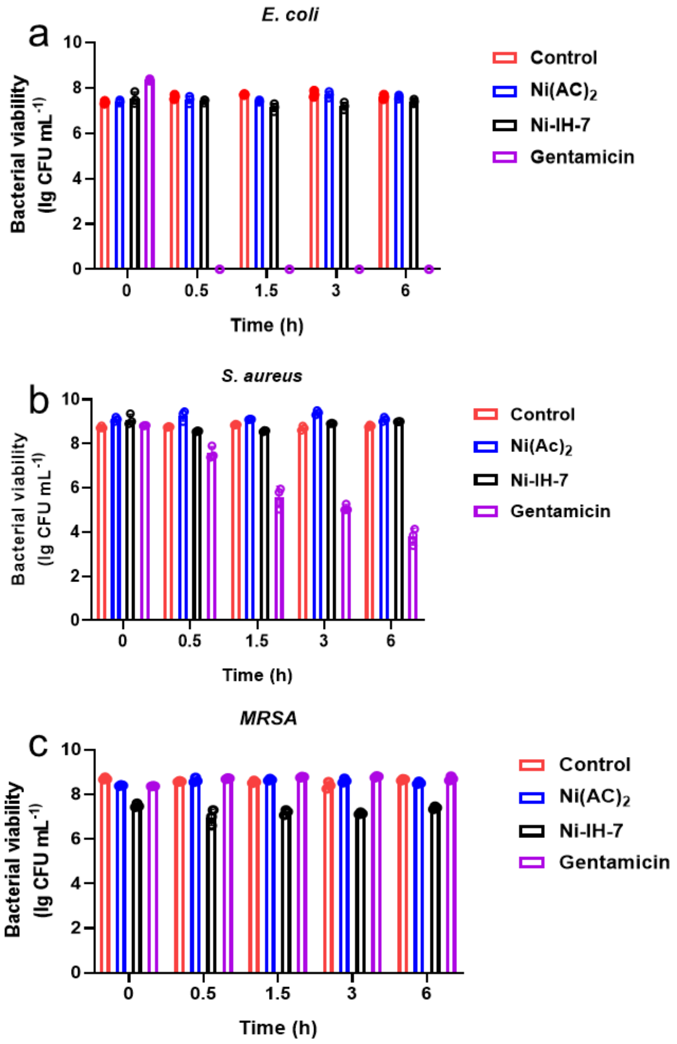

Supplementary Fig. 45 Antibacterial activity of Ni-IH-7 nanotubes under different action time towards (a) *E. coli*, (b) *S. aureus* and (c) *MRSA*. n = 3 independent samples, bars represent means ± SD.

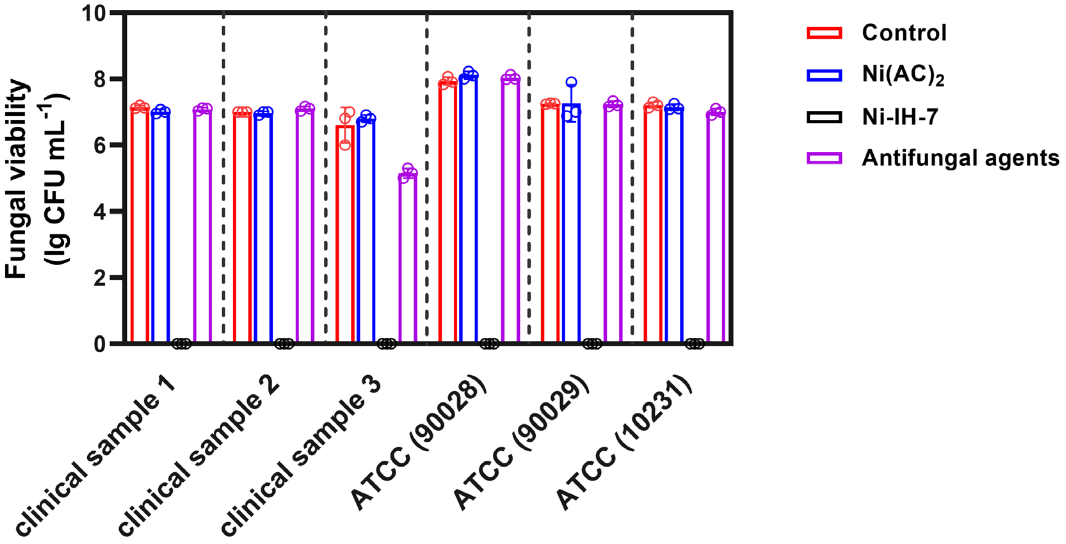

Supplementary Fig. 46 Antifungal activity of Ni-IH-7 nanotubes toward different isolates of *C. albicans*. Clinical samples were provided in hospital (Antifungal agents: clinical sample 1 and 2: 5-Flucytosine; clinical sample 3: Amphotericin B; ATCC (90028): Itraconazole; ATCC (90029): 5-Flucytosine; ATCC (10231): Fluconazole). n = 3 independent samples, bars represent means ± SD.

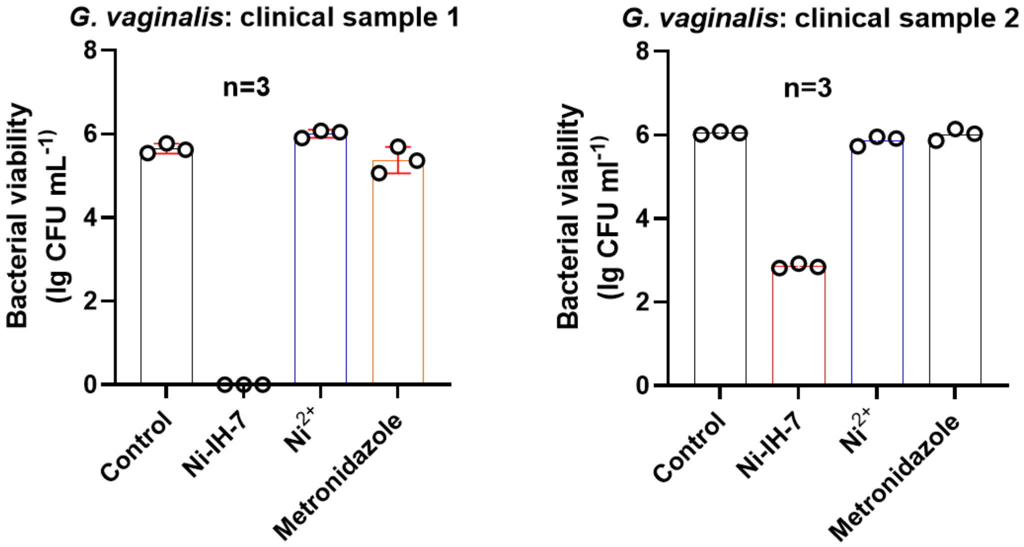

Supplementary Fig. 47 Antibacterial activity of Ni-IH-7 nanotubes toward different isolates of *G. vaginalis*. Clinical samples were provided in hospital (Antifungal agents: Metronidazole). n = 3 independent samples, bars represent means ± SD.

Supplementary Table 8 The antifungal names and the resistance degree purchased by ATCC.

ATCC	Anidu	Micafungi	Caspofungi	5-	Voriconazole	Itraconazol	Fluconazol

	lafung in	n	n	Flucytosine		e	e
10231	R	S	S	S	S	R	R
90028	S	S	S	S	S	DD-S	S
90029	S	S	S	R	S	S	S

R: resistant; S: susceptible; DD-S: Dose dependent-susceptible

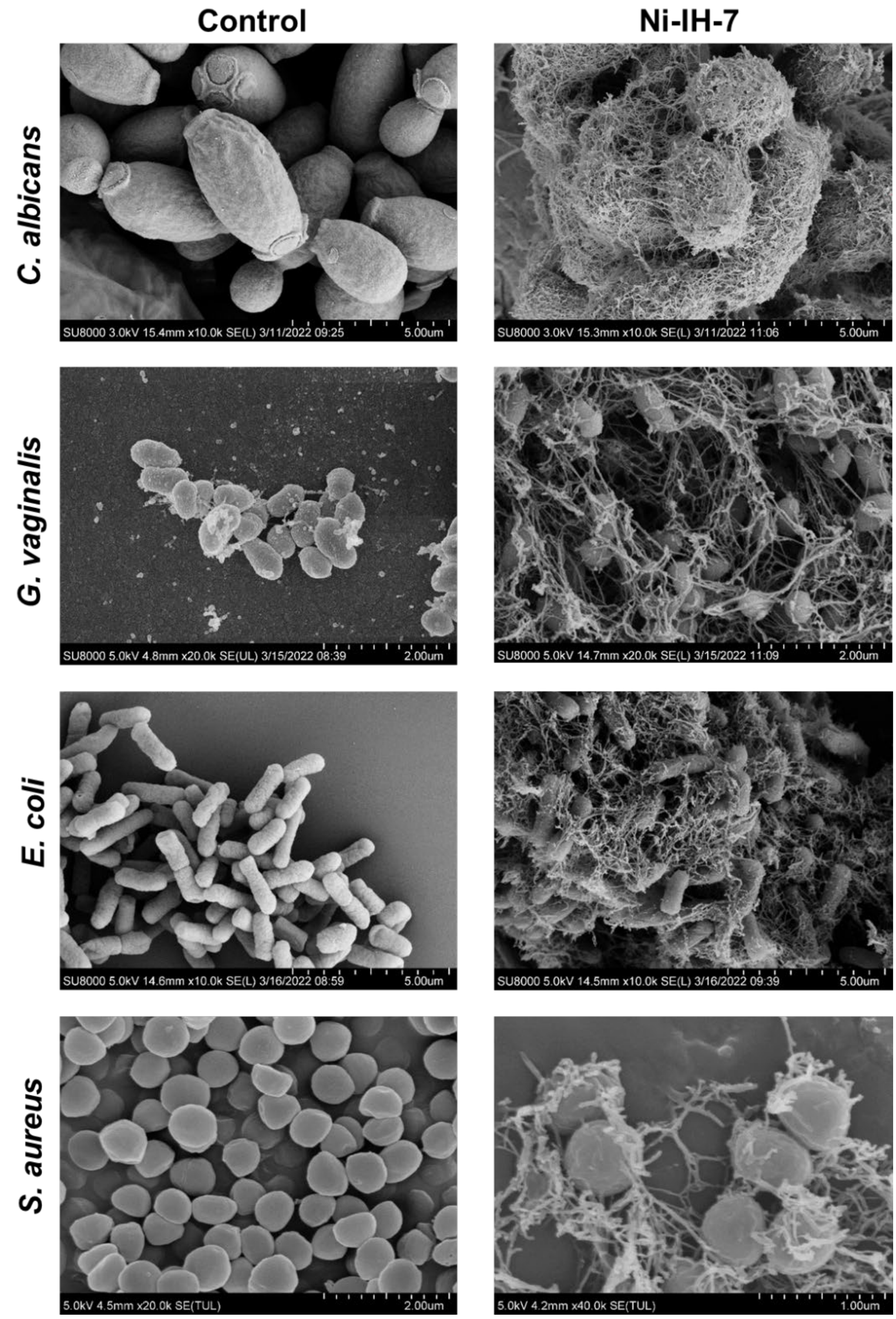

Supplementary Fig. 48 SEM characterization of Ni-IH-7 binding with different microbes. Three times each experiment was repeated independently with similar results. Representative images are shown.

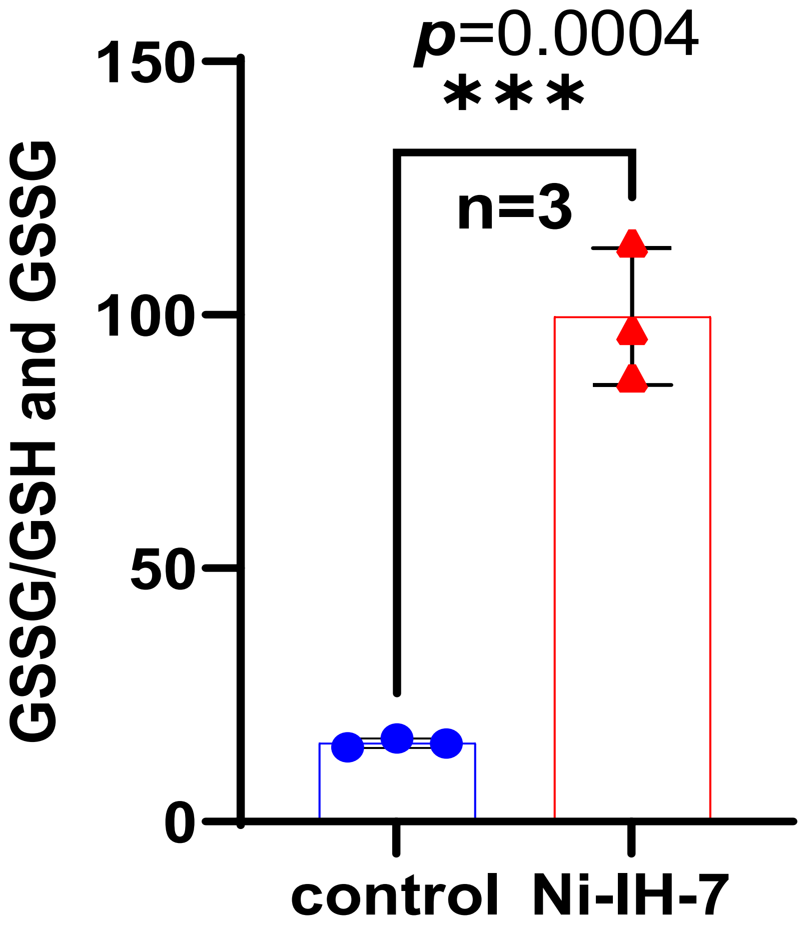

Supplementary Fig. 49 GSH ratio of *C. albicans* treated by Ni-IH-7. The significant difference was evaluated by a two-tailed unpaired t-test. n = 3 independent samples, bars represent means ± SD. ***$p < 0.001$.

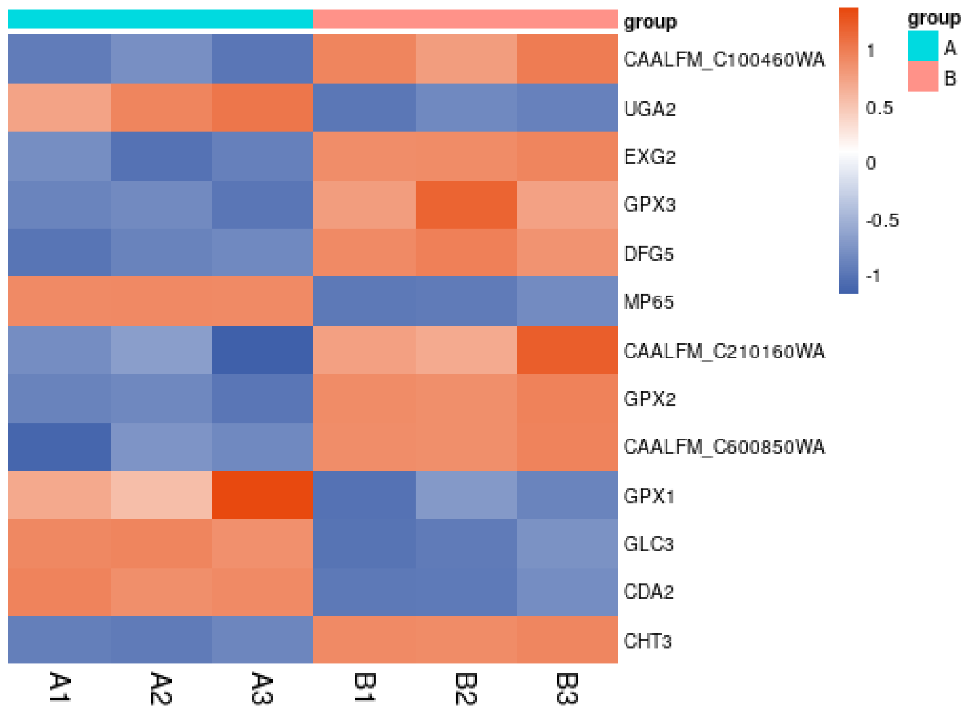

Supplementary Fig. 50 **Transcriptomic analysis of treated *C. albicans* by Ni-IH-7. A: control. B: Ni-IH-7.**

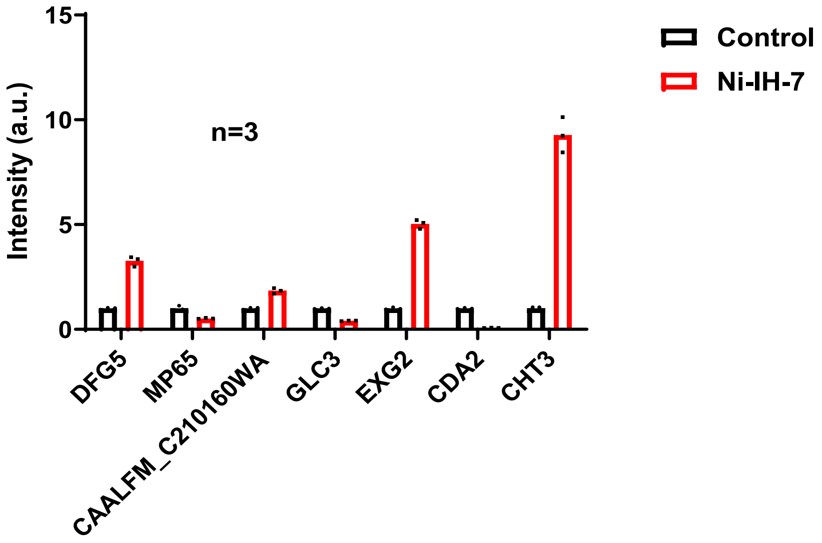

Supplementary Fig. 51 Real-time PCR verification of changes in cell wall-related genes. n = 3 independent samples, bars represent means ± SD.

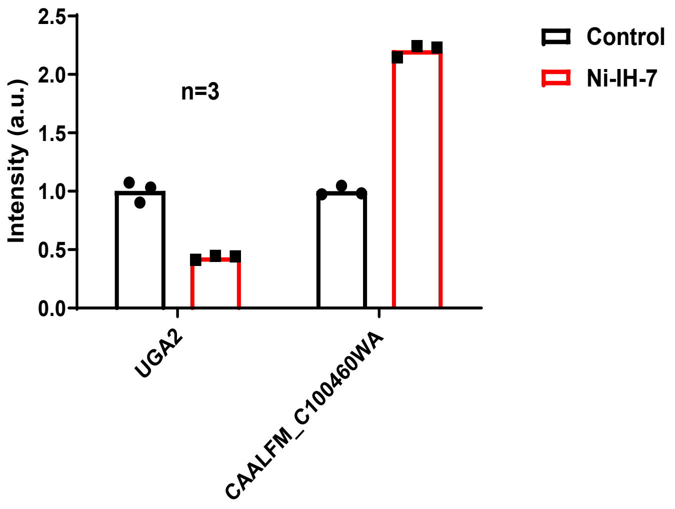

Supplementary Fig. 52 Real-time PCR verification of changes in cell membrane-related genes. n = 3 independent samples, bars represent means ± SD.

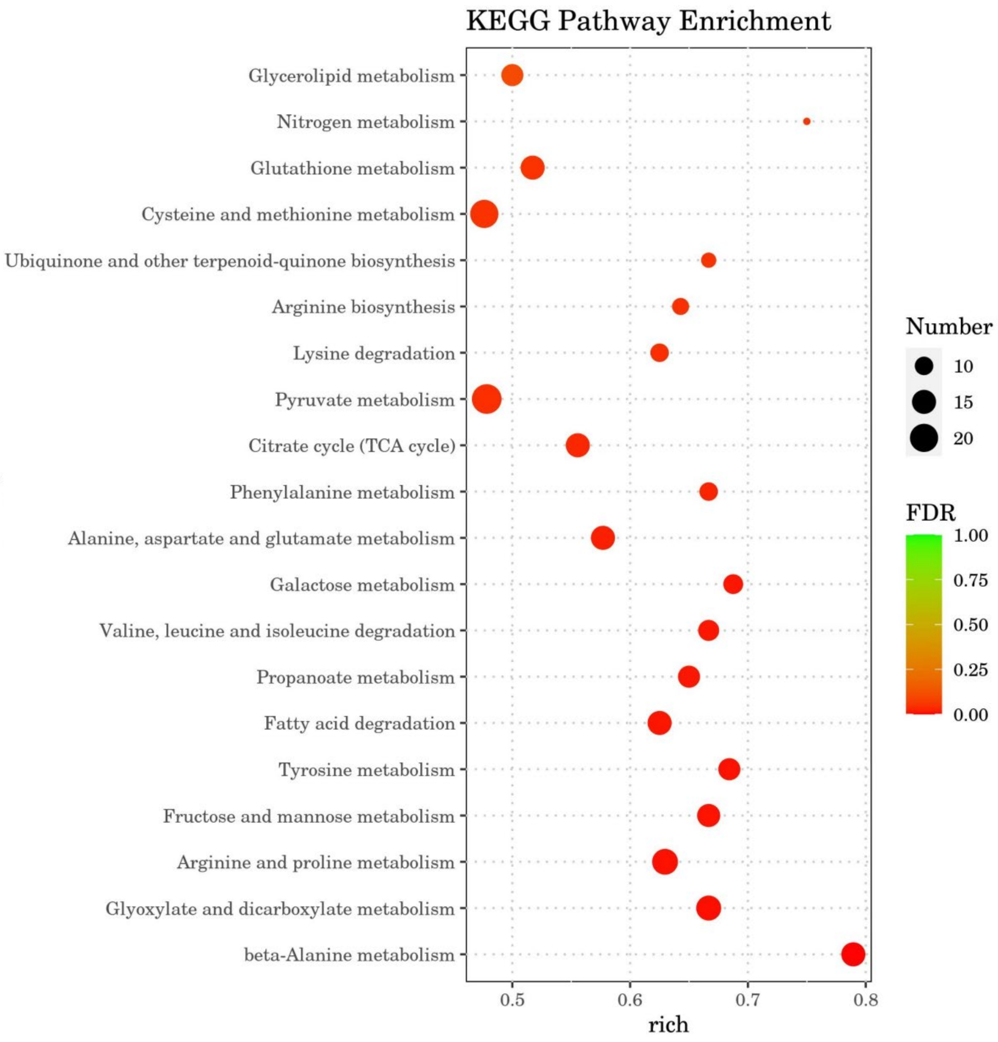

Supplementary Fig. 53 Metabolomic analysis of treated *C. albicans* by Ni-IH-7.

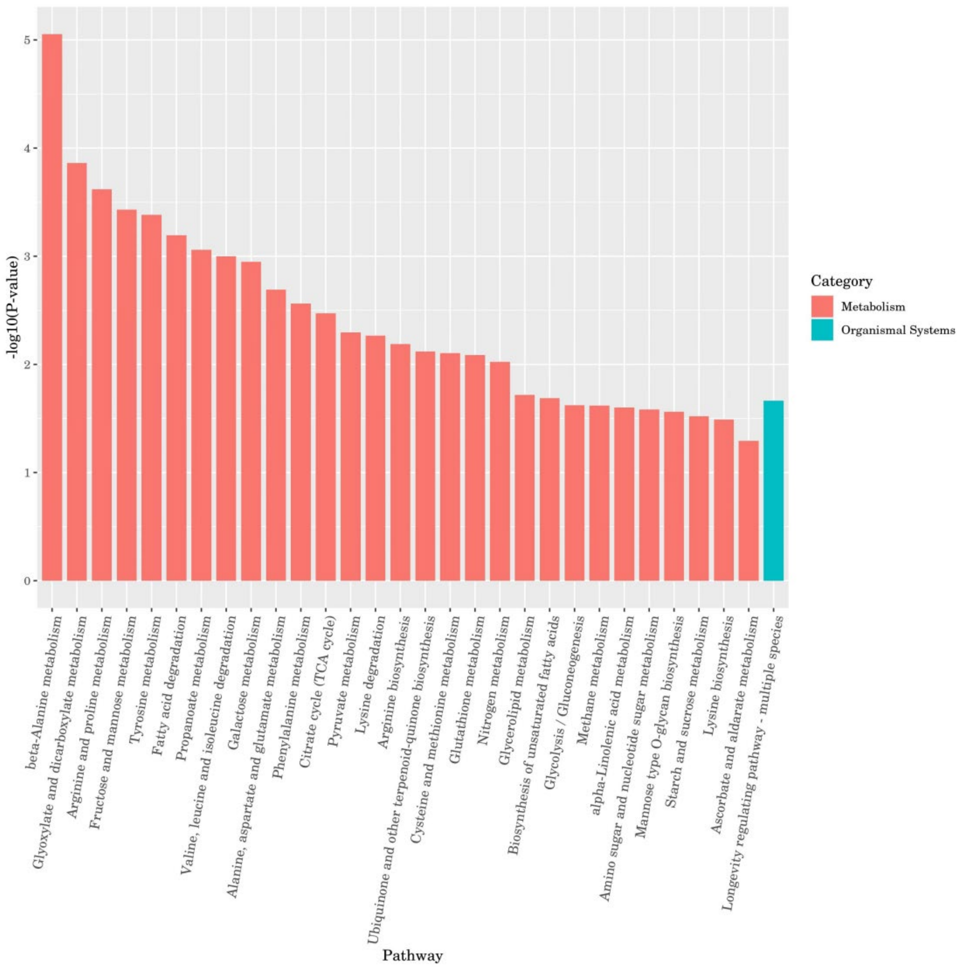

Supplementary Fig. 54 Metabolic analysis of *C. albicans* treated by Ni-IH-7.

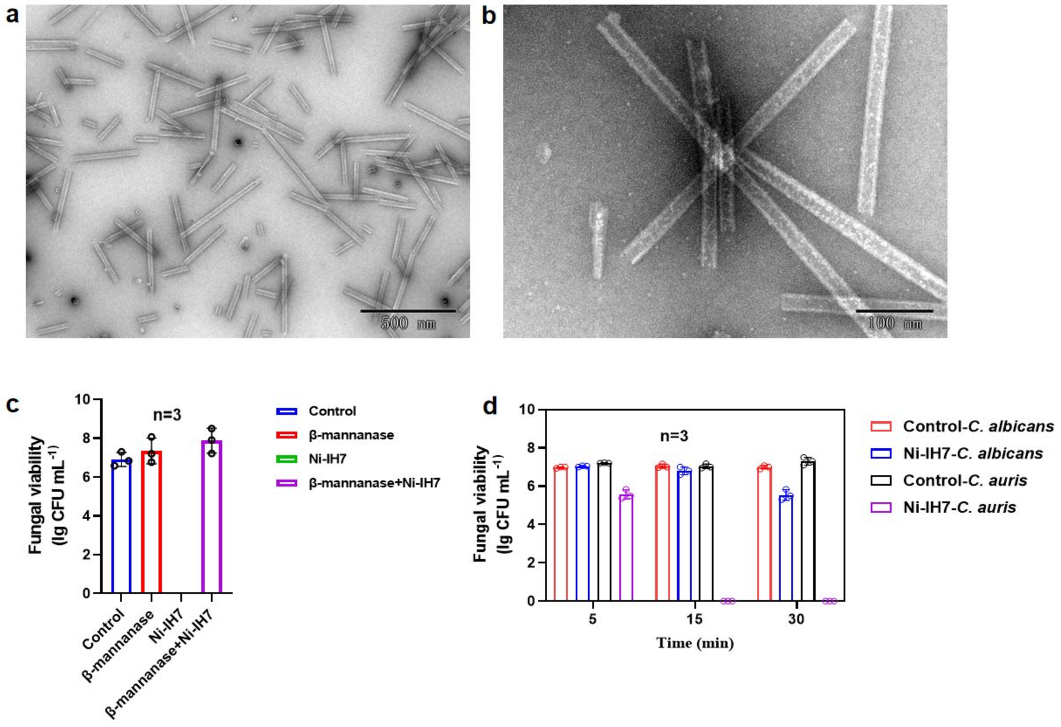

Supplementary Fig. 55 The relationship between antifungal effect of Ni-IH-7 nanotubes and mannan. **a** and **b**, TEM images of nanotubes after treatment with Ni-IH-7 and β-mannanase. **c**, Treatment of *C. albicans* with β-mannanase and nanotubes. **d**, Comparison of the killing abilities of Ni-IH-7 against *C. albicans* and *C. auris*. Three times each experiment was repeated independently with similar results. Representative images are shown. n = 3 independent samples, bars represent means ± SD.

Supplementary Table 9 The content of Mannan in different microbes.

Sample	Mannan (μg/10^8 CFU)
C. albicans	330.734
G. vaginalis	32.585
S. aureus	0.000
E. coli	0.722

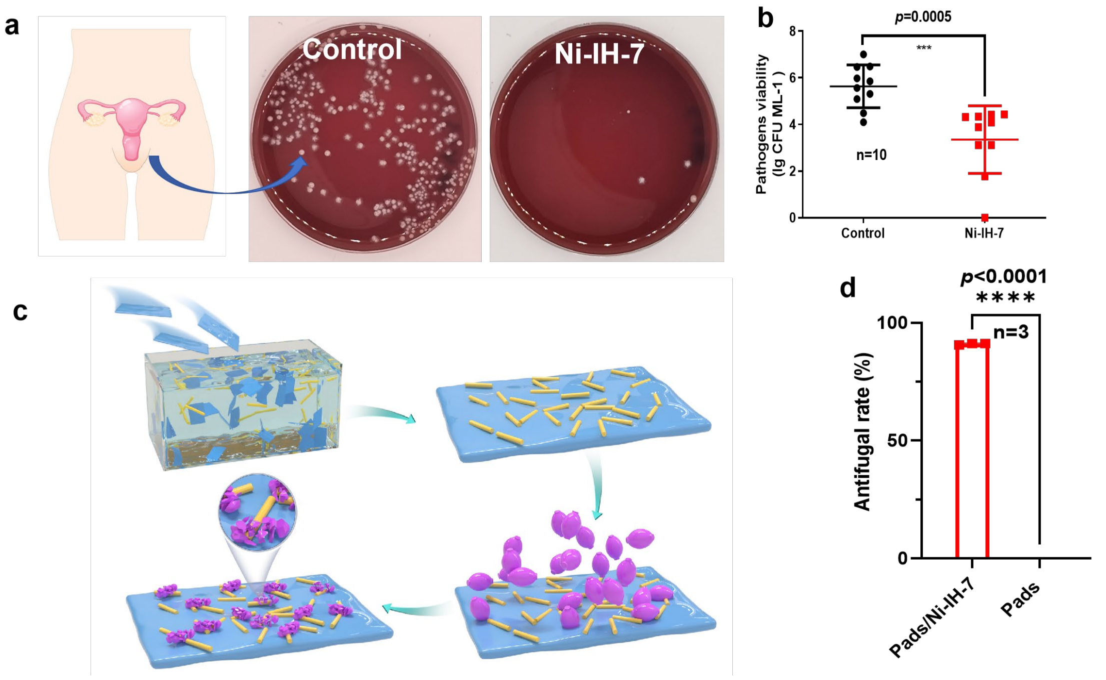

Supplementary Fig. 56 Antifungal assessment with Ni-IH-7 nanozymes. **a**, Anti-*C. albicans* effect of Ni-IH-7 nanozymes on the clinical samples from patients with vaginitis. **Left,** Schematic diagram of patient with fungal infection vaginitis. **Right,** The Photographed plates of antifungal test for the clinical samples treated by Ni-IH-7 nanozymes. **b,** Statistics of the antifungal efficiency of Ni-IH-7 nanozymes on the clinical vaginal secretion with fungal infection. n=10. Mean ± SD are shown, ***p < 0.001. **c,** Schematic diagram of Ni-IH-7 nanozymes used in disinfection pad. **d,** Antifungal efficacy of disinfection pad functionalized with Ni-IH-7 nanozymes. The significant difference was evaluated by a two-tailed unpaired t-test. n = 3 independent samples, bars represent means ± SD. ***p < 0.001, and ****p < 0.0001.

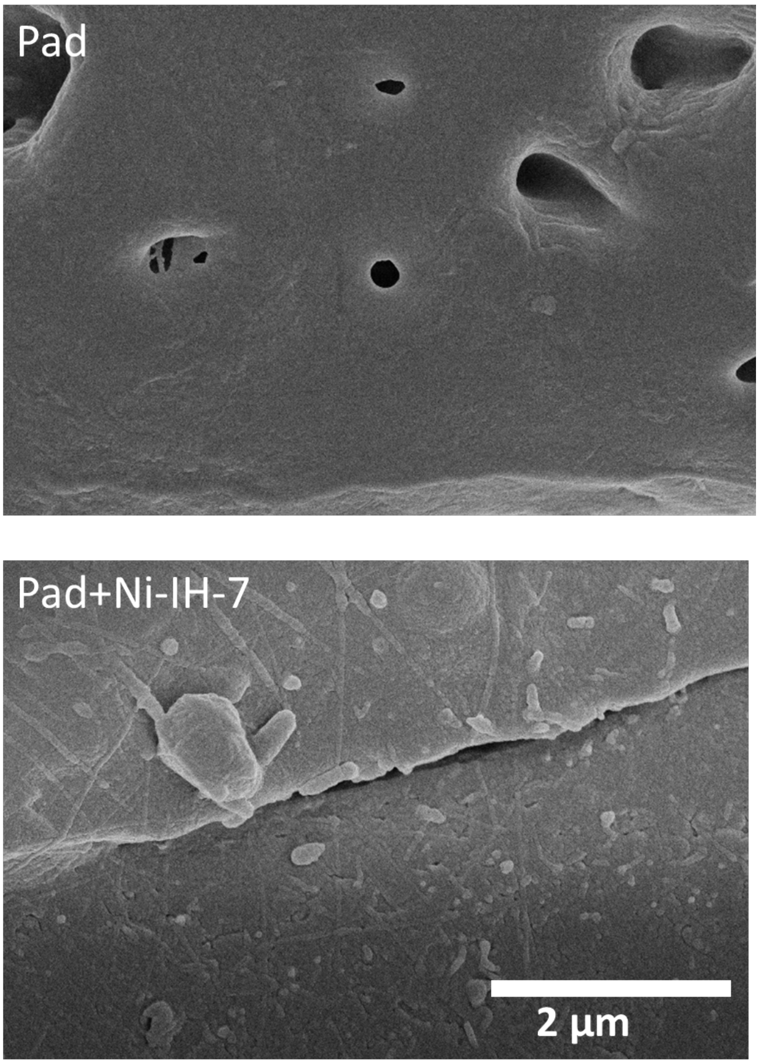

Supplementary Fig. 57 TEM characterization of medical pads bounded with Ni-IH-7 nanotubes. Three times each experiment was repeated independently with similar results. Representative images are shown.
