# Supplementary material for: Stable peptide-assembled nanozyme mimicking dual antifungal actions
Source: Nat Commun. 2024 Jul 5;15:5636. doi: 10.1038/s41467-024-50094-6 (PMC11224359; doi:10.1038/s41467-024-50094-6)
Supplement: Supplementary file 3 — Description of Additional Supplementary Files [file 41467_2024_50094_MOESM3_ESM.pdf]

Description of Additional Supplementary Files

File Name: Supplementary Data 1
Description: The primer sequences corresponding to glutathione peroxidase (GPX), cell wall-related, and cell membrane-related genes, respectively. TEF3 serves as the reference gene.

File Name: Supplementary Movie 1
Description: Animation of assembly of three heptapeptides.

File Name: Supplementary Movie 2
Description: Animation of assembly of six heptapeptides.
