# Supplementary material for: Stable peptide-assembled nanozyme mimicking dual antifungal actions
Source: Nat Commun. 2024 Jul 5;15:5636. doi: 10.1038/s41467-024-50094-6 (PMC11224359; doi:10.1038/s41467-024-50094-6)
Supplement: Supplementary file 7 — Reporting Summary [file 41467_2024_50094_MOESM7_ESM.pdf]

nature portfolio

Corresponding author(s): Lizeng Gao

Last updated by author(s): Jun 10, 2024

Reporting Summary

Nature Portfolio wishes to improve the reproducibility of the work that we publish. This form provides structure for consistency and transparency in reporting. For further information on Nature Portfolio policies, see our Editorial Policies and the Editorial Policy Checklist.

Statistics

For all statistical analyses, confirm that the following items are present in the figure legend, table legend, main text, or Methods section.

n/a	Confirmed	
☐	☒	The exact sample size (*n*) for each experimental group/condition, given as a discrete number and unit of measurement
☐	☒	A statement on whether measurements were taken from distinct samples or whether the same sample was measured repeatedly
☐	☒	The statistical test(s) used AND whether they are one- or two-sided<br>*Only common tests should be described solely by name; describe more complex techniques in the Methods section.*
☐	☒	A description of all covariates tested
☐	☒	A description of any assumptions or corrections, such as tests of normality and adjustment for multiple comparisons
☐	☒	A full description of the statistical parameters including central tendency (e.g. means) or other basic estimates (e.g. regression coefficient) AND variation (e.g. standard deviation) or associated estimates of uncertainty (e.g. confidence intervals)
☐	☒	For null hypothesis testing, the test statistic (e.g. *F*, *t*, *r*) with confidence intervals, effect sizes, degrees of freedom and *P* value noted<br>*Give P values as exact values whenever suitable.*
☒	☐	For Bayesian analysis, information on the choice of priors and Markov chain Monte Carlo settings
☒	☐	For hierarchical and complex designs, identification of the appropriate level for tests and full reporting of outcomes
☒	☐	Estimates of effect sizes (e.g. Cohen's *d*, Pearson's *r*), indicating how they were calculated

Our web collection on statistics for biologists contains articles on many of the points above.

Software and code

Policy information about availability of computer code

Data collection	Cryo-TEM and two-dimensional classification data were collected by Talos Arctica 200 kV FEG. DFT calculation was conducted using Gaussian09 package. MD simulations were using the software package Gromacs 5.1.5 or version 2020.6, PackMOL (18.002) was used to construct the self-assembled initial model. AutoDock 4.2 software was employed for molecular docking experiments.
Data analysis	GraphPad Prism 9.0.0

For manuscripts utilizing custom algorithms or software that are central to the research but not yet described in published literature, software must be made available to editors and reviewers. We strongly encourage code deposition in a community repository (e.g. GitHub). See the Nature Portfolio guidelines for submitting code & software for further information.

Data

Policy information about availability of data

All manuscripts must include a data availability statement. This statement should provide the following information, where applicable:

- Accession codes, unique identifiers, or web links for publicly available datasets
- A description of any restrictions on data availability
- For clinical datasets or third party data, please ensure that the statement adheres to our policy

Data availability. All data are available in the main text or Supplementary Materials. All electron microscope images are available upon request to the corresponding authors. Source data are provided in this paper.

Research involving human participants, their data, or biological material

Policy information about studies with human participants or human data. See also policy information about sex, gender (identity/presentation), and sexual orientation and race, ethnicity and racism.

Reporting on sex and gender	Female. The ages of human participants are 35, 42, 46, 48, 27, 36, 41, 31, 26, and 47, respectively.
Reporting on race, ethnicity, or other socially relevant groupings	Patients with fungal infection vaginitis.
Population characteristics	Patients with fungal infection vaginitis.
Recruitment	10 samples of vaginal secretions.
Ethics oversight	his study was approved by the Ethics Committee of Wuxi Xishan People's Hospital (approval number: LLS2020ky036 27/07/2020) and obtained informed consent from all patients.

Note that full information on the approval of the study protocol must also be provided in the manuscript.

Field-specific reporting

Please select the one below that is the best fit for your research. If you are not sure, read the appropriate sections before making your selection.

- [x] Life sciences
- [ ] Behavioural & social sciences
- [ ] Ecological, evolutionary & environmental sciences

For a reference copy of the document with all sections, see nature.com/documents/nr-reporting-summary-flat.pdf

Life sciences study design

All studies must disclose on these points even when the disclosure is negative.

Sample size	The in vitro experiments were independently performed three times and standard number of replication is three in all in vitro studies.
Data exclusions	No data were excluded.
Replication	Experiments were repeated and experimental findings were reproducible.
Randomization	Samples were randomly allocated to corresponding experimental groups.
Blinding	The experiments and results analyses were performed by multiple researchers, who had minimal information of sample identification. However, samples were not formally blinded.

Reporting for specific materials, systems and methods

We require information from authors about some types of materials, experimental systems and methods used in many studies. Here, indicate whether each material, system or method listed is relevant to your study. If you are not sure if a list item applies to your research, read the appropriate section before selecting a response.

Materials & experimental systems

n/a	Involved in the study	
x		Antibodies
	x	Eukaryotic cell lines
x		Palaeontology and archaeology
	x	Animals and other organisms
x		Clinical data
x		Dual use research of concern
x		Plants

Methods

n/a	Involved in the study	
x		ChIP-seq
x		Flow cytometry
x		MRI-based neuroimaging

Eukaryotic cell lines

Policy information about cell lines and Sex and Gender in Research

Cell line source(s)	The VK2 cell line was obtained from ATCC.
Authentication	The cell line was not independently authenticated.
Mycoplasma contamination	All cell lines tested for mycoplasma contamination by the supplier.
Commonly misidentified lines (See ICLAC register)	No commonly misidentified cell lines were used.

Animals and other research organisms

Policy information about studies involving animals; ARRIVE guidelines recommended for reporting animal research, and Sex and Gender in Research

Laboratory animals	10 week old c57BL/6 rats, (all animals were housed in a temperature controlled (25 ± 2 °C) condition (treatment of humidity from 40-70%) with normal light, and were given free access to water and diet).
Wild animals	The study did not involve wild animals.
Reporting on sex	n/a
Field-collected samples	The study did not involve samples collected from the field
Ethics oversight	All animal procedures were performed in accordance with the Guidelines for Care and Use of Laboratory Animals of Zhengzhou University and experiments were approved by the Animal Ethics Committee of Zhengzhou University.

Note that full information on the approval of the study protocol must also be provided in the manuscript.

Plants

Seed stocks	n/a
Novel plant genotypes	n/a
Authentication	n/a
